# Machine learning prediction of dual absorber lead-free perovskite solar cells for boosting PCE

**DOI:** 10.1038/s41598-026-51970-5

**Published:** 2026-05-24

**Authors:** Shorok Elewa, Nihal F. F. Areed, Bedir Yousif, Mohy Eldin A. Abo-Elsoud

**Affiliations:** 1 Electrical Engineering Department, Faculty of Engineering, Kafrelsheikh University, Kafrelsheikh, 33516 Egypt; 2 Department of Electrical Engineering, College of Engineering, Qassim University, 52571 Buraydah, Saudi Arabia; 3 Department of Electrical Engineering, College of Engineering and Information Technology, Onaizah Colleges, 56447 Qassim, Saudi Arabia; 4 Electronics and Communications Department, Faculty of Engineering, Mansoura University, Mansoura, 35516 Egypt

**Keywords:** Bi-layer, Double perovskites, Lead-free, Machine learning, Photovoltaics, PSCs, RF, Solar cells, XGB, Energy science and technology, Engineering, Materials science

## Abstract

Curtailing the toxicity level of perovskites is a considerable obstacle resisting the wide-scale commercialization of perovskite solar cells (PSCs). This study investigates the impact of implementing several charge transport layers (CTLs) on the performance of the proposed lead-free Cs_2_TiCl_6_/ Cs_2_AgBiI_6_ PSC employing SCAPS-1D simulations. Additionally, the effect of variations in thickness, doping, and defect concentrations of each layer has been considered to optimize the performance of the proposed device. Furthermore, various machine learning models have been trained to estimate the performance of the proposed device through a generated dataset consisting of $2187$ unique data points. Results reveal that employing high quality Cs_2_TiCl_6_ layer of $100\ nm$ thickness and $1\times {10}^{14\ } {cm}^{-3}$ donor doping density, above a $1000\ nm$ Cs_2_AgBiI_6_ absorber with $1\times {10}^{18\ } {cm}^{-3}$ acceptor doping density can theoretically achieve a power conversion efficiency (PCE) of $32.72 \%$ and a short circuit current density (J_SC_) of $26.06\ mA/{cm}^{2}$. Moreover, the extreme gradient boosting (XGB) model has been demonstrated to be the most effective model to predict the performance of the proposed PSC, yielding the lowest root mean square error ($RMSE$), and the highest coefficient of determination (${R}^{2}$) among the other examined models. The findings highlight the capability of optimally engineered dual-absorber PSCs to be considered as eco-friendly, competitive alternatives to the conventional lead-based PSCs.

**Supplementary Information:**

The online version contains supplementary material available at 10.1038/s41598-026-51970-5.

## Introduction

Owing to their extraordinary optoelectronic characteristics, lead-based halide perovskites, which contain Pb as metal cation, have been introduced to the optoelectronics field for use in many applications such as lasers, light emitting diodes, photodetectors, and solar cells (SCs) ^1,2^. Specifically, single-absorber Pb-based perovskite solar cells (PSCs) have recently achieved a power conversion efficiency (PCE) of $27.3 \%$, while tandem PSCs have accomplished PCE of $30.1 \%$ ^3,4^. These remarkable PCE values can be attributed to the high absorption, in addition to their superior lifetimes of carriers, long diffusion lengths, escalated carrier mobility, and tunable bandgap energies, which enable superior light absorption and moderate transport and extraction of carriers. In addition, the lightweight, flexible nature of these materials makes them ideal alternatives to replace silicon-based SCs ^5^. However, the large-scale manufacturing of Pb-based PSCs is considered to be limited compared with other types of SCs, because of the toxic nature of lead and the concerns related to instability and degradation of performance of perovskite materials upon exposure to heating, illumination, or humidity ^6^. A practical solution that has been introduced to reduce the toxicity and performance degradation issues of Pb-based perovskites is replacing the lead cations with metals of IVA and VA groups such as germanium (Ge), tin (Sn), bismuth (Bi) and antimony (Sb) as their ions have comparable structural and chemical properties to Pb ^7^. Yet, tin-based SCs face chemical instability issues, as Sn^2+^ can be oxidized to Sn^4+^ upon exposure to moisture and oxygen, which can increase the formation of defects and in turn deteriorate the device performance ^8^.

Recently, several research efforts have been focused on lead-free double perovskites. The cubic crystal structure and properly tuned bandgaps of double perovskites offer the potential for these materials to be utilized as a replacement for unstable, toxic Pb-based PSCs ^9^. Double perovskites, with structures that can be expressed as A_2_B^+^B^3+^X_6_, utilize monovalent and trivalent cations in the same structure. This combination of cations not only can improve the material’s stability, but it also can result in variations in its optoelectronic characteristics, based on the arrangement of the ions ^10^. In addition, these materials reveal optoelectronic behaviors which are remarkably similar to inorganic halide perovskites, highlighting their potential in next-generation photovoltaic applications ^11^. In this context, Chen et al. introduced a cesium titanium bromide (Cs_2_TiBr_6_) PSC that achieved stable performance with a PCE of $3.22 \%$ and diffusion lengths exceeding $100\ nm$ for both electrons and holes, utilizing P3HT as an organic hole transport layer (HTL) and a dual electron transport layer (ETL) of TiO_2_ and C60 ^12^. Utilizing cesium silver bismuth bromide (Cs_2_AgBiBr_6_) as main absorber with SnO_2_ and Cu_2_O as charge transport layers (CTLs) escalated PCE to 1.52% as reported in the work of Xiao and his colleagues ^13^. Another Titanium-based PSC introduced by Islam and Paul has been proved to theoretically achieve PCE of $27.36 \%$ employing PIN structure in which P-region was selected to be Cs_2_TiBr_6_ while the N and I layers were chosen to be cesium titanium chloride (Cs_2_TiCl_6_) ^14^. Hossain et al. reported a combined density functional theory and finite difference analysis based study that indicates that utilizing cesium silver bismuth iodide (Cs_2_AgBiI_6_) double perovskite as absorber with the use of ZnO as ETL can theoretically achieve PCE of $21.59 \%$ ^15^.

Although the efficiency of single-absorber SCs has improved significantly, the performance of such cells is inherently restricted by the Shockley-Queisser limit and thermodynamic principles. Various light management techniques have been introduced to elevate the functionality of single-absorber SCs including antireflection coating ^16^, surface texturing ^17^, and plasmonic nanostructures ^18^. However, producing these light trapping structures may require complex and carefully executed fabrication steps to realize the targeted optical characteristics ^19^. Another design technique that can be used to elevate the performance of SCs is the employment of tandem structure. Employing tandem structures by stacking higher bandgap cell above lower bandgap one can increase the range of absorbed wavelengths as the upper sub-cell can absorb short-wavelength photons while the lower sub-cell can absorb the photons of higher wavelengths. This configuration can lessen both thermalization and transmission losses and in turn can enhance efficiency of the device ^20^. Nevertheless, the employment of full tandem SCs is limited because of the high cost fabrication and the requirement of current matching between the sub-cells ^21^. Half-tandem SCs, on the other hand, can provide a practical and more cost-effective alternative. The direct stacking of dual absorber layers without additional interfacial layers enhances light absorption and supports efficient charge transport, which results in an escalated PCE ^22^. In this context, Zhu et al. confirmed the ability of dual-layer perovskite to enhance the device stability ^23^. In addition, Lin et al. reported 2D/3D bilayer PSC that successfully achieved high stability and enhanced performance with a PCE of $28.5 \%$, utilizing a layer of lead-halide perovskite atop a layer of mixed lead–tin (Pb–Sn) perovskite as main absorbers ^24^. Hasnain et al. introduced a numerical study which stated that utilizing PSC of structure ITO/ C60/ MASnI_3_/ RbGeI_3_/ Cu_2_O/ Au can accomplish a $20.19 \%$ PCE and an escalated short circuit current density (J_SC_) of approximately $32.7\ mA/c{m}^{2}$ ^25^. The bi-absorber PSC configuration proposed by Dev et al. effectively attained a high PCE of $30.58 \%$ due to the optimization of optoelectrical characteristics of CsSnI_3_ and Cs_3_Sb_2_Br_9_ absorbers ^20^.

Currently, integrating machine learning (ML) approaches into the design of SCs helps to overcome experimental limitations by identifying complex relationships between material characteristics and device performance in the form of simple correlations, which in turn can enhance predictive modeling ^26^. While some ML models cannot effectively predict the same experimental results of PSCs because of the multifaceted correlations in manufacturing conditions, the analysis of these models facilitates reverse-engineering approaches for the design of high-performance PSCs. In this regard, recent studies emphasize an increasing shift toward combining ML with experimental data and stability-focused optimization. A dual-model ML framework was introduced by Zhu et al. to simultaneously optimize both stability and PCE of PSCs, using a high-quality dataset of device performance. Their adopted approach identified key fabrication strategies, highlighting passivation and additive engineering as the most influential factors for enhancing both stability and PCE. The experimental results validated that the devices prepared using the anti-solvent method can achieve a PCE of $25.03 \%$ and can preserve over $93 \%$ of their PCE during one week of maximum power point tracking ^27^. Additionally, a novel weighted integrated ML framework for the co-optimization of PCE and stability in tin-based PSCs was introduced by Muppana and colleagues. Their developed ML models were trained using experimentally obtained fabrication parameters to capture the complex interrelationships among the variables. This proposed approach achieved a correlation coefficient (${R}^{2}$) of $0.84$ for PCE prediction and $0.87$ for stability prediction ^28^. On the other hand, integrating ML with computational techniques enables researchers to efficiently analyze large datasets and optimize key physical parameters of solar cell designs without relying on extensive experimental testing. In this context, Shimul et al. applied a random forest (RF) ML technique to forecast the optimal photovoltaic parameters by considering key material properties such as layer thickness, bandgap, and carrier mobility, achieving a peak PCE of $23.7 \%$ and an ${R}^{2}$ of $0.94$ for PCE prediction ^29^. In addition, a linear regression (LR) ML model was trained by Khan et al. to evaluate how the physical properties of the tin-based perovskite layer may affect the performance of PSCs. Among the eight features considered within this study, the defect density of the absorber layer was identified as the most influential parameter, contributing 28.72% to the model and having a significant impact on the overall performance ^30^. Furthermore, Subudhi et al. proposed an ML study involving five different materials with different additives, applying several ML techniques, including LR, RF, support vector regression (SVR), extreme gradient boosting (XGB), and multilayer perceptron (MLP) algorithms, in order to accurately predict the key factors that affect the performance of PSCs. Among the evaluated models, RF yielded the highest ${R}^{2}$ value of $0.9999$, which implies the high accuracy of RF technique to predict the performance of tin-based devices ^31^. Biswas et al. also revealed that the RF algorithm can achieve higher accuracy than gradient boosting and decision tree regression algorithms in the forecasting of the optical performance of Sr_3_BiBr_3_-based PSCs, achieving an ${R}^{2}$ of $0.9973$ ^32^. Paul et al. also utilized an RF ML model to predict the performance of a Be_3_SbF_3_ solar cell, which demonstrated high accuracy with an ${R}^{2}$ value of $0.987$, in addition to identifying bandgap energy and defect density of absorber layer as the primary limiting factors for the PCE ^33^.

In this numerical study, a solar cell capacitance simulator (SCAPS-1D) dataset was generated through systematic variation of critical physical parameters, including absorber thickness, doping density, and defect concentration across both bulk and interface regions for a novel, unprecedented dual-absorber PSC structure utilizing Cs_2_TiCl_6_ and Cs_2_AgBiI_6_ as the main absorber layers. Subsequently, several ML models, including LR, SVR, RF, XGB, and K- nearest neighbors (KNN) algorithms were trained and systematically evaluated using k-fold cross validation to develop a reliable model for estimating the behavior of the proposed double perovskite bi-absorber based device. In addition, Shapley Additive Explanations (SHAP) were utilized to determine the correlation between absorber layers’ characteristics and device performance, which enables direct identification of dominant physical mechanisms governing device efficiency.

The rest of this article is organized as follows: Section "Materials and Methods" describes the main PSC structure proposed in this work, along with the optoelectrical parameters of the materials used, and outlines the modeling approach adopted in this study. Section "Results and Discussion" presents and discusses the results, while Section "Conclusion" concludes with the key findings and remarks of the study.

## Materials and methods

The electrical performance of the proposed lead-free dual-absorber device was examined in this study using SCAPS-1D, a one-dimensional SC computational modeling tool. The objective of this work is to determine the dual-absorber device structure which yields the optimal PCE and to highlight its advantages over conventional single-absorber PSCs. The following subsections present a detailed description of the introduced device and outline the adopted modeling framework.

### Device configuration

**Fig. 1:**
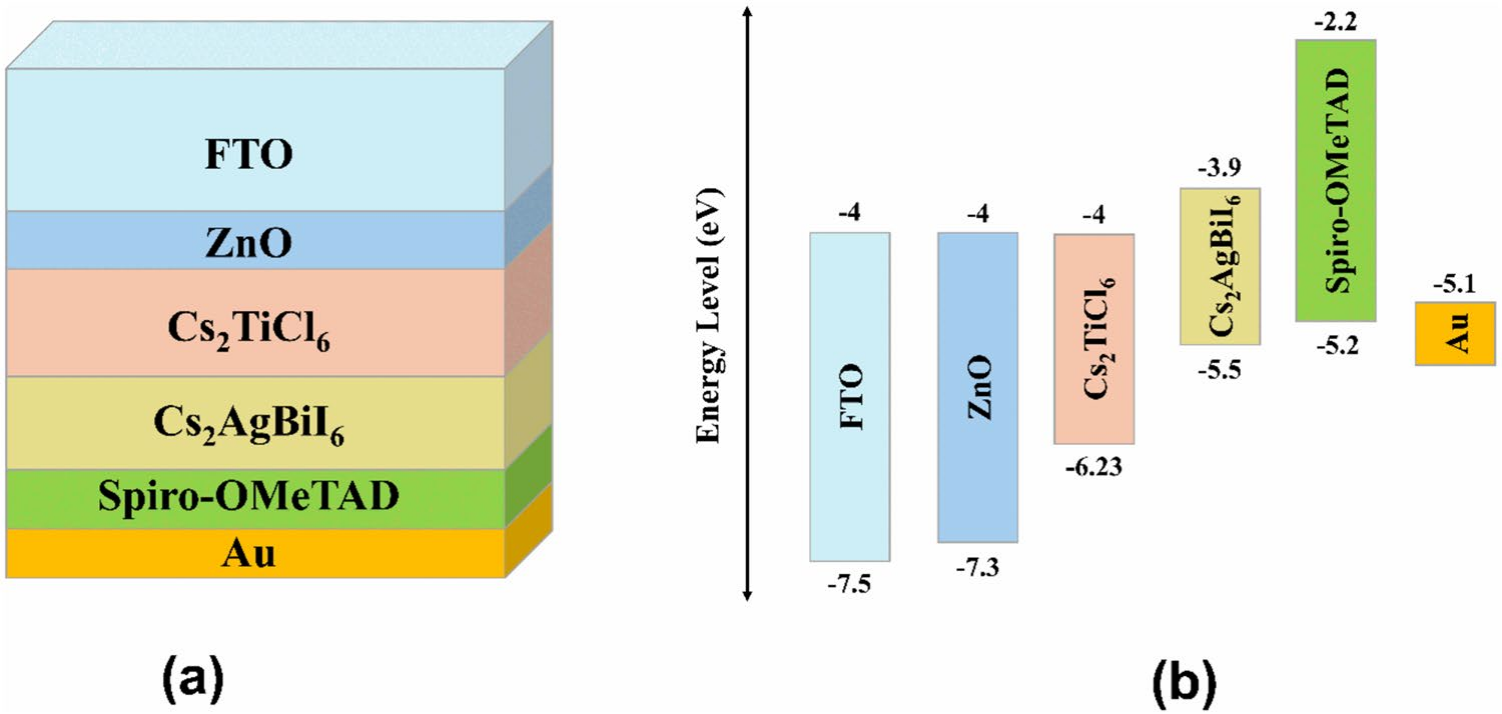
(**a**) Device structure (**b**) relative band alignment of initial proposed dual-absorber PSC.

The initial simulation of this study was executed on the bi-absorber nontoxic lead-free device with a structure presented in Fig. 1(a), composed of superimposed stack arranged as FTO/ ZnO/ Cs_2_TiCl_6_/ Cs_2_AgBiI_6_/ Spiro-OMeTAD/ Au. Fluorine-doped tin oxide (FTO) was selected to be the transparent conducting electrode. It allows light penetration into the device as well as assisting in the extraction of the photogenerated carriers. Zinc oxide (ZnO) was utilized as the ETL owing to its superior electrical properties ^34^. It facilitates electron collection while blocking hole transport. Hole transport layers, on the other hand, can facilitate the harvesting of generated holes while suppressing the backflow of electrons. In this study, Spiro-OMeTAD was initially chosen as the p-doped HTL. The p-doped cesium bismuth silver iodide (Cs_2_AgBiI_6_) layer and the n-doped cesium titanium chloride (Cs_2_TiCl_6_) layer serve as the dual-active absorbers, generating charge carriers upon exposure to light with sufficient energy. Gold was employed as the rear electrode in the initial structure because of its suitable work function, escalated conductivity, and robust stability ^35^.

The absorber sequence was selected according to the bandgap of the dual absorbers. A narrow-bandgap layer is integrated below a wide-bandgap absorber to harvest high-energy photons in the upper layer, whereas lower energy photons successfully penetrate it and get absorbed in the underlying narrow-bandgap layer ^20^. Furthermore, the bandgap alignment of the two absorbers was considered to ensure efficient electrical behavior of the suggested structure. A novel dual-absorber device design is introduced by this study, employing Cs_2_TiCl_6_ as a wide-bandgap material ($2.23\ eV$) and Cs_2_AgBiI_6_ as a narrow-bandgap absorber ($1.6\ eV$), to achieve an optimal bandgap arrangement for enhanced light harvesting and energy conversion. Figure 1(b) presents the bandgap diagram of the initial proposed PSC. As indicated by this figure, Cs_2_TiCl_6_ exhibits $-6.25\ eV$ highest occupied molecular orbital (HOMO) level, while that of Cs_2_AgBiI_6_ is $-5.5\ eV$. In addition, a deeper HOMO level of $-7.3\ eV$ was exhibited by the ZnO ETL, which efficiently allows the hole transport towards the HTL and prevent holes from moving in the other direction toward the transparent front electrode. Additionally, Cs_2_TiCl_6_ and Cs_2_AgBiI_6_ exhibit lowest unoccupied molecular orbital (LUMO) levels of $-4.02\ eV$ and $-3.9\ eV$, respectively, while Spiro-OMeTAD HTL exhibits a higher LUMO level of $-2.2\ eV$, which facilitates the electron flow toward the front contact. Based on this energy band configuration, the proposed PSC can exhibit optimal band structure that ensures efficient charge separation, which leads to enhanced device performance.

In the subsequent stages of this study, various charge transport layers were employed as HTLs including Cu_2_O, P3HT, PEDOT: PSS, CuI, CuO, CFTS, CBTS, PTAA, MoO_3_, MoS_2_, Cu_2_Te, nPB, and Sb_2_S_3_ while maintaining the device structure as presented in Fig. 1(a). Then the influence of changing the ETL to TiO_2_, PCBM, C_60_, IGZO, SnO_2_, WS_2_, SnS_2_, CdS, ZnSe, PC_60_BM, LBSO, Nb_2_O_5_, and CdZnS was investigated with the aim of establishing the most effective PSC design that achieves superior performance. The selection of CTLs for single or dual-absorber PSCs mainly relies on essential photovoltaic requirements such as optimal band alignment with the perovskite absorber, enhanced charge carrier mobility, reduced recombination losses, and strong chemical stability over time. Appropriate alignment of energy levels ensures efficient extraction of electrons and holes while minimizing interfacial recombination, thereby contributing significantly to improved PCE ^36,37^.

In addition, the impact of utilizing various back electrode materials was investigated with the aim of developing a more cost-effective device without compromising performance. Materials’ electrical characteristics of absorber and charge transport layers employed in this work were obtained from reported studies and entered manually into the section of material properties of the simulator. Tables 1 and 2 summarize the electrical characteristics of the materials and the interface characteristics utilized in the initial stage of this study.

**Table 1:** Material parameters employed in the simulation.

${\boldsymbol{P}}{\boldsymbol{a}}{\boldsymbol{r}}{\boldsymbol{a}}{\boldsymbol{m}}{\boldsymbol{e}}{\boldsymbol{t}}{\boldsymbol{e}}{\boldsymbol{r}}{\boldsymbol{s}}$	$\mathbf{F}\mathbf{T}\mathbf{O}$	$\mathbf{Z}\mathbf{n}\mathbf{O}$	${\mathbf{C}\mathbf{s}}_{2}\mathbf{T}\mathbf{i}{\mathbf{C}\mathbf{l}}_{6}$	${\mathbf{C}\mathbf{s}}_{2}\mathbf{A}\mathbf{g}\mathbf{B}\mathbf{i}{\mathbf{I}}_{6}$	$\mathbf{S}\mathbf{p}\mathbf{i}\mathbf{r}\mathbf{o}$ $-\mathbf{O}\mathbf{M}\mathbf{e}\mathbf{T}\mathbf{A}\mathbf{D}$
${\boldsymbol{T}}{\boldsymbol{h}}{\boldsymbol{i}}{\boldsymbol{c}}{\boldsymbol{k}}{\boldsymbol{n}}{\boldsymbol{e}}{\boldsymbol{s}}{\boldsymbol{s}}({\boldsymbol{n}}{\boldsymbol{m}})$	$100$	$50$	$100$	$700$	50
${{\boldsymbol{\varepsilon}}}_{{\boldsymbol{r}}}$	$9$	$9$	$11.9$	$6.5$	$3$
${{\boldsymbol{E}}}_{{\boldsymbol{g}}}$(${\boldsymbol{e}}{\boldsymbol{V}}$)	$3.5$	$3.3$	$2.23$	$1.6$	$3$
${\boldsymbol{\chi}}$(${\boldsymbol{e}}{\boldsymbol{V}}$)	$4$	$4$	$4$	$3.9$	$2.2$
${{\boldsymbol{N}}}_{{\boldsymbol{c}}}$(${{\boldsymbol{c}}{\boldsymbol{m}}}^{-3}$)	$2.2\times {10}^{18}$	$3.7\times {10}^{18}$	$1\times {10}^{18}$	$1\times {10}^{19}$	$2.2\times {10}^{18}$
${{\boldsymbol{N}}}_{{\boldsymbol{v}}}$(${{\boldsymbol{c}}{\boldsymbol{m}}}^{-3}$)	$1.8\times {10}^{19}$	$1.8\times {10}^{19}$	$1\times {10}^{19}$	$1\times {10}^{19}$	$1.8\times {10}^{19}$
${{\boldsymbol{\mu}}}_{{\boldsymbol{n}}}$/ ${{\boldsymbol{\mu}}}_{{\boldsymbol{p}}}$ (${{\boldsymbol{c}}{\boldsymbol{m}}}^{2}/{\boldsymbol{V}}{\boldsymbol{s}})$	$20/10$	$100/25$	$4.4/2.5$	$2/2$	$2.1\times {10}^{-3}$ $/2.16\times {10}^{-3}$
${{\boldsymbol{N}}}_{{\boldsymbol{A}}}$(${{\boldsymbol{c}}{\boldsymbol{m}}}^{-3}$)	-	-	-	$1\times {10}^{15}$	$1\times {10}^{18}$
${{\boldsymbol{N}}}_{{\boldsymbol{D}}}$(${{\boldsymbol{c}}{\boldsymbol{m}}}^{-3}$)	$1\times {10}^{18}$	$1\times {10}^{18}$	$1\times {10}^{16}$	-	-
${{\boldsymbol{N}}}_{{\boldsymbol{t}}}$(${{\boldsymbol{c}}{\boldsymbol{m}}}^{-3}$)	$1\times {10}^{15}$	$1\times {10}^{15}$	$1\times {10}^{15}$	$1\times {10}^{15}$	$1\times {10}^{15}$
**Ref**	^38^	^15^	^14,39^	^15^	^15^

**Table 2:** Parameters of the interface defects.

$\mathbf{P}\mathbf{a}\mathbf{r}\mathbf{a}\mathbf{m}\mathbf{e}\mathbf{t}\mathbf{e}\mathbf{r}\mathbf{s}$	$\mathbf{H}\mathbf{T}\mathbf{L}/{\mathbf{C}\mathbf{s}}_{2}\mathbf{A}\mathbf{g}\mathbf{B}\mathbf{i}{\mathbf{I}}_{6}$	${\mathbf{C}\mathbf{s}}_{2}\mathbf{T}\mathbf{i}{\mathbf{C}\mathbf{l}}_{6}/\mathbf{E}\mathbf{T}\mathbf{L}$
$\mathbf{Defect\ type}$	$\mathrm{N}\mathrm{e}\mathrm{u}\mathrm{t}\mathrm{r}\mathrm{a}\mathrm{l}$	$\mathrm{N}\mathrm{e}\mathrm{u}\mathrm{t}\mathrm{r}\mathrm{a}\mathrm{l}$
$\mathbf{Capture\ cross\ section\ of\ electrons\ } ({\mathbf{c}\mathbf{m}}^{2})$	$1\times {10}^{-18}$	$1\times {10}^{-18}$
$\mathbf{Capture\ cross\ section\ of\ holes\ } ({\mathbf{c}\mathbf{m}}^{2})$	$1\times {10}^{-19}$	$1\times {10}^{-19}$
$\mathbf{Energetic\ distribution }$	$\mathrm{S}\mathrm{i}\mathrm{n}\mathrm{g}\mathrm{l}\mathrm{e}$	$\mathrm{S}\mathrm{i}\mathrm{n}\mathrm{g}\mathrm{l}\mathrm{e}$
$\mathbf{Reference\ for\ defect\ energy\ level\ } {\mathbf{E}}_{\mathbf{t}}$	$\text{Above the highest } {\mathrm{E}}_{\mathrm{V}}$	$\text{Above the highest } {\mathrm{E}}_{\mathrm{V}}$
$\mathbf{Energy\ level\ with\ respect\ to\ reference\ }(\mathbf{e}\mathbf{V})$	$0.6$	$0.6$
$\mathbf{Total\ density} ({\mathbf{c}\mathbf{m}}^{3})$	$1\times {10}^{10}$	$1\times {10}^{10}$
**Ref**	^38^	^40^

The dielectric constant is denoted by ${\varepsilon}_{r}$, bandgap energy by ${E}_{g}$, and electron affinity by $\chi$. The valance band and conduction band effective densities of states are denoted by ${N}_{v}$ and ${N}_{c}$, respectively. Electron and hole mobilities are expressed as ${\mu}_{n}$ and ${\mu}_{p}$, while ${N}_{A}$ and ${N}_{D}$ represent the acceptor and donor doping densities. The defect density is given by ${N}_{t}$. Electrical parameters of the different HTLs and ETLs utilized in this analysis are indicated in Tables S1 and S2 of the Supplementary Information. Simulations were performed with the standard illumination (AM1.5G) at light intensity of $100\ mW/{cm}^{2}$ and $300 K$ as operating temperature. Subsequently, the effect of temperature changes on performance was studied.

### Numerical simulation

Version 3.3.12 of SCAPS-1D, which was introduced by Professor Marc Burgelmen ^41^, was utilized for the simulation of the proposed device. This software was selected for its advanced features, wide adoption in prior studies, and relative ease of operation compared to alternative programs used for SC simulation. The simulation framework is based on solving three fundamental differential equations: Poisson’s equation, the continuity equations, and the transport equations. Together these three equations can enable a comprehensive analysis of SC behavior ^42^. The relationship between charge distribution and the resulting electric field can be described by Poisson’s equation ^43^. On the other hand, the spatial change in electron and hole current densities can be expressed as functions of the charge carriers generation and recombination rates using continuity equations ^14^. Further, the transport equations describe the flow of charge carriers and current conduction in SCs ^29^.

The diffusion coefficient of holes and electrons ($${D}_{p}$$ and $${D}_{n})$$ can be determined as follows ^44^:

1 $${D}_{n}={\mu}_{n}\frac{KT}{q}$$

2 $${D}_{p}={\mu}_{p}\frac{KT}{q}$$

where $T$ and $K$ are the operating temperature and Boltzmann’s constant, respectively, and ${\mu}_{n}$ and ${\mu}_{p}$ denote the mobilities of electrons and holes, respectively. Diffusivity can be used to define how free charge carriers move in a material, which indicates their tendency to spread out from regions of high concentration. Additionally, the diffusion length describes how far the charge carriers typically migrate before recombination. The diffusion length of charge carriers can be expressed as follows ^45^:

3 $${L}_{p}=\sqrt{{D}_{p}{\tau}_{p}}$$

4 $${L}_{n}=\sqrt{{D}_{n}{\tau}_{n}}$$

here, the diffusion lengths of electron and hole are expressed as ${L}_{n}$ and ${L}_{p}$, and ${\tau}_{p}$ and ${\tau}_{n}$ represent the carrier lifetimes. A longer carrier lifetime can typically enhance device performance, as it enables a greater number of carriers to contribute to the current, and in turn enhances the overall performance of the device. The carrier lifetime primarily depends on the density of defects within each layer, and can be expressed by ^46^:

5 $$\tau =\frac{1}{\sigma {v}_{th} {N}_{t}}$$

Here, the thermal velocity of carriers is denoted by ${v}_{th}$ and their capture cross section is denoted by $\sigma$, and ${N}_{t}$ corresponds to defect density.

The optical absorption coefficient $\alpha (\lambda )$ can be considered as an essential parameter that represents the average penetration depth of photons with wavelength $\lambda$ entering a semiconductor of bandgap ${E}_{g}$ before being absorbed. SCAPS-1D provides several models for estimating this coefficient. The absorption coefficients of the Cs_2_AgBiI_6_ and Cs_2_TiCl_6_ absorber layers employed in this analysis were obtained from previously published studies ^15,47^ and incorporated into the material section of the simulation software. For the remaining layers of the proposed structure, however, the commonly adopted SC absorption model was employed ^39^.

### Machine learning modeling approach

ML, one of the artificial intelligence key disciplines, focuses on generating predictive models which can derive insights and patterns from large datasets. The rapid growth of computational capacity and data generation has significantly enabled the application of ML in several scientific fields. Recently, ML techniques have been utilized to analyze complex correlations among device parameters and photovoltaic performance, enabling prediction and guiding the design of next-generation SCs ^30^. Moreover, ML models can be used to identify correlations and determine optimal operating conditions by analyzing large-scale datasets that encompass operation and fabrication variables including temperature, pressure and humidity. These insights can contribute to reducing structural defects, improving film quality, and enhancing device efficiency and stability ^26^.

ML was applied in the present study to examine the influence of absorber layer characteristics on the effectiveness of introduced device by implementing ML models using Python. Python was chosen because of its robust available libraries which can be used for data processing, model training, and evaluation. A total of $2187$ output data points were obtained from SCAPS-1D simulations by varying the characteristics of both absorbers in a systematic manner. The dataset consisted of two components: input features, and target outputs. The input features included thickness, defect density, and doping concentration of both absorbers in addition to the density of interfacial defects between them. The target outputs were defined by PCE, J_SC_, open circuit voltage (V_OC_), and fill factor (FF) which are the key performance indicators of the proposed device. The dataset was divided into an $80 \%$ training subset and a $20 \%$ testing one to guarantee efficient model validation. Five different supervised ML algorithms (LR, SVR, KNN, RF, and XGB) were employed and systematically compared to determine the model that is capable of achieving the highest predictive precision for the target performance parameters. All analyses were performed within the Google Colab tool employing Scikit-learn (Sklearn) library and its related modules.

The choice of ML models in this study was driven by the bias-variance trade-off as well as the nature of the available dataset. Given that the dataset generated through SCAPS-1D simulations is moderately sized and structured, traditional ML methods are more suitable than deep learning approaches, which generally depend on large datasets to achieve reliable generalization and avoid overfitting ^26^. LR can be considered as a core simple supervised learning algorithm designed to estimate a continuous output variable from one or several predictor variables. Further, LR is characterized by high bias and low variance, serving as a reference model for identifying linear relationships between the input features and output variables. ^30^. On the other hand, KNN model determines the K closest data points to a given sample by evaluating distance metrics within the dataset and then predicts the target value by regression or classification according to the labels of those neighbors ^48^. SVR aims to determine the optimal hyperplane that minimizes prediction errors while maintaining model generalization by employing a regularization mechanism to control overfitting and utilizing a kernel function to model nonlinear dependencies between input and output variables ^31^. Both SVR and KNN models offer greater flexibility, reducing bias while maintaining a manageable level of variance, making them effective for modeling mild nonlinear patterns in datasets ^26^. The RF algorithm, on the other hand, is an ensemble model that aggregates the predictions of numerous decision trees. Each decision tree is trained on distinct bootstrap samples and feature subsets of the original dataset, and integrating these diverse estimators effectively mitigates overfitting tendencies, and enhances prediction stability ^26^. In addition, XGB can be considered as a powerful ensemble algorithm that applies gradient boosting by adding trees that correct the residuals of previous ones. XGB is well-suited for large-scale regression problems such as the estimation of photovoltaic metrics as it achieves high predictive performance and robustness due to the minimized loss function ^49^. Ensemble techniques such as RF and XGB can be utilized for their strong capability to reduce variance while capturing complex nonlinear interactions among physical parameters, achieved by bagging and boosting in RF and XGB, respectively, which enables more robust and accurate performance prediction ^26^. On the other hand, deep learning models were not adopted in this study as they typically exhibit high variance when trained on limited datasets and require significantly large amounts of data to ensure stable convergence and prevent overfitting ^26^. In addition, the interpretability offered by classical ensemble methods, particularly through SHAP analysis, can be considered as essential to link ML predictions with physically meaningful device parameters. Consequently, the selected models achieve an effective balance between predictive accuracy, generalization performance, and physical interpretability for the generated dataset employed in this study.

Two statistical performance metrics were employed to evaluate the prediction reliability of the applied analysis: the root mean square error ($RMSE$), and the correlation coefficient (${R}^{2}$). $RMSE$ measures the mean magnitude of prediction errors, while ${R}^{2}$ indicates the strength of the correlation between estimated and real values. Additionally, the mean absolute error (MAE) was calculated for each model. These parameters are formulated as mentioned in Table S3 of the Supplementary Information ^50^. Further, SHAP plots were employed to explain the ML model by quantifying the influence of each input feature on the predicted output which provides transparent insights into feature importance.

## Results and discussion

The results of this study were structured in distinct subsection, each addressing a particular aspect, including the impact of employing different charge transport layers, the effect of thickness and defect densities, and the impact of doping density on the performance of the proposed PSC. Additionally, the influence of the metal rear electrode and parasitic resistance on the effectiveness of the proposed PSC was investigated.

### Simulation results validation

The reliability of the employed model and the accuracy of the SCAPS-1D analysis introduced in this work were validated by reproducing the study on the fabricated device proposed by Lu et al. ^51^ and subsequently evaluating the simulated results against the published ones. The simulated J-V response of the single-absorber MAPbI_3_ device exhibits strong agreement with the experimental data, as the achieved accuracy is greater than $96 \%$. Only minor deviations were observed in the electrical performance, as J_SC_ slightly dropped from $22.9$ to $22.82\ mA/c{m}^{2}$, and PCE shifted from $20.8$ to $20.88 \%$. The minor reduction in the V_OC_ value from $1.15\ V$ in the reported results to $1.11\ V$ in the simulated results can be attributed to the simulator’s inability to capture all fabricated and environmental factors, even with the simulated structure being carefully adjusted to ensure strong agreement with reported findings. This performance demonstrates the reliability of the software. The complete input data and output electrical parameters are listed in Tables S5 and S6 of the Supplementary Information, respectively.

### Impact of utilizing different HTLs on device performance

**Fig. 2:**
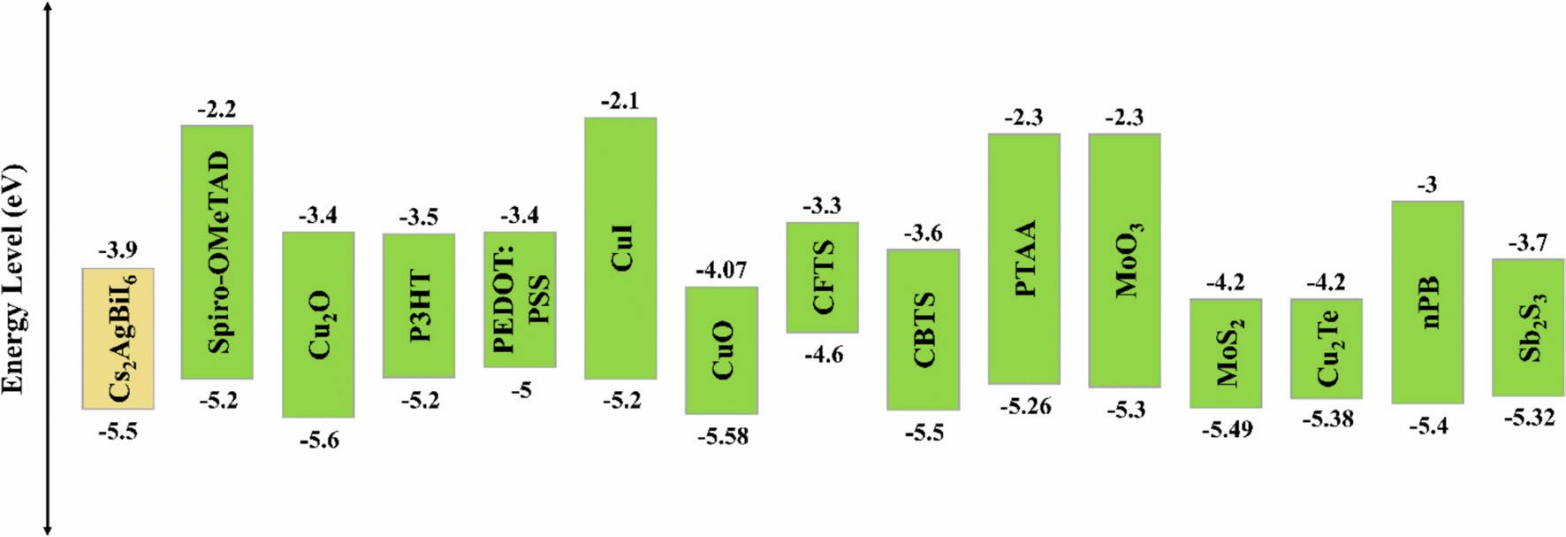
The alignment of energy bands of the different utilized HTLs with the lower Cs_2_AgBiI_6_ absorber.

Transport materials can play crucial roles in suppressing ion migration, preventing perovskite degradation, and protecting the absorber layers from moisture and oxygen during prolonged exposure to light, air, and heat. Therefore, the choice of charge transport materials with optimal characteristics may be considered a critical aspect for escalating both stability and PCE of the device. The study started with the selection of the optimal HTL using the initial device configuration shown in Fig. 1(a) by considering several widely employed HTLs such as Spiro-OMeTAD, Cu_2_O, P3HT, CuI, CuO, CFTS, CBTS, PTAA, MoO_3_, PEDOT: PSS, MoS_2_, Cu_2_Te, nPB, and Sb_2_S_3_. The key electrical characteristics of the utilized HTLs are provided in Table S1 of the Supplementary Information, while the corresponding energy band alignments with the lower Cs_2_AgBiI_6_ absorber are indicated in Fig. 2.

A systematic batch analysis was performed to identify the optimum HTL configuration, that improves the performance of the proposed device. The study examined the effect of thickness, acceptor, and defect concentrations of each utilized material on the effectiveness of the introduced device. Specifically, the HTL thickness was varied between $50$ and $500 nm$, the acceptor density ranged between $1\times {10}^{15}$ and $1\times {10}^{21\ }{ cm}^{-3}$, and the defect concentration ranged between $1\times {10}^{11}$ and $1\times {10}^{16}{\ cm}^{-3}$. Figures S1-S3 of the Supplementary Information present the effect of the examined parameters on the performance, whereas Table 3 reports the electrical characteristics associated with the maximum performance achieved when employing different HTLs with their optimized thickness, doping, and defect densities.

**Table 3:** Electrical parameters of introduced device corresponding to the maximum performance employing different HTLs.

$\mathbf{H}\mathbf{T}\mathbf{L}\mathbf{M}\mathbf{a}\mathbf{t}\mathbf{e}\mathbf{r}\mathbf{i}\mathbf{a}\mathbf{l}$	${\boldsymbol{T}}{\boldsymbol{h}}{\boldsymbol{i}}{\boldsymbol{c}}{\boldsymbol{k}}{\boldsymbol{n}}{\boldsymbol{e}}{\boldsymbol{s}}{\boldsymbol{s}}$ $({\boldsymbol{\mu}}{\boldsymbol{m}})$	${{\boldsymbol{N}}}_{{\boldsymbol{A}}}$ $({{\boldsymbol{c}}{\boldsymbol{m}}}^{-3})$	${{\boldsymbol{N}}}_{{\boldsymbol{t}}}$ $({{\boldsymbol{c}}{\boldsymbol{m}}}^{-3})$	${\boldsymbol{P}}{\boldsymbol{C}}{\boldsymbol{E}}(\boldsymbol{\%})$	${{\boldsymbol{J}}}_{{\boldsymbol{S}}{\boldsymbol{C}}}$ $({\boldsymbol{m}}{\boldsymbol{A}}/{{\boldsymbol{c}}{\boldsymbol{m}}}^{2})$	${{\boldsymbol{V}}}_{{\boldsymbol{O}}{\boldsymbol{C}}}$ $({\boldsymbol{V}})$	${\boldsymbol{F}}{\boldsymbol{F}}$ $(\boldsymbol{\%})$
${\boldsymbol{S}}{\boldsymbol{p}}{\boldsymbol{i}}{\boldsymbol{r}}{\boldsymbol{o}}-{\boldsymbol{O}}{\boldsymbol{M}}{\boldsymbol{e}}{\boldsymbol{T}}{\boldsymbol{A}}{\boldsymbol{D}}$	$0.50$	$1\times {10}^{21}$	$1\times {10}^{11}$	$20.43$	$22.79$	$1.09$	$82.01$
${{\boldsymbol{C}}{\boldsymbol{u}}}_{2}{\boldsymbol{O}}$	$0.50$	$1\times {10}^{21}$	$1\times {10}^{11}$	$20.51$	$22.90$	$1.10$	$81.66$
${\boldsymbol{P}}3{\boldsymbol{H}}{\boldsymbol{T}}$	$0.50$	$1\times {10}^{21}$	$1\times {10}^{11}$	$21.33$	$23.86$	$1.09$	$81.63$
${\boldsymbol{P}}{\boldsymbol{E}}{\boldsymbol{D}}{\boldsymbol{O}}{\boldsymbol{T}}:{\boldsymbol{P}}{\boldsymbol{S}}{\boldsymbol{S}}$	$0.50$	$1\times {10}^{21}$	$1\times {10}^{11}$	$19.90$	$24.35$	$1.09$	$75.02$
${\boldsymbol{C}}{\boldsymbol{u}}{\boldsymbol{I}}$	$0.50$	$1\times {10}^{21}$	$1\times {10}^{11}$	$20.47$	$22.79$	$1.09$	$82.08$
${\boldsymbol{C}}{\boldsymbol{u}}{\boldsymbol{O}}$	$0.50$	$1\times {10}^{21}$	$1\times {10}^{11}$	$20.58$	$22.85$	$1.10$	$82.18$
${\boldsymbol{C}}{\boldsymbol{F}}{\boldsymbol{T}}{\boldsymbol{S}}$	$0.50$	$1\times {10}^{21}$	$1\times {10}^{11}$	$14.28$	$29.79$	$0.74$	$64.66$
${\boldsymbol{C}}{\boldsymbol{B}}{\boldsymbol{T}}{\boldsymbol{S}}$	$0.50$	$1\times {10}^{21}$	$1\times {10}^{11}$	$20.96$	$23.25$	$1.10$	$82.15$
${\boldsymbol{P}}{\boldsymbol{T}}{\boldsymbol{A}}{\boldsymbol{A}}$	$0.50$	$1\times {10}^{21}$	$1\times {10}^{11}$	$20.24$	$22.79$	$1.09$	$81.50$
${\boldsymbol{M}}{\boldsymbol{o}}{{\boldsymbol{O}}}_{3}$	$0.50$	$1\times {10}^{21}$	$1\times {10}^{11}$	$20.57$	$22.79$	$1.10$	$82.27$
${\boldsymbol{M}}{\boldsymbol{o}}{{\boldsymbol{S}}}_{2}$	$0.50$	$1\times {10}^{21}$	$1\times {10}^{11}$	$20.55$	$22.79$	$1.10$	$82.25$
${{\boldsymbol{C}}{\boldsymbol{u}}}_{2}{\boldsymbol{T}}{\boldsymbol{e}}$	$0.50$	$1\times {10}^{21}$	$1\times {10}^{11}$	$20.43$	$22.75$	$1.09$	$82.11$
${\boldsymbol{n}}{\boldsymbol{P}}{\boldsymbol{B}}$	$0.50$	$1\times {10}^{21}$	$1\times {10}^{11}$	$20.59$	$22.83$	$1.10$	$82.25$
${{\boldsymbol{S}}{\boldsymbol{b}}}_{2}{{\boldsymbol{S}}}_{3}$	$0.50$	$1\times {10}^{21}$	$1\times {10}^{11}$	$21.83$	$24.26$	$1.10$	$81.87$

Changing the HTL influences the performance of PSC, as demonstrated by the results presented in Table 3. This variation in performance can be interpreted by many factors governing the transport and extraction of carriers, including the HTL alignment with the perovskite absorber, which can be expressed in terms of the valence band offset (VBO), which can be numerically determined as follows ^52^:

6 $$VBO={E}_{VB, Lower Absorber}-{E}_{VB,HTL}$$

here, ${E}_{VB, Lower Absorber}$ and ${E}_{VB, HTL}$ are the valence band (VB) edge energies of the Cs_2_AgBiI_6_ layer and the HTL, respectively. When the VB of the absorber is above that of the HTL, positive VBOs, or spikes, are introduced at the interface, which impede hole flow in the desired direction toward the HTL ^45^. Materials with a negative VBO, with HTL VB laying above that of the absorber, create energy cliffs. This generally facilitates hole transport into the HTL making such materials favorable. Nevertheless, an excessively large cliff may increase carrier recombination and reduce device performance ^53^. Accordingly, the utilization of PEDOT: PSS and CFTS as HTLs declines the performance of the suggested device as a consequence of their VBO values of $-0.5\ eV$ and $-0.9\ eV$, respectively, which are considered to be relatively high cliffs at the interface of the lower Cs_2_AgBiI_6_ absorber. It is noteworthy that the small positive VBO spikes of $0.08\ eV$ and $0.1\ eV$ at the lower absorber interface with CuO and Cu_2_O HTLs have minimal impact on performance, yielding PCE values of $20.58 \%$ and $20.51 \%$, respectively. On the other hand, employing HTLs with moderate VBO cliff values enhances the performance of the proposed device, as utilizing Sb_2_S_3_ ($-0.18\ eV$) and P3HT ($-0.3\ eV$) increases the PCE to $21.83 \%$ and $21.33 \%$, respectively. The results also reveal that Sb_2_S_3_ achieved the highest performance among all other utilized HTLs in this study.

**Fig. 3:**
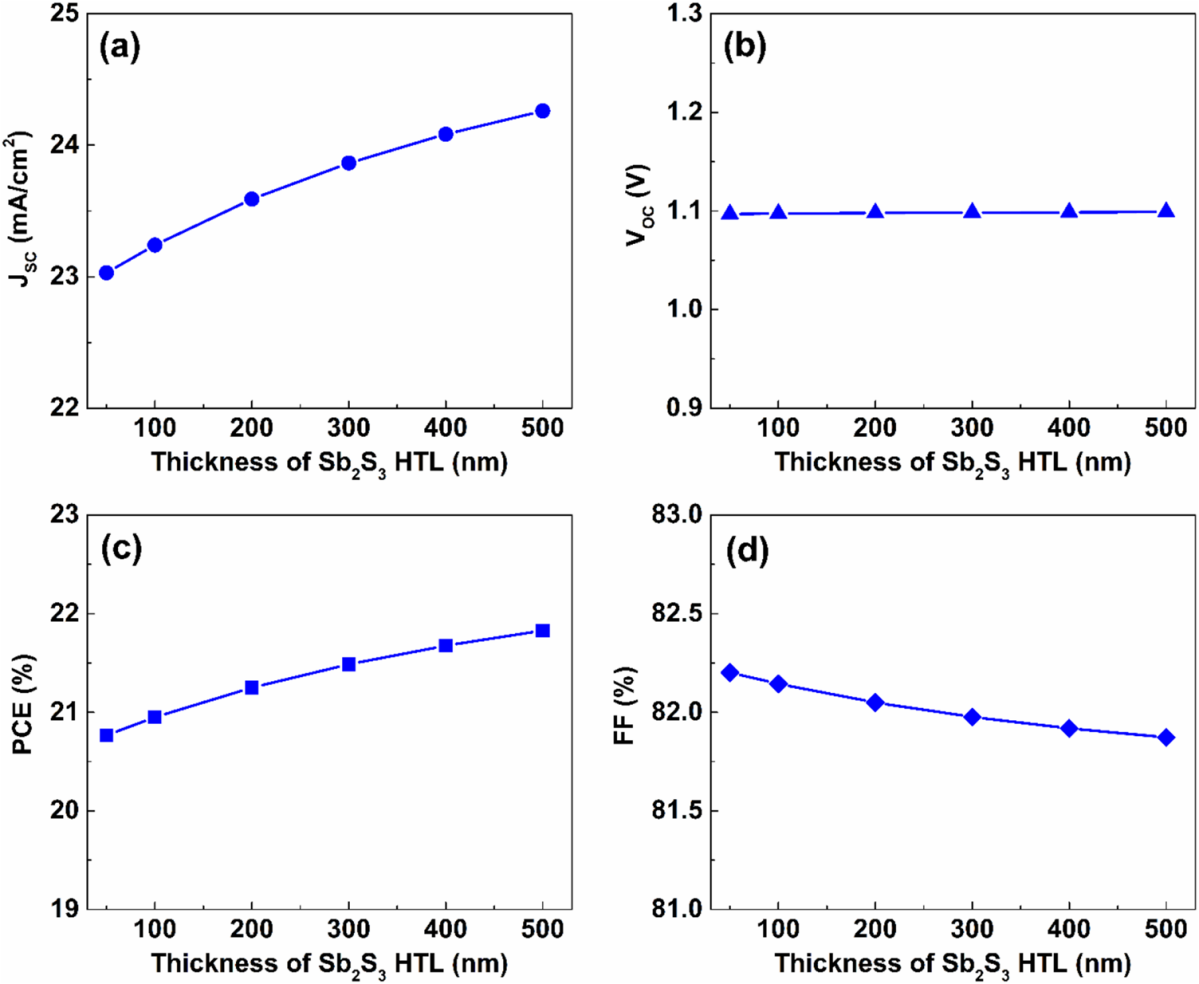
The effect of thickness variation of the Sb_2_S_3_ layer on the proposed dual-absorber PSC’s performance.

**Fig. 4:**
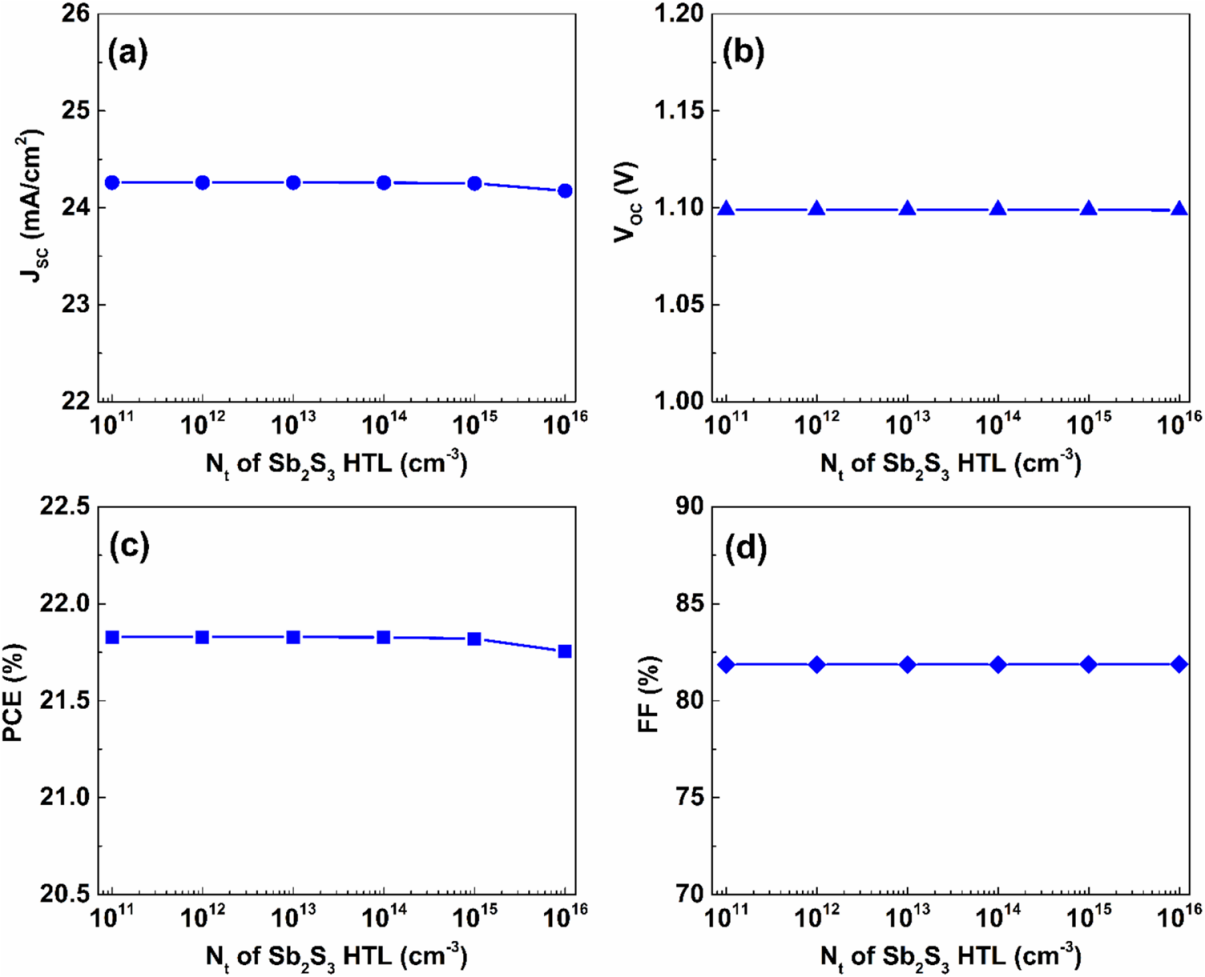
The effect of defect density variation of the Sb_2_S_3_ layer on the proposed dual-absorber PSC’s performance.

**Fig. 5:**
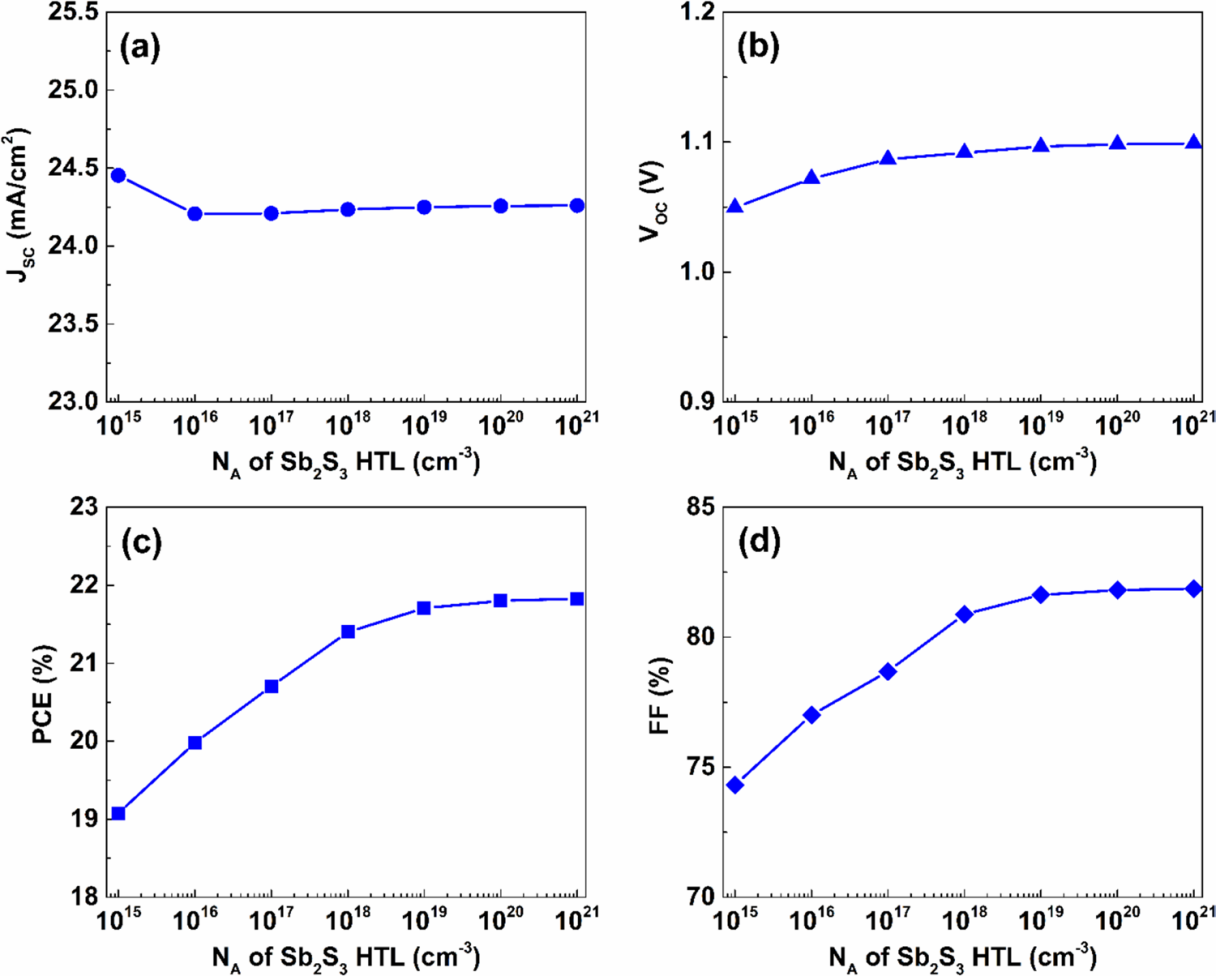
The effect of doping density variation of the Sb_2_S_3_ layer on the proposed device’s performance.

Figures 3–5 present the influence of changing the thickness, defect, and the acceptor doping densities of Sb_2_S_3_ HTL, respectively, on the proposed dual-absorber PSC performance. As illustrated in Fig. 3, increasing the Sb_2_S_3_ HTL thickness from $50$ to $500\ nm$ enhances performance. Specifically, J_SC_ rises by $5.34 \%$ from $23.03$ to $24.26\ mA/{cm}^{2}$ as thickness increases to $500\ nm$.

In contrast, the value of V_OC_ is almost unaffected, maintaining a constant value near $1.1 V$. The PCE of the proposed structure rises from $20.77$ to $21.83 \%$ as the HTL thickness elevates from $50$ to $500\ nm$, while the FF reduced by $0.33 \%$ over the same thickness range.

The defect density of the Sb_2_S_3_ HTL showed a slight impact on the performance of the proposed cell. As shown in Fig. 4, increasing the Sb_2_S_3_ layer’s defect density from $1\times {10}^{11}$ to $1\times {10}^{16} {\ cm}^{-3}$ induces a minor reduction in the effectiveness of the suggested PSC. In detail, the value of J_SC_ is kept constant at $24.26\ mA/{cm}^{2}$ while changing the defect concentration from $1\times {10}^{11}$ to $1\times {10}^{14} {\ cm}^{-3}$, then it slightly reduces to $24.25\ mA/{cm}^{2}$ and $24.18\ mA/{cm}^{2}$ with the increase in the bulk defect concentration to $1\times {10}^{15} {\ cm}^{-3}$ and $1\times {10}^{16} {\ cm}^{-3}$, respectively. Furthermore, the PCE value only reduces by $0.08 \%$ upon the increase of defect concentration to $1\times {10}^{16} {\ cm}^{-3}$. In addition, the values of V_OC_ and FF are almost unaffected by the Sb_2_S_3_ layer’s defect density variation over the employed range, keeping their values almost constant at $1.1\ V$ and $81.87 \%$, respectively.

The density of acceptor atoms in the Sb_2_S_3_ HTL, on the other hand, has a considerable impact on the performance of the proposed device. A strong influence on the strength of the electric field at the HTL/absorber interfaces is induced by the doping concentration within the HTL which makes it a critical parameter for device performance. The enhanced electric field promotes more effective separation of the generated electron- hole pairs, which increases the overall performance ^54^. As indicated by Fig. 5, increasing N_A_ of Sb_2_S_3_ from $1\times {10}^{15}$ to $1\times {10}^{16} {\ cm}^{-3}$ diminishes J_SC_ by $0.98 \%$, reducing its value from $24.25$ to $24.21\ mA/{cm}^{2}$, then it shows a slight increase to $24.26\ mA/{cm}^{2}$ upon rising the acceptor density to $1\times {10}^{21} {\ cm}^{-3}$. The value of V_OC_ rises by $4.55 \%$ from $1.05\ V$ at doping concentration of $1\times {10}^{15} {\ cm}^{-3}$ to $1.1\ V$ at N_A_ of $1\times {10}^{21} {\ cm}^{-3}$. Further, the values of PCE and FF increase with the Sb_2_S_3_ layer’s acceptor doping density increase, achieving the maximum PCE of $21.83 \%$ and FF of $81.87 \%$, at an Sb_2_S_3_ doping density of $1\times {10}^{21} {\ cm}^{-3}$.

### Impact of utilizing different ETLs on device performance

**Fig. 6:**
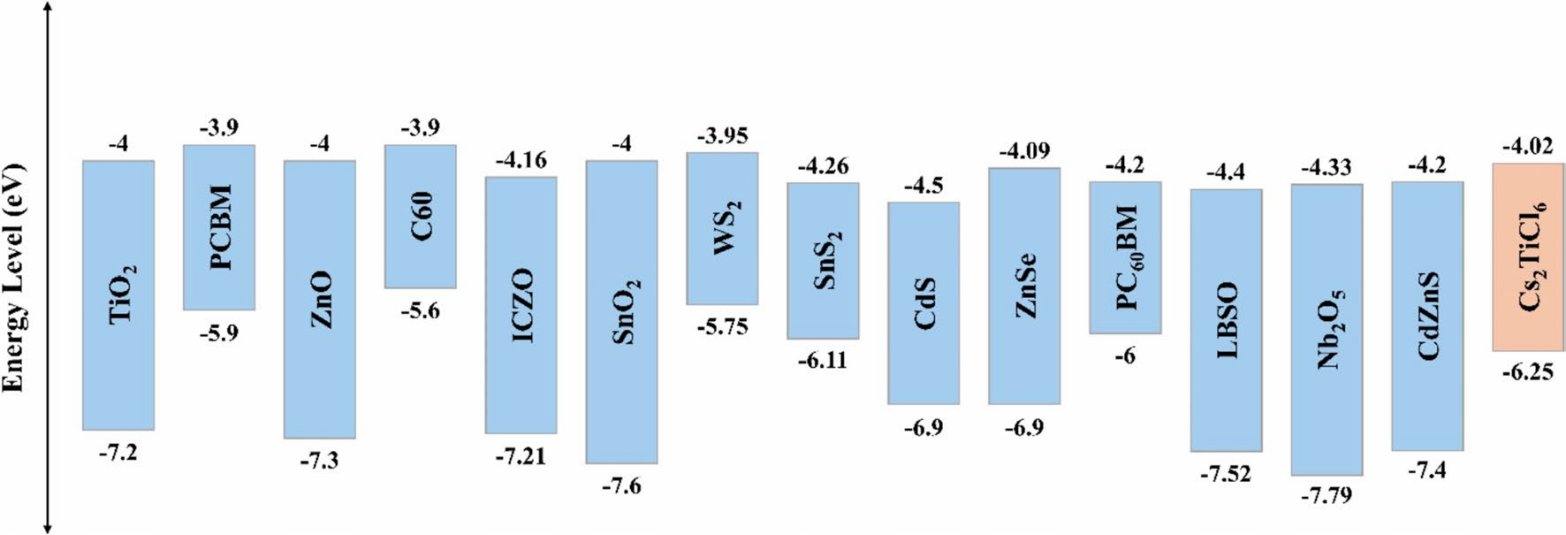
The alignment of energy bands of the different utilized ETLs with upper Cs_2_TiCl_6_ absorber.

The investigation was extended to identify the optimal ETL, using the initial device configuration shown in Fig. 1(a), while substituting the HTL with Sb_2_S_3_, which was shown in the preceding subsection to be the most effective HTL among the evaluated candidates. Several common electron transport materials were assessed including TiO_2_, PCBM, C_60_, IGZO, SnO_2_, WS_2_, SnS_2_, CdS, ZnSe, PC_60_BM, LBSO, Nb_2_O_5_, and CdZnS. The main electrical characteristics of the employed ETLs are provided in Table S2 of the Supplementary Information, while the corresponding energy band alignments with the upper Cs_2_TiCl_6_ absorber are indicated in Fig. 6.

To determine the optimum ETL configuration, which escalates device performance, another systematic batch analysis was performed. The investigation examined the influence of thickness, donor doping, and defect concentrations of each utilized material on the proposed device performance. Specifically, the ETL thickness was varied from $50$ to $500\ nm$, the donor doping concentration ranged between $1\times {10}^{15}$ and $1\times {10}^{20}{\ cm}^{-3}$, and the density of bulk defects ranged between $1\times {10}^{11}$ and $1\times {10}^{16}{\ cm}^{-3}$. Figures S4-S6 of the Supplementary Information present the impact of the examined parameters on the performance, whereas Table 4 reports the electrical characteristics associated with the maximum performance achieved when employing different ETLs with their optimized thickness, doping, and defect densities.

**Table 4:** Electrical parameters of introduced device corresponding to the maximum performance employing different ETLs.

ETL Material	${\boldsymbol{T}}{\boldsymbol{h}}{\boldsymbol{i}}{\boldsymbol{c}}{\boldsymbol{k}}{\boldsymbol{n}}{\boldsymbol{e}}{\boldsymbol{s}}{\boldsymbol{s}}$ $({\boldsymbol{\mu}}{\boldsymbol{m}})$	${{\boldsymbol{N}}}_{{\boldsymbol{D}}}$ $({{\boldsymbol{c}}{\boldsymbol{m}}}^{-3})$	${{\boldsymbol{N}}}_{{\boldsymbol{t}}}$ $({{\boldsymbol{c}}{\boldsymbol{m}}}^{-3})$	${\boldsymbol{P}}{\boldsymbol{C}}{\boldsymbol{E}}(\boldsymbol{\%})$	${{\boldsymbol{J}}}_{{\boldsymbol{S}}{\boldsymbol{C}}}$ $({\boldsymbol{m}}{\boldsymbol{A}}/{{\boldsymbol{c}}{\boldsymbol{m}}}^{2})$	${{\boldsymbol{V}}}_{{\boldsymbol{O}}{\boldsymbol{C}}}$ $({\boldsymbol{V}})$	${\boldsymbol{F}}{\boldsymbol{F}}$ $(\boldsymbol{\%})$
${\boldsymbol{Z}}{\boldsymbol{n}}{\boldsymbol{O}}$	$0.05$	$1\times {10}^{19}$	$1\times {10}^{11}$	$21.84$	$24.26$	$1.10$	$81.92$
${{\boldsymbol{T}}{\boldsymbol{i}}{\boldsymbol{O}}}_{2}$	$0.10$	$1\times {10}^{19}$	$1\times {10}^{11}$	$21.84$	$24.26$	$1.10$	$81.92$
${\boldsymbol{P}}{\boldsymbol{C}}{\boldsymbol{B}}{\boldsymbol{M}}$	$0.05$	$1\times {10}^{20}$	$1\times {10}^{11}$	$19.18$	$21.47$	$1.09$	$81.68$
${\boldsymbol{C}}60$	$0.05$	$1\times {10}^{20}$	$1\times {10}^{11}$	$17.60$	$19.73$	$1.09$	$81.83$
${\boldsymbol{I}}{\boldsymbol{G}}{\boldsymbol{Z}}{\boldsymbol{O}}$	$0.05$	$1\times {10}^{20}$	$1\times {10}^{11}$	$21.82$	$24.26$	$1.10$	$81.83$
${{\boldsymbol{S}}{\boldsymbol{n}}{\boldsymbol{O}}}_{2}$	$0.05$	$1\times {10}^{20}$	$1\times {10}^{11}$	$21.82$	$24.23$	$1.10$	$81.94$
${{\boldsymbol{W}}{\boldsymbol{S}}}_{2}$	$0.05$	$1\times {10}^{20}$	$1\times {10}^{11}$	$18.37$	$20.54$	$1.09$	$81.87$
${{\boldsymbol{S}}{\boldsymbol{n}}{\boldsymbol{S}}}_{2}$	$0.50$	$1\times {10}^{20}$	$1\times {10}^{11}$	$22.15$	$24.50$	$1.10$	$82.03$
${\boldsymbol{C}}{\boldsymbol{d}}{\boldsymbol{S}}$	$0.50$	$1\times {10}^{20}$	$1\times {10}^{11}$	$19.90$	$24.24$	$1.14$	$71.80$
${\boldsymbol{Z}}{\boldsymbol{n}}{\boldsymbol{S}}{\boldsymbol{e}}$	$0.50$	$1\times {10}^{20}$	$1\times {10}^{11}$	$21.84$	$24.26$	$1.10$	$81.95$
${{\boldsymbol{P}}{\boldsymbol{C}}}_{60}{\boldsymbol{B}}{\boldsymbol{M}}$	$0.05$	$1\times {10}^{20}$	$1\times {10}^{11}$	$17.32$	$20.90$	$1.10$	$75.66$
${\boldsymbol{L}}{\boldsymbol{B}}{\boldsymbol{S}}{\boldsymbol{O}}$	$0.50$	$1\times {10}^{20}$	$1\times {10}^{11}$	$19.26$	$24.22$	$1.12$	$71.24$
${{\boldsymbol{N}}{\boldsymbol{b}}}_{2}{{\boldsymbol{O}}}_{5}$	$0.05$	$1\times {10}^{20}$	$1\times {10}^{11}$	$21.34$	$24.21$	$1.10$	$79.85$
${\boldsymbol{C}}{\boldsymbol{d}}{\boldsymbol{Z}}{\boldsymbol{n}}{\boldsymbol{S}}$	$0.05$	$1\times {10}^{20}$	$1\times {10}^{11}$	$21.81$	$24.26$	$1.10$	$81.81$

Changing the ETL clearly affects the performance of PSC, as indicated by the results presented in Table 4. This variation in performance can be interpreted by many factors governing the transport and extraction of carriers including the amount of absorbed light within the ETL and its absorption coefficient in addition to the alignment of the ETL with the upper absorber layer which can be expressed in terms of the conduction band offset (CBO) which can be determined at the interface of the ETL as follows ^52^:

7 $$CBO={E}_{CB,ETL}-{E}_{CB, Upper Absorber}$$

where, ${E}_{CB,Upper Absorber}$ and ${E}_{CB, ETL}$ denote the edge energies of conduction bands (CBs) of the Cs_2_TiCl_6_ layer and the ETL, respectively. Positive CBOs or spikes are created when the CB of the ETL lies above the absorber’s CB, leading to resistance of the electron flow in the desired direction into ETL ^45^.

On the other hand, materials with negative CBO with CB of the ETL laying below that of absorber create energy cliffs with absorber layer, which generally facilitates electron transport into ETL making such materials favorable. Nevertheless, excessively large cliffs increase carrier recombination which leads to device performance deterioration ^53^. Therefore, employing ETLs that create positive CBO spikes at the interface with the upper Cs_2_TiCl_6_ absorber, such as C60 ($0.1\ eV$) and WS_2_ ($0.05\ eV)$ reduces the device PCE to $17.6 \%$ and $18.37 \%$, respectively. Further, utilizing materials that create significant energy cliffs at the interface with upper absorber negatively affects the device effectiveness, resulting in PCE values of $19.9 \%$ for CdS ($-0.5\ eV$) and $19.26 \%$ for LBSO ($-0.4\ eV$). In contrast, using ETLs with moderate CBO cliff values escalates the performance of the introduced PSC as shown by the escalated PCE when SnS_2_ ($-0.26\ eV$) and ZnSe ($-0.09\ eV$) were employed. The results also indicates that SnS_2_ achieves the highest performance among the investigated ETLs in this study.

**Fig. 7:**
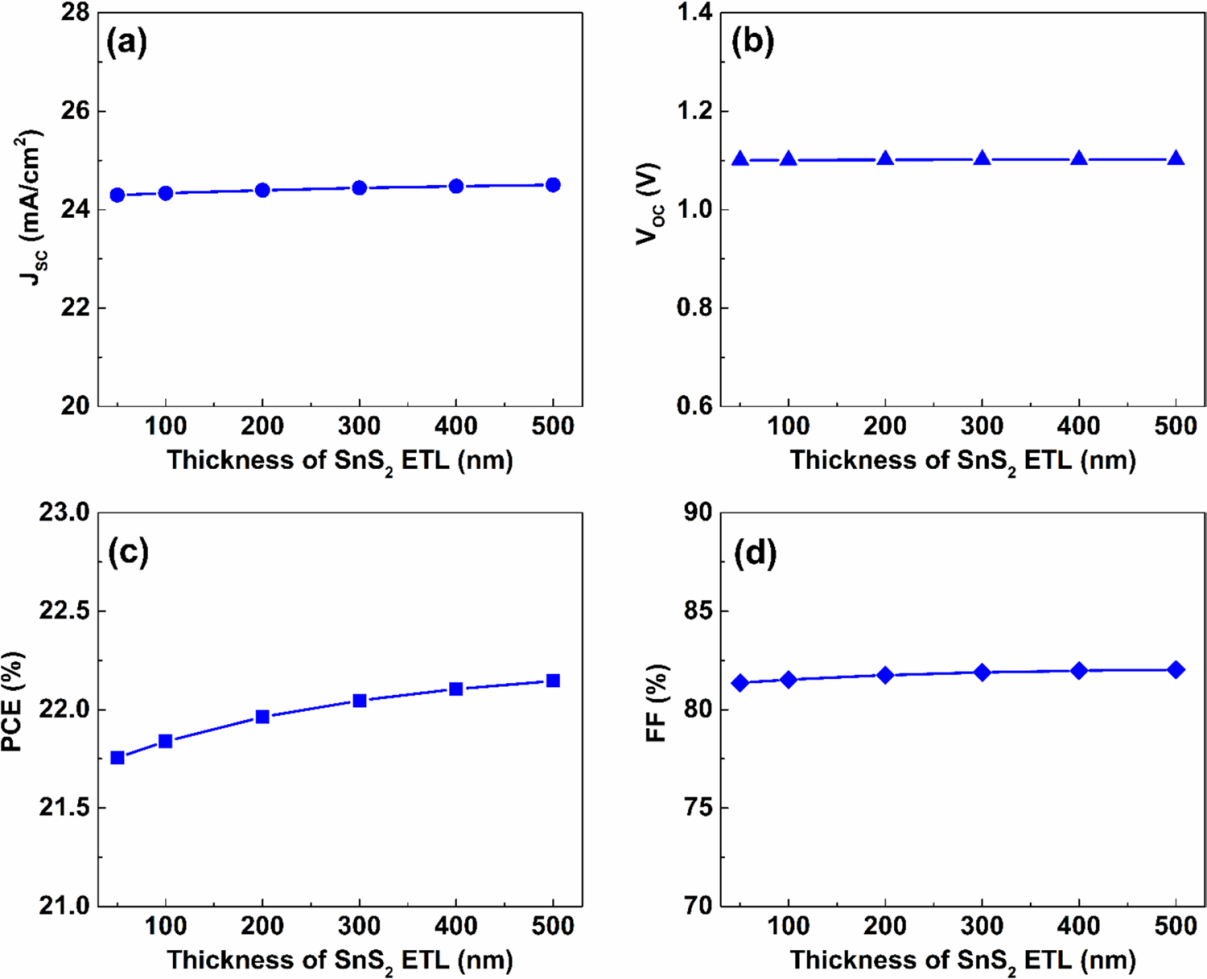
The effect of thickness variation of the SnS_2_ layer on the proposed dual-absorber PSC’s performance.

**Fig. 8:**
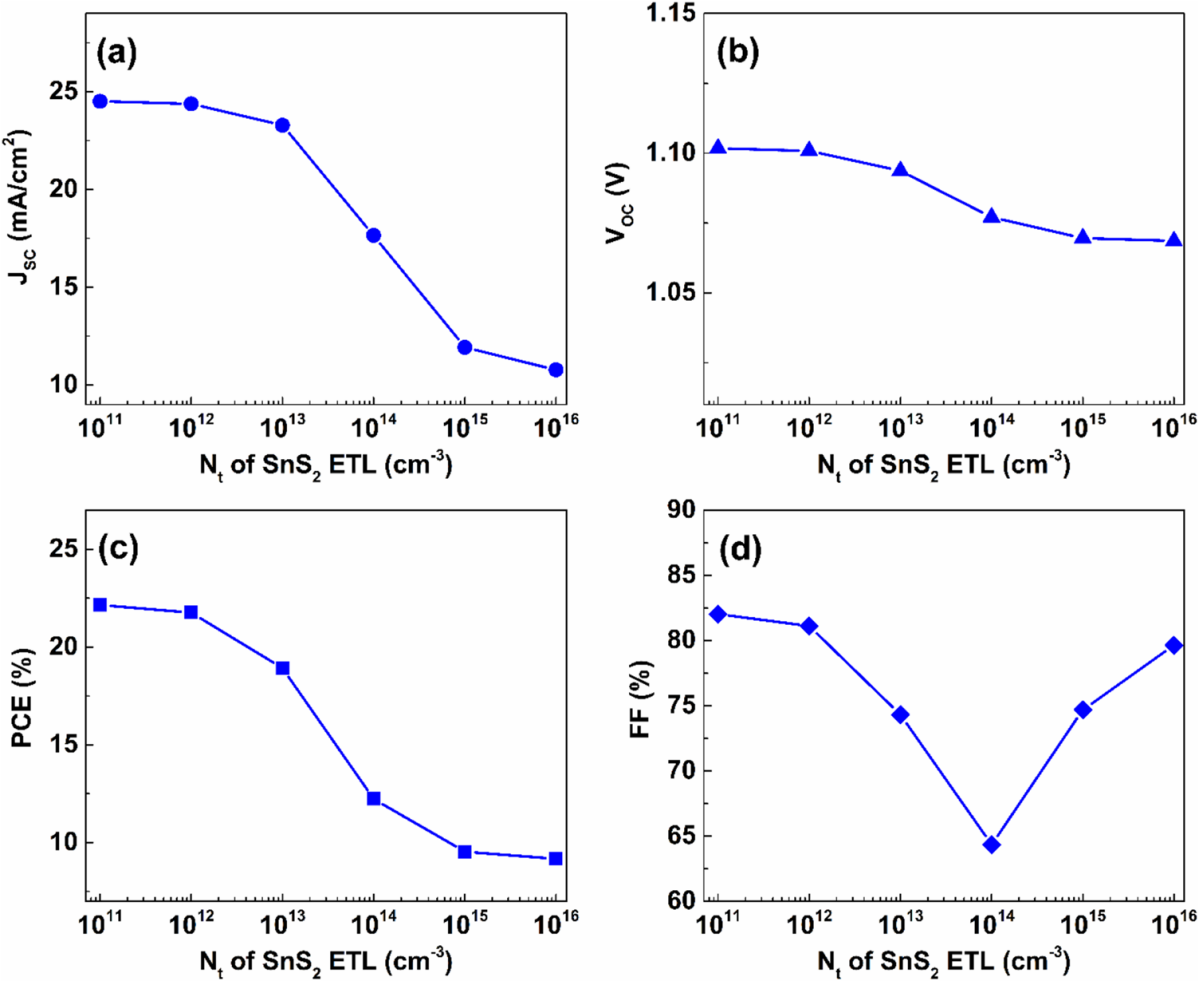
The effect of defect density variation of the SnS_2_ layer on the proposed dual-absorber device’s performance.

**Fig. 9:**
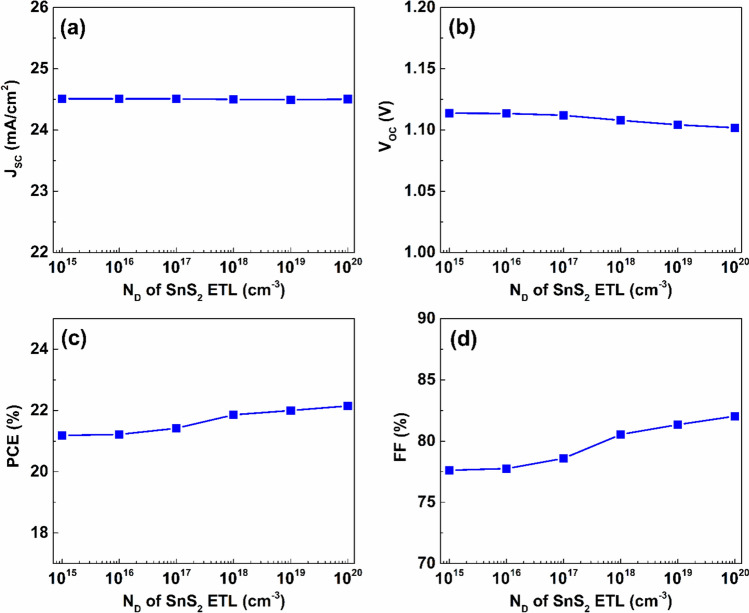
The effect of doping density variation of the SnS_2_ layer on the proposed dual-absorber device’s performance.

Figures 7–9 illustrate the influence of varying thickness, defect density, and the donor doping density of SnS_2_ ETL on the effectiveness of the introduced Cs_2_TiCl_6_- Cs_2_AgBiI_6_ half tandem device. As presented in Fig. 7, rising the thickness of the SnS_2_ ETL from $50$ to $500\ nm$ slightly escalates performance of the Cs_2_TiCl_6_-Cs_2_AgBiI_6_ proposed PSC. Particularly, J_SC_ increases by $0.82\%$ from $24.3$ to $24.5\ mA/{cm}^{2}$ while elevating the thickness to $500\ nm$. In addition, V_OC_ exhibits negligible variation, remaining essentially constant around $1.1\ V$. Further, PCE of the proposed PSC rises from $21.76$ to $22.15 \%$ as the ETL thickness increases from $50$ to $500\ nm$, while the value of FF increases from $81.36$ to $82.03 \%$ over the same thickness range.

On the other hand, the bulk defect concentration of the SnS_2_ ETL has a more significant effect on the performance of Cs_2_TiCl_6_-Cs_2_AgBiI_6_ proposed device. Defects in ETL, which can be originated from structural dislocations, native imperfection, or contamination by foreign atoms, contribute to the creation of both shallow and deep traps. As indicated in Fig. 8, increasing concentration of defects of the SnS_2_ ETL from $1\times {10}^{11}$ to $1\times {10}^{16} {\ cm}^{-3}$ causes a tremendous reduction of the proposed PSC performance. In detail, J_SC_ reduces from $24.5$ to $10.78\ mA/{cm}^{2}$ with the increase of the defect density of the SnS_2_ layer from $1\times {10}^{11}$ to $1\times {10}^{16} {\ cm}^{-3}$. Furthermore, V_OC_ decreases by $2.73 \%$ from $1.1$ to $1.07\ V$ due to escalating N_t_ from $1\times {10}^{11}$ to $1\times {10}^{16} {\ cm}^{-3}$. In addition, the value of PCE faces a massive reduction of $12.97 \%$ upon the increase of defect density to $1\times {10}^{16} {\ cm}^{-3}$. Also, the value of FF was influenced by the variation of SnS_2_ layer’s defect concentration over the employed range. Increasing N_t_ of the SnS_2_ layer from $1\times {10}^{11}$ to $1\times {10}^{14} {\ cm}^{-3}$ results in $7.72 \%$ reduction of FF. Beyond that, FF starts to rise in correspondence with the increase of defect density.

Additionally, the donor doping density of SnS_2_ layer has considerable influence on performance. As indicated by Fig. 9, increasing N_D_ of SnS_2_ layer from $1\times {10}^{15}$ to $1\times {10}^{20} {\ cm}^{-3}$ has a negligible impact on J_SC_, as it remains essentially constant around $24.5\ mA/{cm}^{2}$. The value of V_OC_ slightly reduced by $0.01\ V$ from $1.11\ V$ at doping density of $1\times {10}^{15} {\ cm}^{-3}$ to $1.1\ V$ at $1\times {10}^{20} {\ cm}^{-3}$. Further, values of PCE and FF were escalated as result of increasing SnS_2_ doping density, achieving maximum PCE and FF values of $22.15 \%$ and $82.03 \%$, respectively, with SnS_2_ layer’s doping density of $1\times {10}^{20} {\ cm}^{-3}$.

### Structural optimization of proposed dual-absorbers

In the following subsections, the effects of variations in the thickness, bulk defect, and doping densities of photo-absorber layers on the performance of the Cs_2_TiCl_6_-Cs_2_AgBiI_6_ bi-absorber proposed PSC are analyzed. The device is configured with Sb_2_S_3_ and SnS_2_ as HTL and ETL, respectively, based on their superior effectiveness among the examined CTLs. The aim of this part of the study is to optimize the device performance and maximize the PCE of the proposed device.

#### Effect of thickness variation of upper absorber on performance

**Fig. 10:**
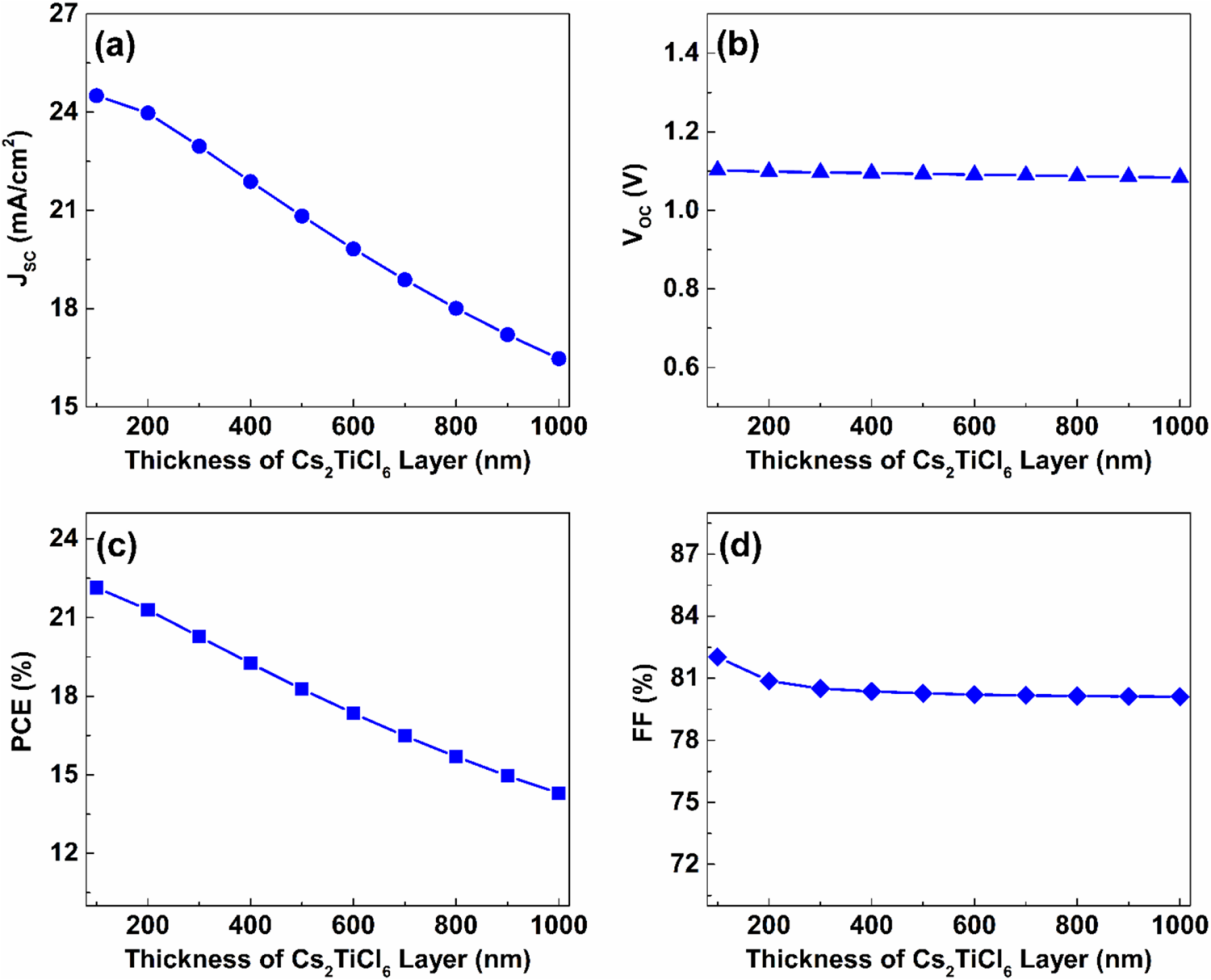
The impact of thickness variation of the Cs_2_TiCl_6_ upper absorber on the proposed device’s performance.

The absorber layer’s thickness can be regarded as one of the critical factors which determine the effectiveness of different types of solar cells. The optimum value of the absorber thickness is primally impacted by the absorber’s absorption coefficient and its bandgap. While increasing the absorber thickness may escalate light absorption, it also may result in elevated rates of carrier recombination and in turn diminish performance. In contrast, a thinner absorber layer can minimize recombination losses but reduces light absorption ^55^. The impact of thickness variation of the upper absorber on the effectiveness of the device was evaluated through an analysis in which the thickness of Cs_2_TiCl_6_ film was systematically varied from $100$ to $1000\ nm$ with an iteration step of $100\ nm$, while Cs_2_AgBiI_6_ layer was kept with fixed thickness of $700\ nm$. As indicated in Fig. 10(a), increasing the thickness of the Cs_2_TiCl_6_ layer from $100$ to $1000\ nm$ causes a $32.78 \%$ reduction of J_SC_ from $24.5$ to $16.47\ mA/{cm}^{2}$. In addition, changing the Cs_2_TiCl_6_ layer thickness has a minimal impact on the V_OC_ value, decreasing from $1.1$ to $1.08\ V$ over the same thickness range as shown in Fig. 10(b). The PCE value also declined from $22.15$ to $14.3 \%$ with the increase in Cs_2_TiCl_6_ thickness from $100\ nm$ to $1000\ nm$, while FF decreased from $82.03$ to $80.11 \%$ over the same thickness range, as shown in Figs. 10(c) and (d).

The decline in device performance caused by increasing the thickness of the upper absorber can be attributed to the reduction of light transmission to the lower Cs_2_AgBiI_6_ absorber, which has a higher absorption coefficient. As a result, fewer charge carriers are generated, which results in a decrease in JSC and overall cell performance.

#### Effect of defect density variation of upper absorber on PSC performance

The density of defects within the main absorber layers critically affects the PSCs’ performance. Increasing defect densities can lead to absorber-quality deterioration, structural stress, and accelerated device degradation. Furthermore, these defects introduce trap states which facilitate non-radiative recombination and, in turn, reduce lifetime of carriers and overall device performance ^56^. Shockley–Read–Hall recombination can be used to characterize the impact of defect density on the photovoltaic performance by relating it to the defect concentration and the lifetime of charge carriers, as follows ^46^:

8 $${R}_{SRH}={R}_{SRH}=\frac{np-{{n}_{i}}^{2}}{{\tau}_{p}\left(n+{n}_{1}\right)+{\tau}_{n}\left(p+{p}_{1}\right)}$$

here, the lifetime of electrons and holes are represented by ${\tau}_{n}$ and ${\tau}_{p}$, respectively, and ${n}_{1}$ and ${p}_{1}$ can be calculated as follows ^57^:

9 $${n}_{1}={N}_{C} {e}^{\frac{-({E}_{C}-{E}_{t})}{K T}}$$

10 $${p}_{1}={N}_{v} {e}^{\frac{-({E}_{t}-{E}_{V})}{K T}}$$

where ${\mathrm{E}}_{t}$ is the trap level. To consider the effect of the bulk defect concentration within the Cs_2_TiCl_6_ upper absorber on the performance of the proposed cell, simulations were conducted by ramping the defect density from $1\times {10}^{11}$ to $1\times {10}^{16} {\ cm}^{-3}$, while the absorber layers’ thicknesses were held constant at $100\ nm$ and $700\ nm$ for the upper Cs_2_TiCl_6_ layer and the lower Cs_2_AgBiI_6_ layer, respectively.

**Fig. 11:**
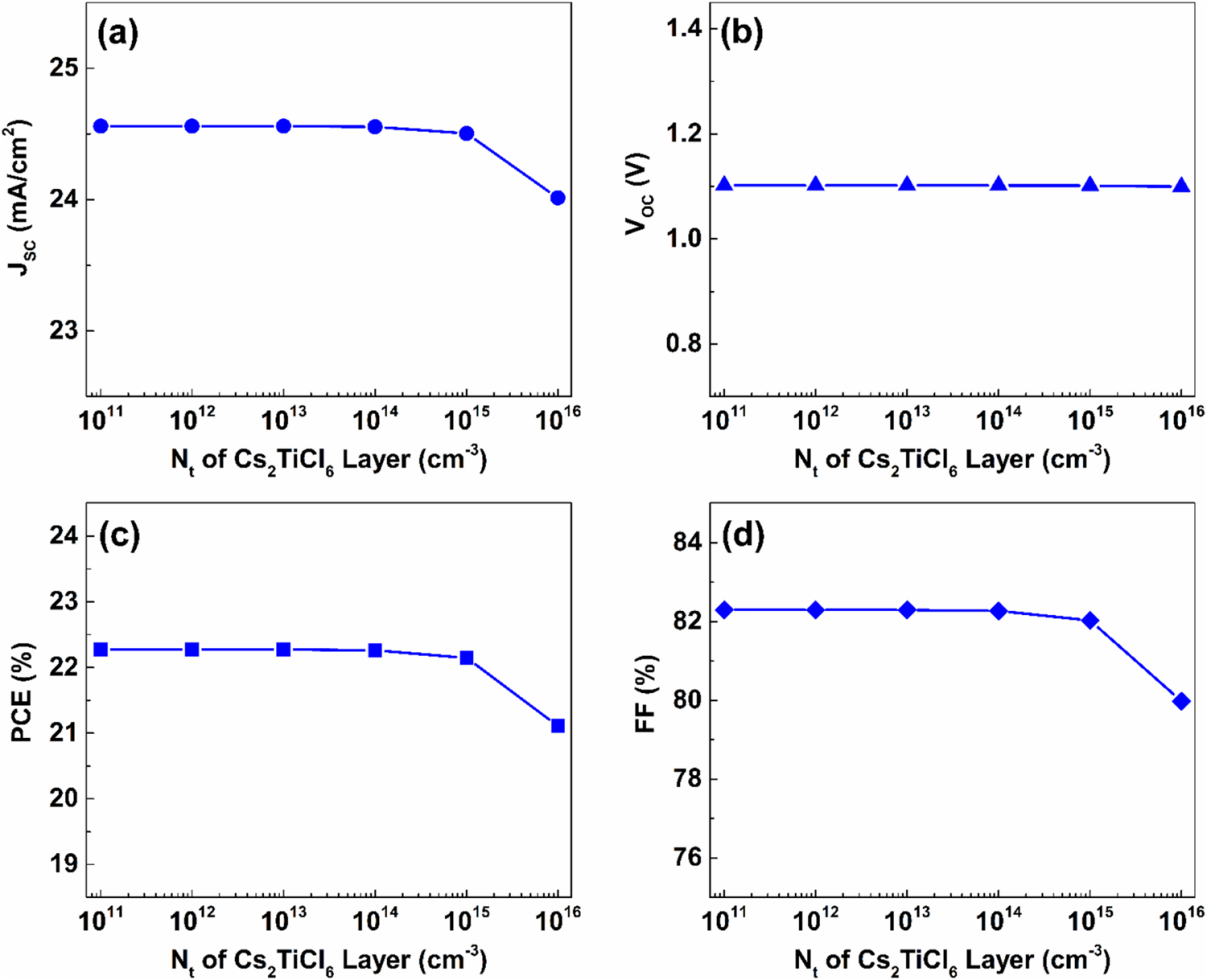
The effect of defect density variation of the Cs_2_TiCl_6_ upper absorber on the proposed dual-absorber device’s performance.

Results shown in Fig. 11 reveal that the proposed cell’s performance behaves as predicted, showing a clear dependence on the density of bulk defects within the absorber layer. Figure 11(a) indicates that increasing the Cs_2_TiCl_6_ upper layer’s defect density from $1\times {10}^{11}$ to $1\times {10}^{15} {\ cm}^{-3}$ has a negligible impact on J_SC_ value, with only a minor variation of $0.24\%$. However, when the concentration of defects exceeds $1\times {10}^{15} {\ cm}^{-3}$, J_SC_ begins to decline at a higher rate of approximately $0.5\ mA/{cm}^{2}$ per decade as the defect density continues to rise. Nevertheless, changing the defect density of the Cs_2_TiCl_6_ upper absorber does not considerably affect the open-circuit voltage. As shown in Fig. 11(b), increasing the upper absorber defect density from $1\times {10}^{11}$ to $1\times {10}^{16} {\ cm}^{-3}$ causes a $0.01\ V$ reduction in the value of V_OC_ from $1.1\ V$ at a defect density of $1\times {10}^{11} {\ cm}^{-3}$ to $1.09\ V$ at $1\times {10}^{16} {\ cm}^{-3}$. Figures 11(c) and (d) demonstrate that variations in Cs_2_TiCl_6_ defect density significantly affect PCE and FF, leading to a progressive decline in performance as the upper absorber defect density increases beyond $1\times {10}^{14} {\ cm}^{-3}$. The highest performance, characterized by a PCE of $22.27\%$ and an FF of $82.3\%$, was achieved when the upper absorber defect density was maintained below $1\times {10}^{14} {\ cm}^{-3}$.

#### Effect of doping density variation of upper absorber on performance

**Fig. 12:**
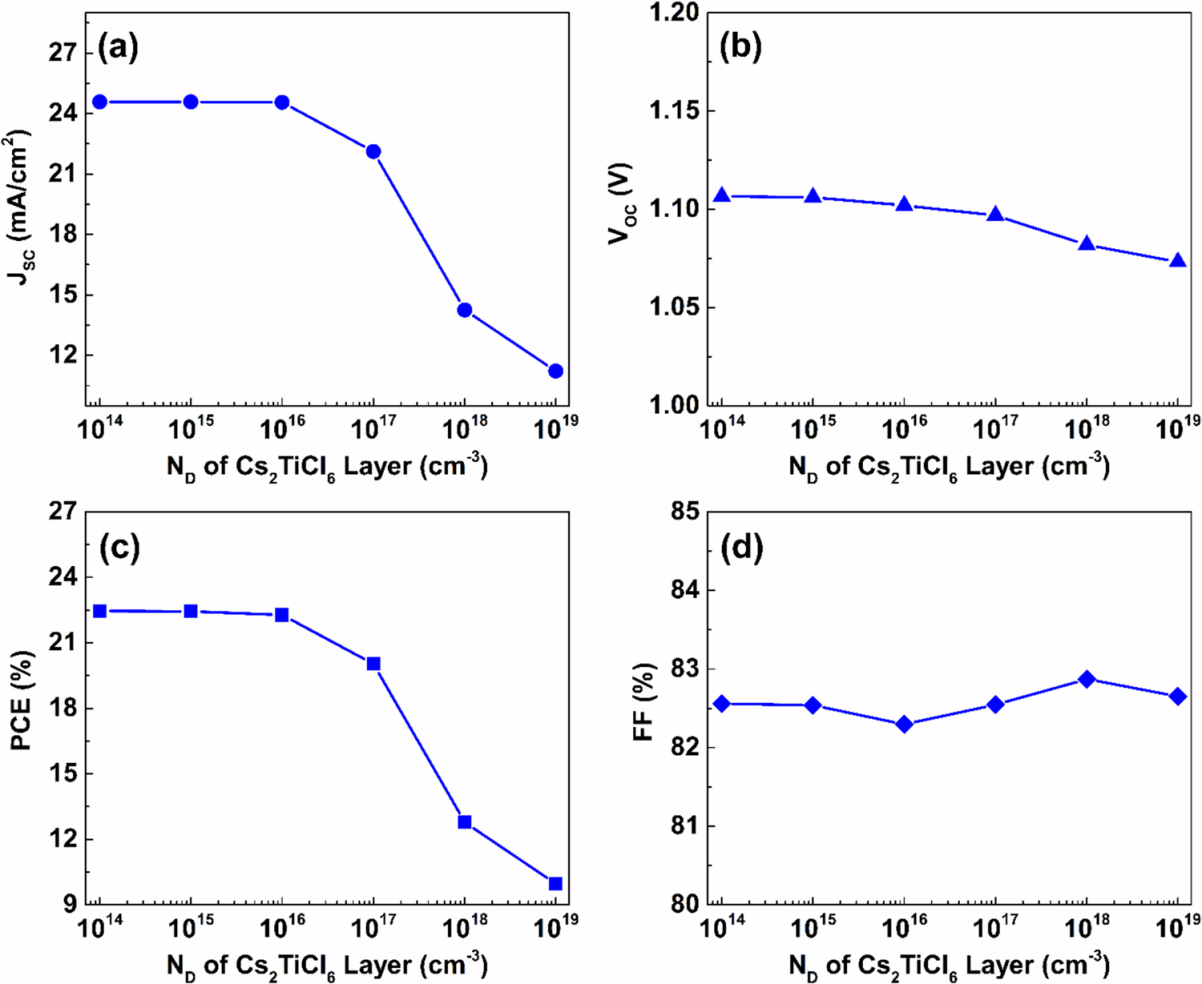
The effect of donor doping density variation of the Cs_2_TiCl_6_ upper absorber the proposed device’s performance.

The doping densities of the absorber layers have a crucial influence on the performance of PSCs. In this part of the study, a numerical simulation was conducted to investigate the impact of changing the donor density of the Cs_2_TiCl_6_ absorber on the key performance parameters. The outcomes resulting from the doping density variation of the upper absorber layer between $1\times {10}^{14} {\ cm}^{-3}$ and $1\times {10}^{19} {\ cm}^{-3}$ are presented in Figs. 12. As indicated in this figure, elevating the donor doping concentration within the upper absorber layer from $1\times {10}^{14} {\ cm}^{-3}$ to $1\times {10}^{15} {\ cm}^{-3}$ has almost no influence on the performance of the introduced device, with J_SC_, V_OC_, PCE, and FF levels remaining constant at $24.58\ mA/{cm}^{2}$, $1.11\ V$, $22.45\%$, and $82.55\%$, respectively. However, when the concentration of donor atoms exceeds $1\times {10}^{15} {\ cm}^{-3}$, J_SC_ begins to decrease significantly at an approximate average rate of $3.32\ mA/{cm}^{2}$ per decade as the donor density continues to rise to $1\times {10}^{19} {\ cm}^{-3}$.

Moreover, increasing the doping density of the Cs_2_TiCl_6_ upper absorber from $1\times {10}^{14} {\ cm}^{-3}$ to $1\times {10}^{19} {\ cm}^{-3}$ causes a slight reduction in V_OC_ from $1.11\ V$ to $1.07\ V$, as presented in Fig. 12(b). Further, Fig. 12(c) indicates that increasing the doping density of the Cs_2_TiCl_6_ upper absorber beyond $1\times {10}^{16} {\ cm}^{-3}$ significantly affects the PCE, leading to a deterioration in performance as the upper absorber doping density increases. The PCE value drops from $22.27\%$ at an N_D_ of $1\times {10}^{16} {\ cm}^{-3}$ to $9.96\%$ at an N_D_ of $1\times {10}^{19} {\ cm}^{-3}$. In contrast, the FF increases from $82.3\%$ to $82.65\%$ over the same doping density range, as shown in Fig. 12(d). This behavior can be attributed to the rise in donor density, which improves the electrical conductivity of the absorber layer, thereby improving the FF. However, excessive donor doping levels produce additional defect states within the material, resulting in elevated rates of carrier recombination and, consequently, a deterioration in overall device performance ^58^.

#### Impact of variation of lower absorber thickness on device performance

**Fig. 13:**
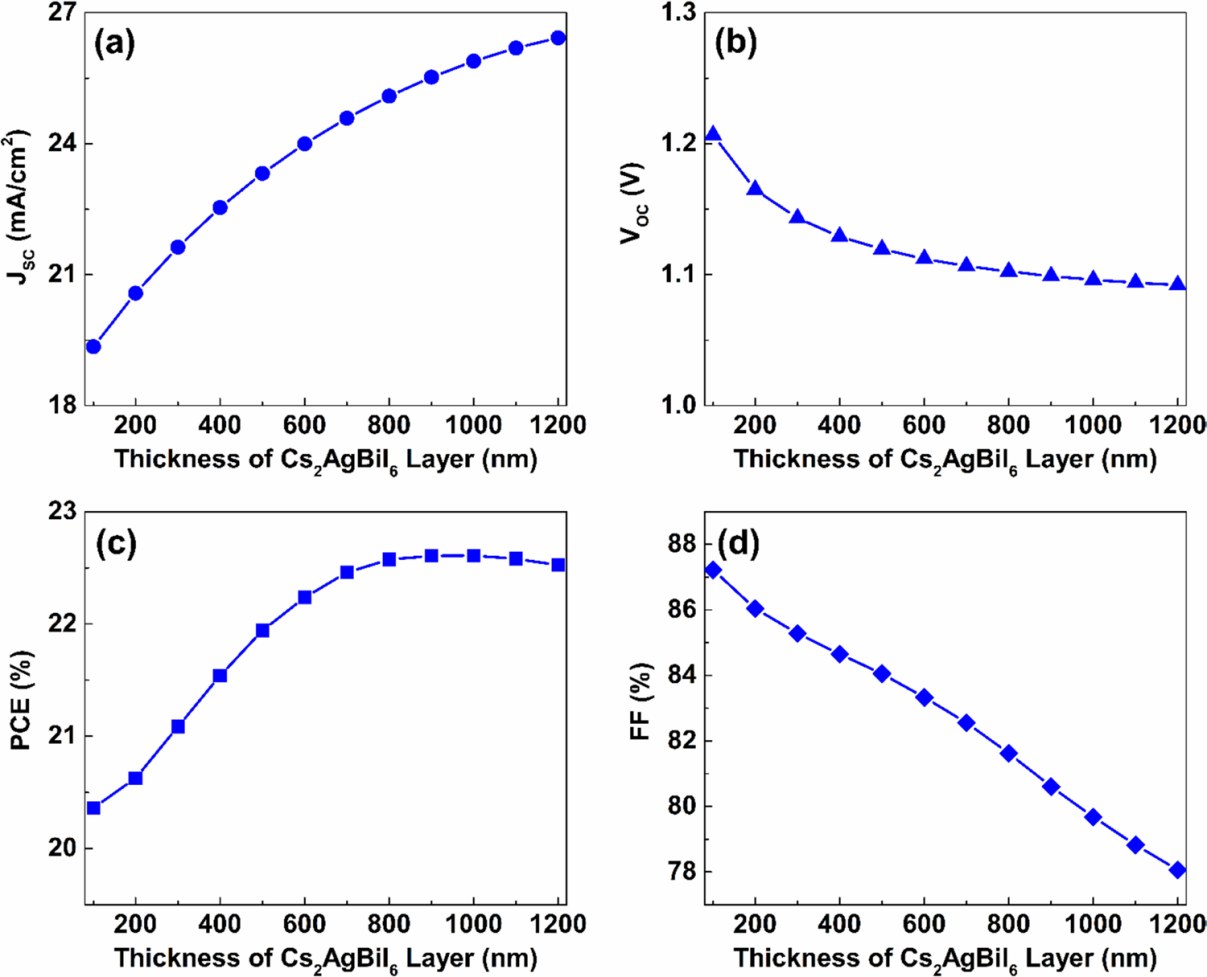
The effect of thickness variation of the Cs_2_AgBiI_6_ lower absorber the introduced dual-absorber device’s performance.

The influence of varying the thickness of the lower Cs_2_AgBiI_6_ absorber on the performance of the proposed PSC was investigated by conducting simulations with the Cs_2_AgBiI_6_ layer thickness being varied from $100\ nm$ to $1200\ nm$, using a $100\ nm$ interval step, while the thickness of the Cs_2_TiCl_6_ layer was maintained at $100\ nm$, as it was proven to be the optimal thickness for this layer in the preceding subsection. As indicated in Fig. 13(a), elevating the thickness of the Cs_2_AgBiI_6_ lower absorber from $100\ nm$ to $1200\ nm$ causes a $36.54\%$ enhancement in the J_SC_ value, from $19.35\ mA/{cm}^{2}$ to $26.42\ mA/{cm}^{2}$, as a thicker absorber layer promotes higher light absorption and, consequently, a greater generation of carriers. In contrast, varying the Cs_2_AgBiI_6_ layer thickness has a slight influence on V_OC_, causing it to reduce from $1.21\ V$ to $1.09\ V$ over the same thickness range, as shown in Fig. 13(b). The PCE value also increases from $20.36\%$ to $22.61\%$ with increasing bottom absorber thickness from $100\ nm$ to $1000\ nm$ as a result of the enhanced J_SC_.

Nevertheless, escalating the lower absorber thickness above $1000\ nm$ leads to increased carrier recombination within the absorber layer, which causes a minor reduction in PCE to $22.52\%$ with a further increase in layer thickness to $1200 nm$, as shown in Fig. 13(c). Moreover, the FF significantly reduced from $87.22\mathrm{\%}$ to $78.06\mathrm{\%}$ as the Cs_2_AgBiI_6_ layer thickness increases from $100\ nm$ to $1200\ nm$, as shown in Fig. 13(d).

#### Effect of defect density variation of lower absorber on performance

**Fig. 14:**
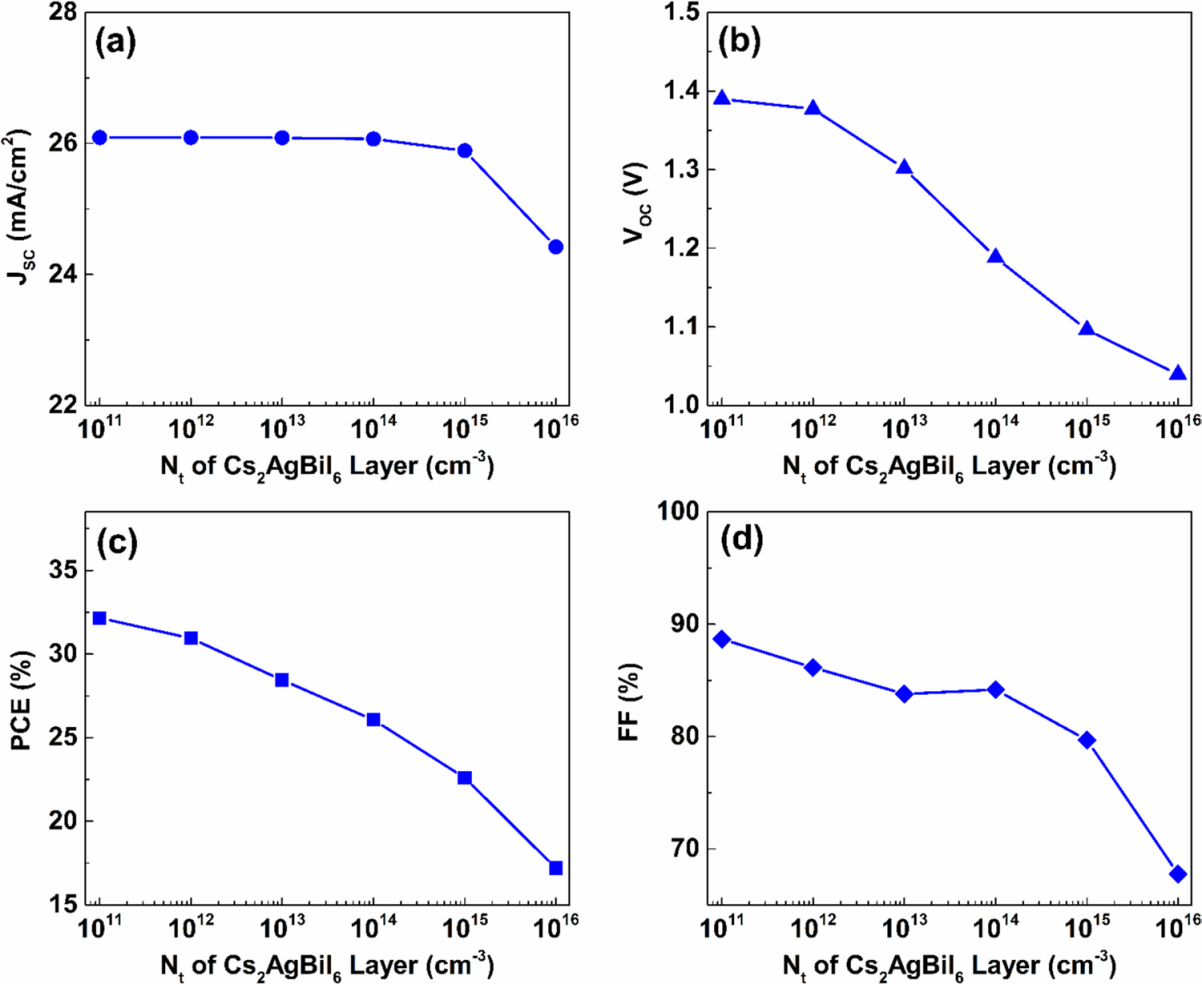
The effect of defect density variation of the Cs_2_AgBiI_6_ lower absorber the proposed dual-absorber device’s performance.

Simulations were conducted by varying the defect density between $1\times {10}^{10}{\ cm}^{-3}$ and $1\times {10}^{19} {\ cm}^{-3}$ to examine the impact of the defect density variation of the Cs_2_AgBiI_6_ layer on the performance of the proposed device, while the defect density and thickness of the Cs_2_TiCl_6_ layer defect were kept fixed at $1\times {10}^{10} {\ cm}^{-3}$ and $100\ nm$, respectively. The influence of the Cs_2_AgBiI_6_ layer’s defect density on the performance is indicated in Fig. 14. Increasing the defect density of the Cs_2_AgBiI_6_ lower absorber from $1\times {10}^{11} {\ cm}^{-3}$ to $1\times {10}^{13} {\ cm}^{-3}$ does not impact the J_SC_ value, as presented in Fig. 14(a).

Nevertheless, when the density of bulk defects surpasses $1\times {10}^{13} {\ cm}^{-3}$, J_SC_ begins to decline at an average rate of approximately $0.56\ mA/{cm}^{2}$ per decade as the density of bulk defects continues to rise. Furthermore, increasing the density of defects within the Cs_2_AgBiI_6_ lower absorber from $1\times {10}^{11} {\ cm}^{-3}$ to $1\times {10}^{16} {\ cm}^{-3}$ causes a significant reduction in V_OC_, from $1.39\ V$ to $1.04\ V$, as indicated in Fig. 14(b). Additionally, Figs. 14(c) and (d) reveal that variations in the density of defects in the Cs_2_AgBiI_6_ absorber considerably affect both the PCE and FF, leading to performance degradation as the bulk defect density of the lower absorber increases. The highest performance, which is characterized by a PCE of $32.15\%$ and an FF of $88.67\%$, was achieved when the defect concentration within the bottom absorber layer was maintained at a low level of $1\times {10}^{11} {\ cm}^{-3}$.

#### Effect of doping density variation of lower absorber on PSC performance

**Fig. 15:**
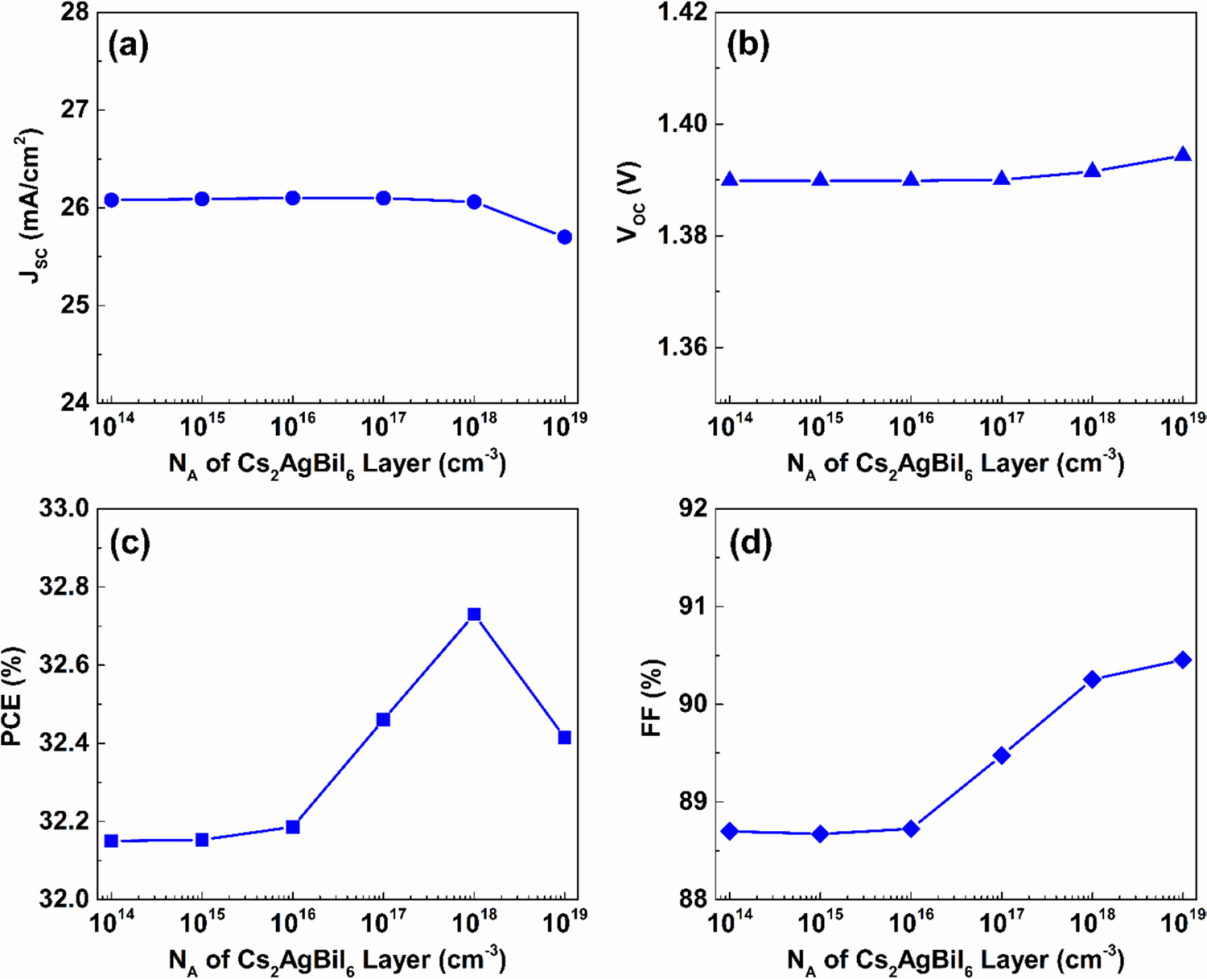
The impact of acceptor doping density variation of the Cs_2_AgBiI_6_ lower absorber the proposed dual-absorber device’s performance.

Increasing the acceptor atoms concentration within the bottom Cs_2_AgBiI_6_ absorber layer has a minor influence on the J_SC_ value, as indicated in Fig. 15(a). As the acceptor density of the lower absorber rises from $1\times {10}^{14} {\ cm}^{-3}$ to $1\times {10}^{19} {\ cm}^{-3}$, J_SC_ decreases from $26.08\ mA/{cm}^{2}$ to $25.7\ mA/{cm}^{2}$.

Nevertheless, V_OC_ is slightly increased by $0.35\%$ with the increase in acceptor concentration from $1\times {10}^{14}{\ cm}^{-3}$ to $1\times {10}^{19} {\ cm}^{-3}$, as indicated in Fig. 15(b). The PCE increases from $32.15\%$ at an N_A_ of $1\times {10}^{14} {\ cm}^{-3}$ to $32.72\%$ at $1\times {10}^{18} {\ cm}^{-3}$, then it reduces to $32.41\%$ at $1\times {10}^{19} {\ cm}^{-3}$, as presented in Fig. 15(c). Further, the value of FF is enhanced by $1.75\%$ as a result of increasing the acceptor concentration from $1\times {10}^{14} {\ cm}^{-3}$ to $1\times {10}^{19} {\ cm}^{-3}$. This behavior can be attributed to the rise in acceptor concentration, which strengthens the built-in electric field within the cell, and in turn facilitating effective separation of carriers and minimizing recombination losses. The enhanced electric field can also improve charge extraction, which results in a reduction in series resistance and the enhancement of performance parameters including FF, V_OC_, and PCE. Yet, it has been noted that increasing the acceptor doping concentration beyond $1\times {10}^{18} {\ cm}^{-3}$ can cause increased carrier recombination while reducing the carrier diffusion, which ultimately results in a decline in J_SC_ and PCE ^59^.

### Influence of interfacial defect density on device performance

Examining the junction interfaces of multi-junction SCs is an essential aspect because the use of different materials with varying crystal structures often leads to lattice mismatches between the interfaces of epitaxial films. Lattice mismatches can induce structural discontinuities at the interfaces, resulting in interfacial strain, dangling bonds, and misfit dislocations ^60^. Interfacial defects can act as centers of non-radiative recombination, which cause a reduction in carrier lifetime and consequently deteriorate the overall performance of the device. The velocities of electron and hole interfacial recombination can be determined using the following expressions ^61^:

11 $${V}_{n}={v}_{th}{N}_{t}{\sigma}_{n}$$

12 $${V}_{p}={v}_{th}{N}_{t}{\sigma}_{p}$$

where the carrier’s thermal velocity is denoted by ${v}_{th}$, and ${\sigma}_{n}$ and ${\sigma}_{p}$ are the electrons and holes capture cross sections, respectively, and ${N}_{t}$ denotes the interface defect density. In the proposed PSC configuration, the critical thicknesses of the epitaxial Cs_2_TiCl_6_ and Cs_2_AgBiI_6_ absorbers and the SnS_2_ ETL are $23.37\ \dot{A}$, $18.22\ \dot{A}$, and $2.77\ \dot{A}$, respectively. The relatively high mismatch factors and low critical thicknesses imply that the strained-layer epitaxy approach is unsuitable for this heterostructure, and that the resulting interfaces are not defect-free. Nevertheless, high-quality interfaces can still be achieved despite the significant lattice mismatch by employing experimental approaches such as surface modifications with functional group additives, which enhance the surface morphology and interfacial coupling with CTLs, resulting in improved device performance. Similarly, interface passivation with organic or inorganic materials can reduce interfacial defects and trap states, improving carrier transport. The employment of buffer layers with compatible lattice characteristics can also minimize lattice mismatch, which in turn improves long-term stability and device performance. Employing post-fabrication annealing has been shown to alleviate lattice strain and reduce defect concentrations, which enhances the perovskite microstructural quality. This approach, combined with surface and interface modification strategies, can facilitate the manufacturing of PSCs with appropriate interfacial properties even for structurally mismatched layers. Hence, while lattice mismatch is an inherent challenge, it does not pose a critical limitation to achieve high device performance, particularly under low-temperature processing conditions ^62^.

**Fig. 16:**
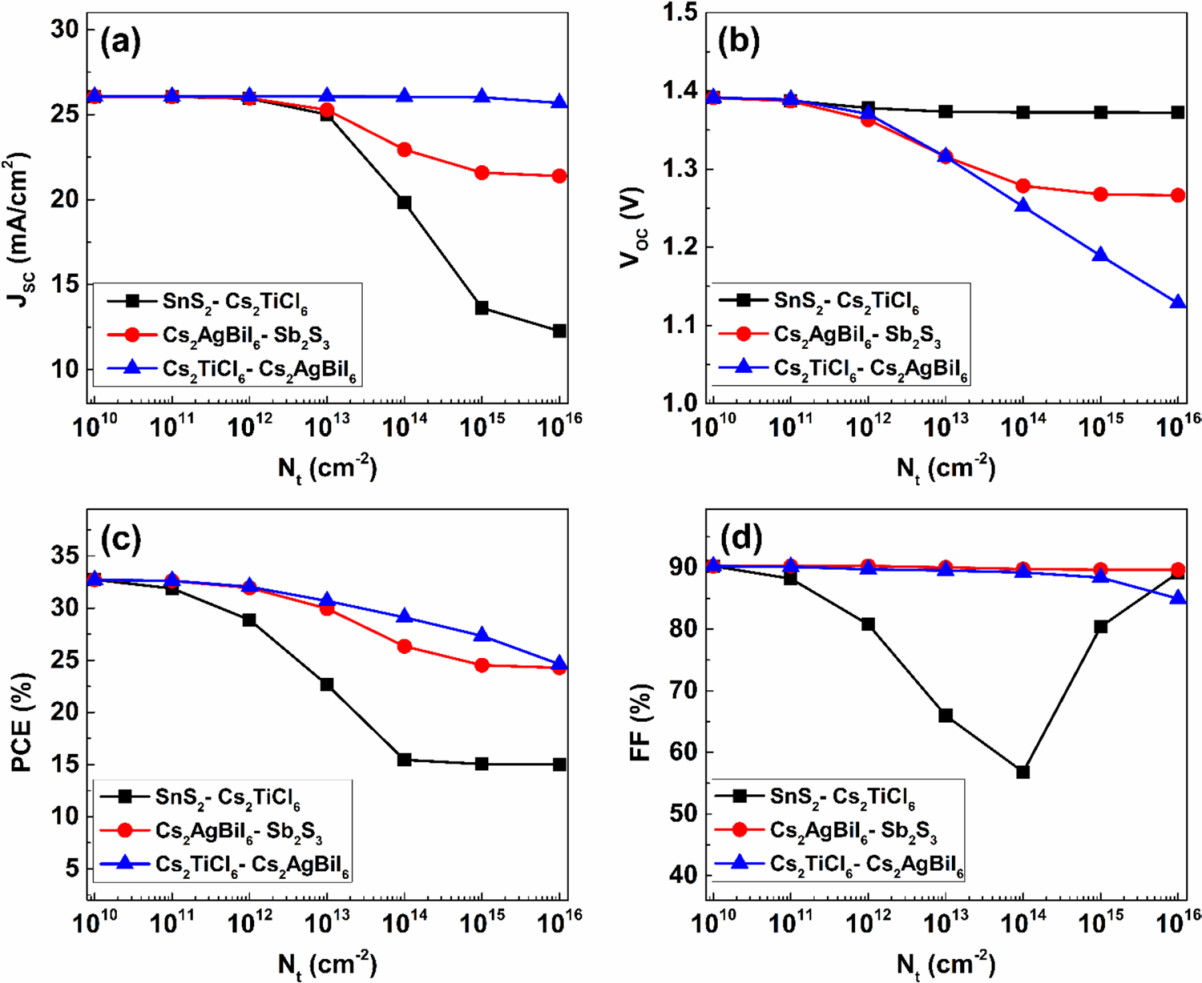
The effect of interfacial defect density on the electrical characteristics of the proposed device.

The effect of defect density at the ETL/ Cs_2_TiCl_6_, Cs_2_TiCl_6_/ Cs2AgBiI_6_, and Cs_2_AgBiI_6_ /HTL junction interfaces on the performance of the proposed PSC configuration is indicated by Fig. 16, where the defect density at each interface was ramped from $1\times {10}^{10} {\ cm}^{-2}$ to $1\times {10}^{16} {\ cm}^{-2}$. Figure 16 demonstrates that higher defect densities at any interface lead to performance deterioration due to the formation of excess carrier traps and recombination centers.

At the SnS_2_ layer contact with the upper Cs_2_TiCl_6_ absorber, increasing the interfacial defect density from $1\times {10}^{10} {\ cm}^{-2}$ to $1\times {10}^{16} {\ cm}^{-2}$ causes a reduction in J_SC_ by $52.92\%$, while it minimally impacts the value of V_OC_, causing a reduction of only $0.02\ V$ over the same defect density variation range. The value of PCE also drops to $15\%$ with the increase in density of defects to $1\times {10}^{16} {\ cm}^{-2}$. In addition, the FF value reduces from 90.25% with an N_t_ of $1\times {10}^{10} {\ cm}^{-2}$ to 65.97% with an N_t_ of $1\times {10}^{14} {\ cm}^{-2}$, then rises to $89.15\%$ at an N_t_ of $1\times {10}^{16} {\ cm}^{-2}$. Additionally, increasing the defect density at the interface of the lower Cs_2_AgBiI_6_ absorber with the Sb_2_S_3_ HTL from $1\times {10}^{10} {\ cm}^{-2}$ to $1\times {10}^{16} {\ cm}^{-2}$ causes a $4.68\ mA/{cm}^{2}$ reduction in the J_SC_ value, in addition to decreasing V_OC_ from $1.39\ V$ to $1.27\ V$, along with reducing PCE and FF from $32.72\%$ and $90.24\%$ to $24.28\%$ and $89.66\%$, respectively. Furthermore, the variation of defect density at the interface between the two absorbers has a minor impact on the value of J_SC_; nevertheless it causes the value of V_OC_ to decline from $1.39\ V$ to $1.13\ V$ as a result of the increased interface defect density from $1\times {10}^{10} {\ cm}^{-2}$ to $1\times {10}^{16} {\ cm}^{-2}$. Finally, increasing the interface density between the two absorbers to $1\times {10}^{16} {\ cm}^{-2}$ causes the diminution of PCE and FF to $24.62\%$ and $84.96\%$, respectively.

### Effect of metal electrode material on device performance

The choice of rear electrode material significantly governs the performance and long-term stability of PSCs. Moreover, since the electrode’s work function represents the energy required to liberate electrons from the electrode surface, it exerts a decisive influence on the charge extraction process and consequently on the device performance ^52^. Table 5 provides the electrical outputs of the proposed FTO/ SnS_2_/ Cs_2_TiCl_6_/ Cs_2_AgBiI_6_/ Sb_2_S_3_/ back contact, while nickel, gold, carbon, silver, and copper were employed as the back contact. The electrical performance of the introduced device exhibited a positive correlation with the work function of the rear electrode as presented in Table 5. While the results did not reveal any noticeable effect of the electrode’s work function on the value of J_SC_, the PCE rises from $27.67\%$ to $32.72\%$ in correspondence with the elevating work function from $4.6\ eV$ for copper to $5\ eV$ for gold. Additionally, the value of V_OC_ increases from $1.2 V$ to $1.39 V$ while FF rises from $88.77\%$ to $90.24\%$. These results can be interpreted by the increase in the work function of the electrode, which effectively reduces the barrier at the contact interface, promoting ohmic contact behavior and enhancing the extraction of majority charge carriers from the absorber layer ^56^.

**Table 5:** Effect of backward electrode material and work function on the electrical performance of the proposed PSC.

$\mathbf{M}\mathbf{a}\mathbf{t}\mathbf{e}\mathbf{r}\mathbf{i}\mathbf{a}\mathbf{l}$	$\mathbf{N}\mathbf{i}$	$\mathbf{A}\mathbf{u}$	$\mathbf{C}$	$\mathbf{A}\mathbf{g}$	$\mathbf{C}\mathbf{u}$
${\boldsymbol{W}}{\boldsymbol{o}}{\boldsymbol{r}}{\boldsymbol{k\ }}{\boldsymbol{f}}{\boldsymbol{u}}{\boldsymbol{n}}{\boldsymbol{c}}{\boldsymbol{t}}{\boldsymbol{i}}{\boldsymbol{o}}{\boldsymbol{n}}({\boldsymbol{e}}{\boldsymbol{V}})$	$5.15$	$5.1$	$5$	$4.7$	$4.6$
${\boldsymbol{P}}{\boldsymbol{C}}{\boldsymbol{E}}(\%)$	$32.72$	$32.72$	$32.72$	$30.2$	$27.67$
${{\boldsymbol{J}}}_{{\boldsymbol{S}}{\boldsymbol{C}}}({\boldsymbol{m}}{\boldsymbol{A}}/{{\boldsymbol{c}}{\boldsymbol{m}}}^{2})$	$26.06$	$26.06$	$26.06$	$26.06$	$26.06$
${{\boldsymbol{V}}}_{{\boldsymbol{O}}{\boldsymbol{C}}}({\boldsymbol{V}}{\boldsymbol{o}}{\boldsymbol{l}}{\boldsymbol{t}})$	$1.39$	$1.39$	$1.39$	$1.30$	$1.20$
${\boldsymbol{F}}{\boldsymbol{F}}(\%)$	$90.24$	$90.24$	$90.24$	$89.46$	$88.77$

In contrast, metals possessing lower work functions tend to exhibit Schottky contact behavior, resulting in higher potential barriers and diminished device efficiency ^20^. It is also notable that saturation of the performance of the proposed PSC occurred when the work function of the rear electrode surpassed $5\ eV,$ which highlights the potential of lower-cost alternatives, such as Ni and C, to replace expensive gold electrodes.

### Effect of temperature on device performance

**Fig. 17:**
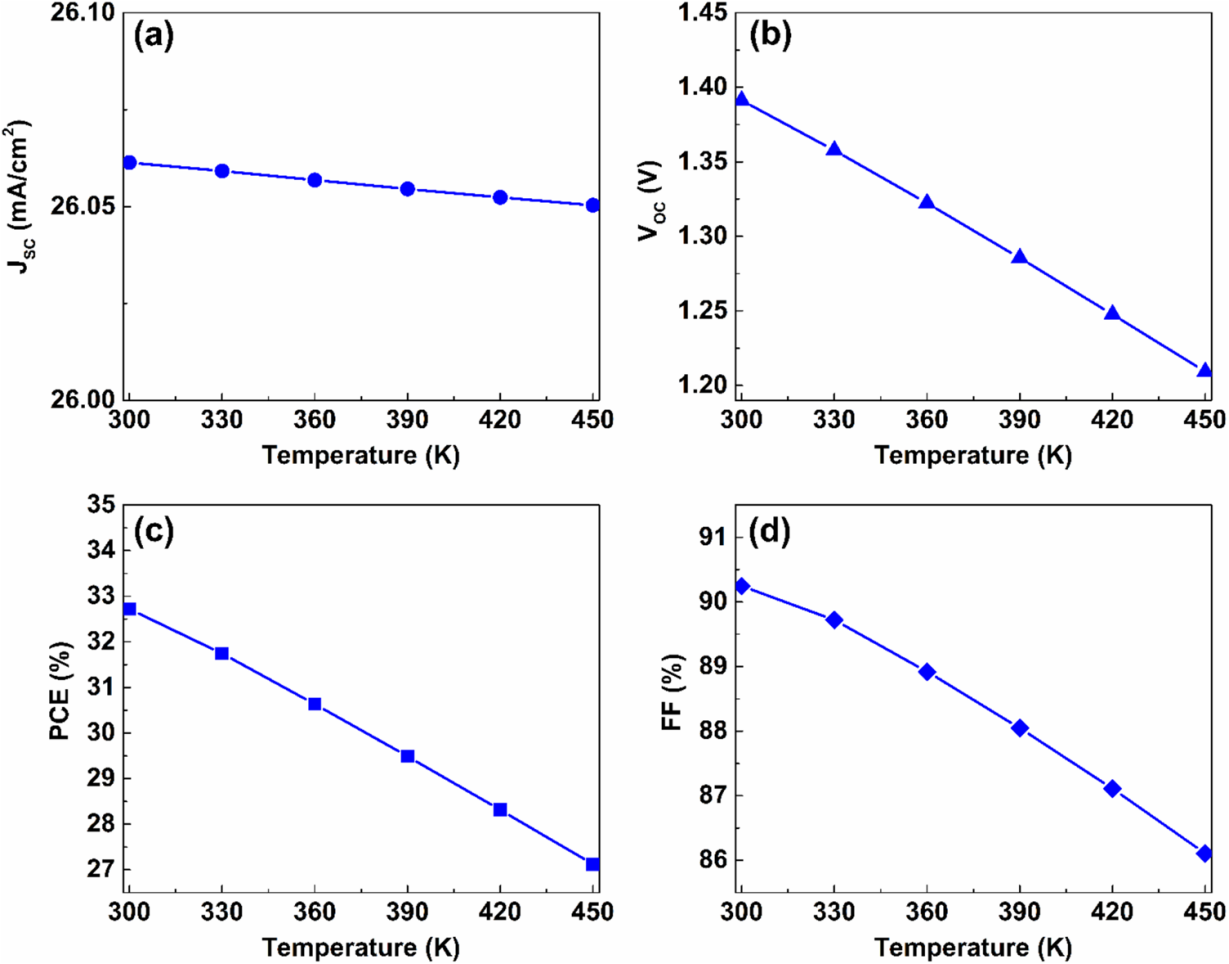
Effect of working temperature on the device electrical performance.

Solar cells in real-world operating environments can frequently be subjected to elevated temperatures that significantly influence their performance. Temperature variations can alter the energy bandgaps, carrier mobilities, and carrier concentrations of the utilized materials, which in turn affect the electrical behavior of the device ^52^. The impact of temperature on the photovoltaic performance was investigated by varying the operating temperature between $300 K$ and $450 K$. The variation in electrical parameters corresponding to the increase in temperature is shown in Fig. 17. As indicated in Fig. 17(a), the operating temperature has a negligible impact on J_SC_. However, V_OC_ decreases approximately by $0.2\ V$ when the operating temperature rises from $300 K$ to $450 K$, as shown in Fig. 17(b). This decline in V_OC_ can be attributed to the enhanced thermal velocity of carriers at elevated temperatures, which shortens the lifetime and diffusion length of charge carriers. In addition, high temperatures cause bandgap narrowing and alter the values of the effective densities of states, which also contribute to V_OC_ reduction. Furthermore, the additional thermal energy acquired by charge carriers can increase their recombination probability, which further degrades the device performance ^60^.

The effect of operating temperature on the value of the CB and VB densities of states of employed materials and on the thermal velocity of carriers can be quantitatively evaluated using the following equations ^63^:

13 $${N}_{c}\left(T\right)= {N}_{c}\left({T}_{o}\right)\times {\left(\frac{T}{{T}_{o}}\right)}^{1.5}$$

14 $${N}_{v}\left(T\right)= {N}_{v}\left({T}_{o}\right)\times {\left(\frac{T}{{T}_{o}}\right)}^{1.5}$$

15 $${V}_{th}\left(T\right)= {V}_{th}\left({T}_{o}\right)\times {\left(\frac{T}{{T}_{o}}\right)}^{1.5}$$

where ${T}_{o}$ is the standard operating temperature, conventionally assigned a value of $300 K$ in the simulation framework. Further, the variation of the semiconductor bandgap with temperature can be expressed as a function of temperature as follows ^42^:

16 $${E}_{g}\left(T\right)= {E}_{g}\left(0\right)-\frac{\alpha {T}^{2}}{(T+\beta )}$$

where $\alpha$ and $\beta$ are material-specific constants, and ${E}_{g}\left(0\right)$ and ${E}_{g}\left(T\right)$ represent the bandgap energies at $0 K$ and temperature $T$, respectively. The effect of operating temperature on V_OC_ can be analytically determined through the application of the following relation ^64^:

17 $${V}_{OC}=\frac{{n}_{id}KT}{q}\mathrm{log}\left( \frac{{J}_{SC}}{{J}_{o}}+1\right)$$

where ${J}_{o}$ denotes the saturation current density, its increase with temperature results in a corresponding decrease in V_OC_. ${n}_{id}$ represents the ideality factor. If ideality factor is equal to 2, then the governing recombination mechanism is SRH. Temperature elevation from $300 K$ to $450 K$ results in substantial reductions in PCE and FF, as shown in Figs. 17(c) and (d). The PCE decreases from $32.72\%$ to $27.12\%$, while the FF declines from $90.24\%$ to $86.1\%$. This degradation is caused by increased carrier recombination in the absorbers and at the interfaces, along with thermally induced stress and deformation that increase the series resistance, thereby impairing the device performance ^65^.

### Impact of parasitic resistances on device performance

Series resistance (R_S_) represents the cumulative resistance that opposes the flow of current through the interfaces of ETL/ absorber and absorber/ HTL junctions, as well as the resistive contributions from the front and rear electrodes. The interfacial and contact series resistances can be reduced through optimized fabrication methods and the employment of suitable interface modifiers. In addition, the bulk resistance can be further reduced by incorporating appropriate chemical compounds within ETL and HTL ^42^.

Shunt resistance (R_Sh_) is primarily related to induced defects during fabrication. Low R_Sh_ decreases the effective current flowing through the main cell structure by introducing alternative leakage pathways which deteriorate the device performance. Improving the structural design of the device can mitigate the shunt resistance effect and enhance the overall electrical behavior of the device ^66^.

The effect of R_S_ and R_Sh_ on the electrical behavior of SCs can be determined through the analytical formulation expressed as follows ^64^:

18 $${I}_{SC}{=I}_{o}\left({\mathrm{e}}^{\frac{qV}{{n}_{id}KT}}-1\right)-{I}_{Gen}-\frac{({V}_{OC}+{I}_{SC}{R}_{S})}{{R}_{Sh}}$$

where ${I}_{SC}$, ${I}_{o}$, and ${I}_{Gen}$ are the short-circuit, saturation and photogenerated currents, respectively.

**Fig. 18:**
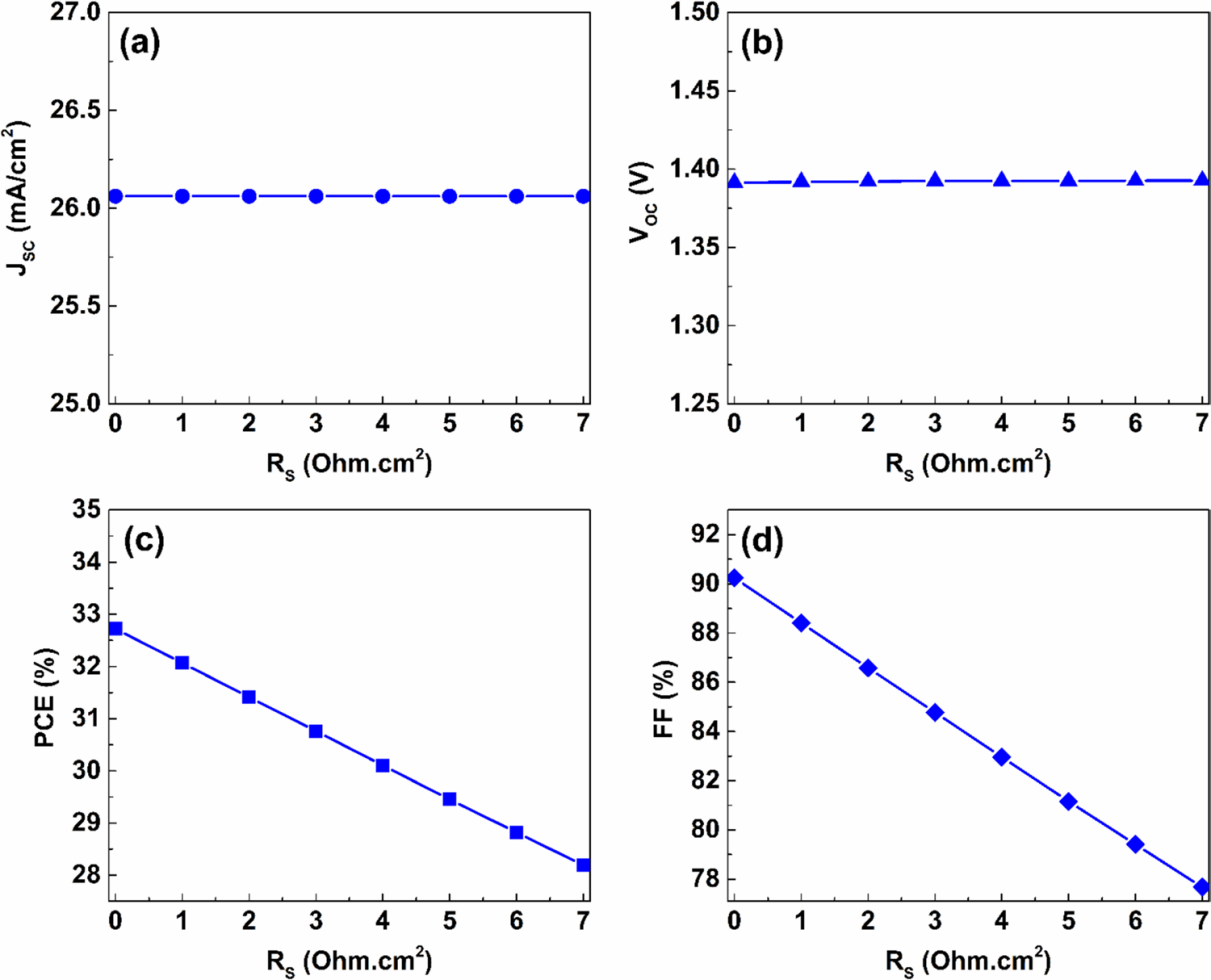
Impact of series resistance on device performance.

To assess the impact of R_S_ on the effectiveness of the introduced Cs_2_TiCl_6_/ Cs_2_AgBiI_6_ based device, R_S_ was systematically ramped from $0\ \Omega .{cm}^{2}$ to $7\ \Omega .{cm}^{2}$. As presented in Figs. 18(a) and (b), the variation of R_S_ exhibits a negligible influence on J_SC_ and V_OC_. Nevertheless, PCE and FF linearly reduce upon increasing R_S_, which indicates higher resistive losses and poorer charge extraction. PCE and FF drop from $32.72\%$ and $90.24\%$ to $28.19\%$ and $77.68\%$, respectively, when R_S_ increases from $0\ \Omega .{cm}^{2}$ to $7\ \Omega .{cm}^{2}$, as presented in Figs. 18(c) and (d).

**Fig. 19:**
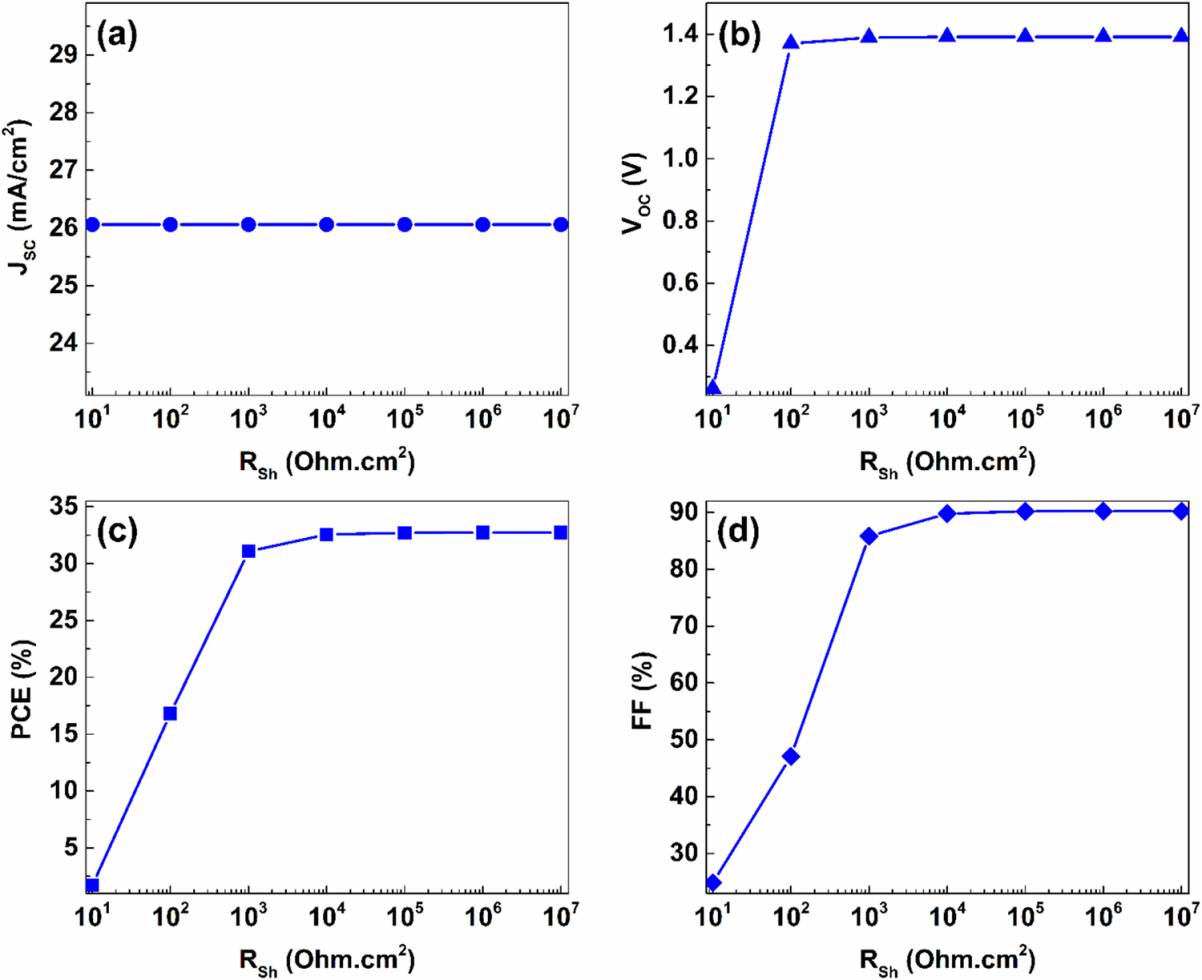
Impact of shunt resistance on device performance.

The effect of R_Sh_ value on photovoltaic characteristics of the introduced PSC was studied by varying R_Sh_ from $10\ \Omega .{cm}^{2}$ to ${10}^{7}\ \Omega .{cm}^{2}$. Increased R_Sh_ values effectively suppress leakage pathways and recombination at the junctions, resulting in improved carrier collection and overall performance, as presented in Fig. 19. As indicated in this figure, R_Sh_ has a negligible effect on the value of J_SC_. However, the V_OC_, PCE, and FF values exhibit substantial enhancements rising from $0.26\ V$, $1.69\%$ , and $24.86\%$ at ${R}_{Sh}=10\ \Omega .{cm}^{2}$ to $1.39\ V$ , $32.7\%$, and $90.2\%$ at ${R}_{Sh}={10}^{5}\ \Omega .{cm}^{2}$, respectively. Beyond this threshold, further R_Sh_ increments yield negligible performance gains, maintaining consistent values of J_SC_, V_OC_, PCE, and FF as $26.06\ mA/{cm}^{2}$, $1.39\ V$ , $32.72\%$ , and $90.24\%$, respectively.

### Evaluation of machine learning models

**Fig. 20:**
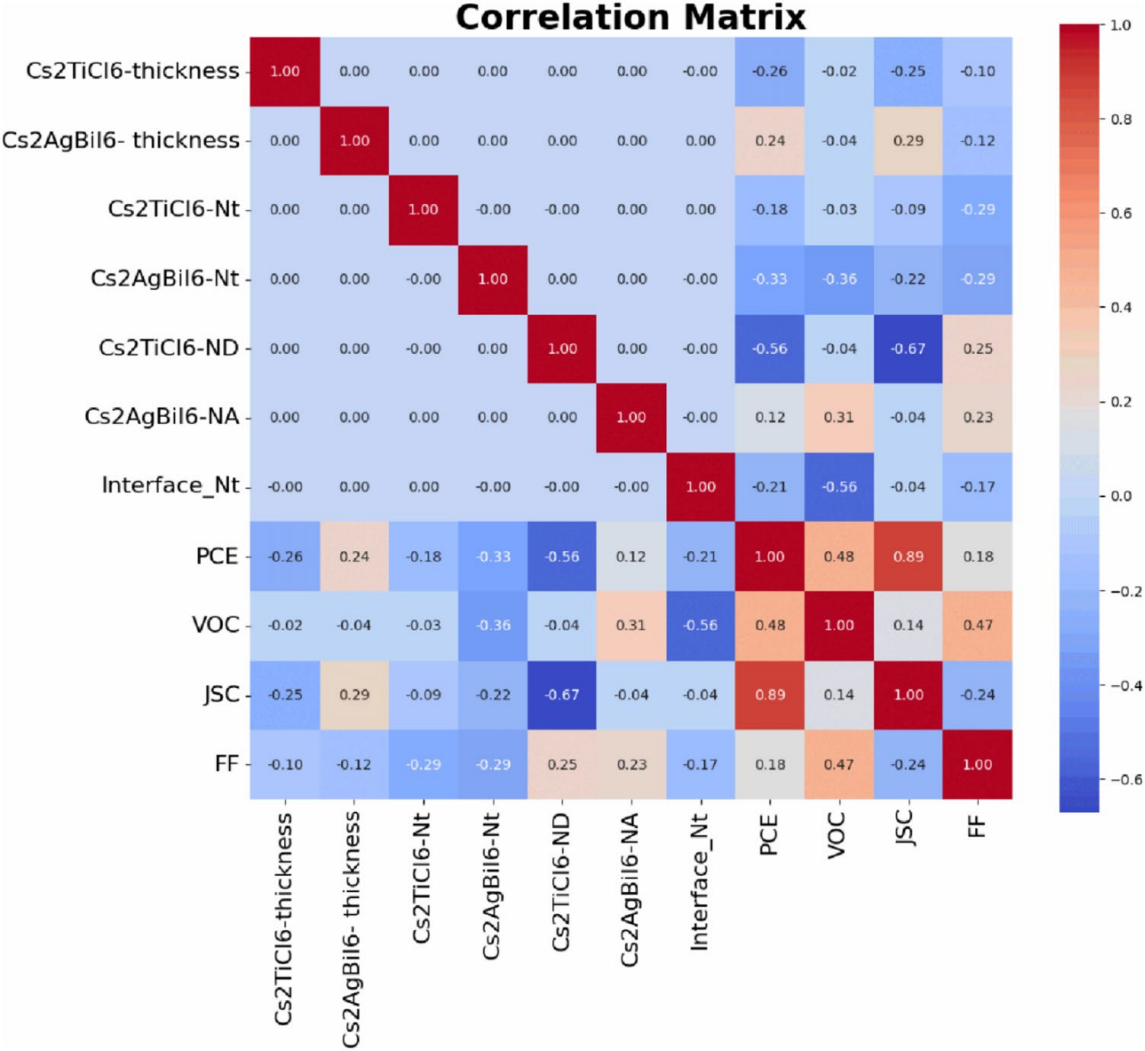
Feature correlation matrix derived from SCAPS-1D simulations.

Following the structure optimization of the proposed PSC and its key parameters to achieve maximum PCE, ML models were established to assist in performance prediction and future optimization. Simulation data were obtained through SCAPS-1D, incorporating critical physical fabrication-related parameters that influence device performance. Data preprocessing and organization were conducted in Python using Pandas and NumPy to ensure consistency, accuracy and reproducibility. The dataset comprises $2187$ output data points derived from detailed simulations by systematically varying key absorber parameters as the input variables, including absorber features, such as doping densities, defect concentrations and thickness of the two absorbers. A structured parametric sweep approach was implemented to ensure comprehensive coverage of input space and to capture possible nonlinear interactions between the parameters, as indicated by the generated dataset in Supplementary Data 2 (provided as a as CSV table) and the summary statistics of the dataset shown in Table S7 of the Supplementary Information. Output metrics were recorded to evaluate device performance comprehensively. Figure 20 depicts a correlation heatmap that illustrates the strength of linear relationships between the selected features and performance metrics. Furthermore, prior to the training of the ML models, the dataset was preprocessed to ensure consistency and enhance model performance. Given the wide variation in numerical ranges and physical units across features, normalization was employed to promote balanced model behavior and mitigate the influence of high-magnitude variables by bringing all variables into a comparable numerical range utilizing the StandardScaler transformation. It is significant to note that due to the strong physics-driven correlations in addition to utilizing the cross-validation schemes, this dataset size is adequate to capture the dominant nonlinear dependance governing the device performance.

The prepared dataset was utilized to train multiple ML algorithms including LR, KNN, SVR, RF, and XGB. The performance of ML models largely relies on the proper choice of hyperparameters. In this regard, the number of neighbors in KNN model should be carefully tuned to achieve an optimal balance between bias and variance, while distance weighting further enhances prediction accuracy. In SVR model, the use of the radial basis function (rbf) kernel allows effective modeling of nonlinear relationships, with the regularization parameter (C) governing the trade-off between model complexity and error tolerance. For ensemble methods, increasing the number of estimators in RF and XGB contributes to improved prediction stability. Additionally, key parameters such as maximum tree depth and learning rate in XGB were optimized to maintain high accuracy while avoiding overfitting. In this study, hyperparameter optimization was performed for each model using a grid search approach combined with fivefold cross-validation (CV), which ensured reliable evaluation of model generalization capability and guided hyperparameter tuning ^31^. In addition, K-fold CV involves dividing the dataset into $k$ equal parts, in this study $k=5$ , with the model trained sequentially on $k-1$ parts and tested on the remaining part which ensures enhanced model robustness ^67^.

The final set of hyperparameters, presented in Table 6, delivered optimal performance for PCE prediction.

**Table 6:** Utilized hyperparameters of the employed ML models.

${\boldsymbol{M}}{\boldsymbol{o}}{\boldsymbol{d}}{\boldsymbol{e}}{\boldsymbol{l}}$	${\boldsymbol{P}}{\boldsymbol{a}}{\boldsymbol{r}}{\boldsymbol{a}}{\boldsymbol{m}}{\boldsymbol{e}}{\boldsymbol{t}}{\boldsymbol{e}}{\boldsymbol{r}}$	${\boldsymbol{V}}{\boldsymbol{a}}{\boldsymbol{l}}{\boldsymbol{u}}{\boldsymbol{e}}$
LR	$alpha$	$10$
	$solver$	$svd$
KNN	$n\_neighbors$	$11$
	$weights$	$distance$
	$p$	$1$
SVR	$C$	$100$
	$epsilon$	$0.01$
	$kernel$	$rbf$
	$gamma$	$auto$
RF	$n\_estimators$	$200$
	$max\_depth$	$none$
	$min\_samples\_split$	$2$
	$min\_samples\_leaf$	$1$
	$max\_features$	$sqrt$
XGB	$n\_estimators$	$200$
	$max\_depth$	$5$
	$learning\_rate$	$0.1$
	$subsample$	$0.8$
	$colsample\_bytree$	$0.8$
	$gamma$	$0$
	$min\_child\_weight$	$5$

**Fig. 21:**
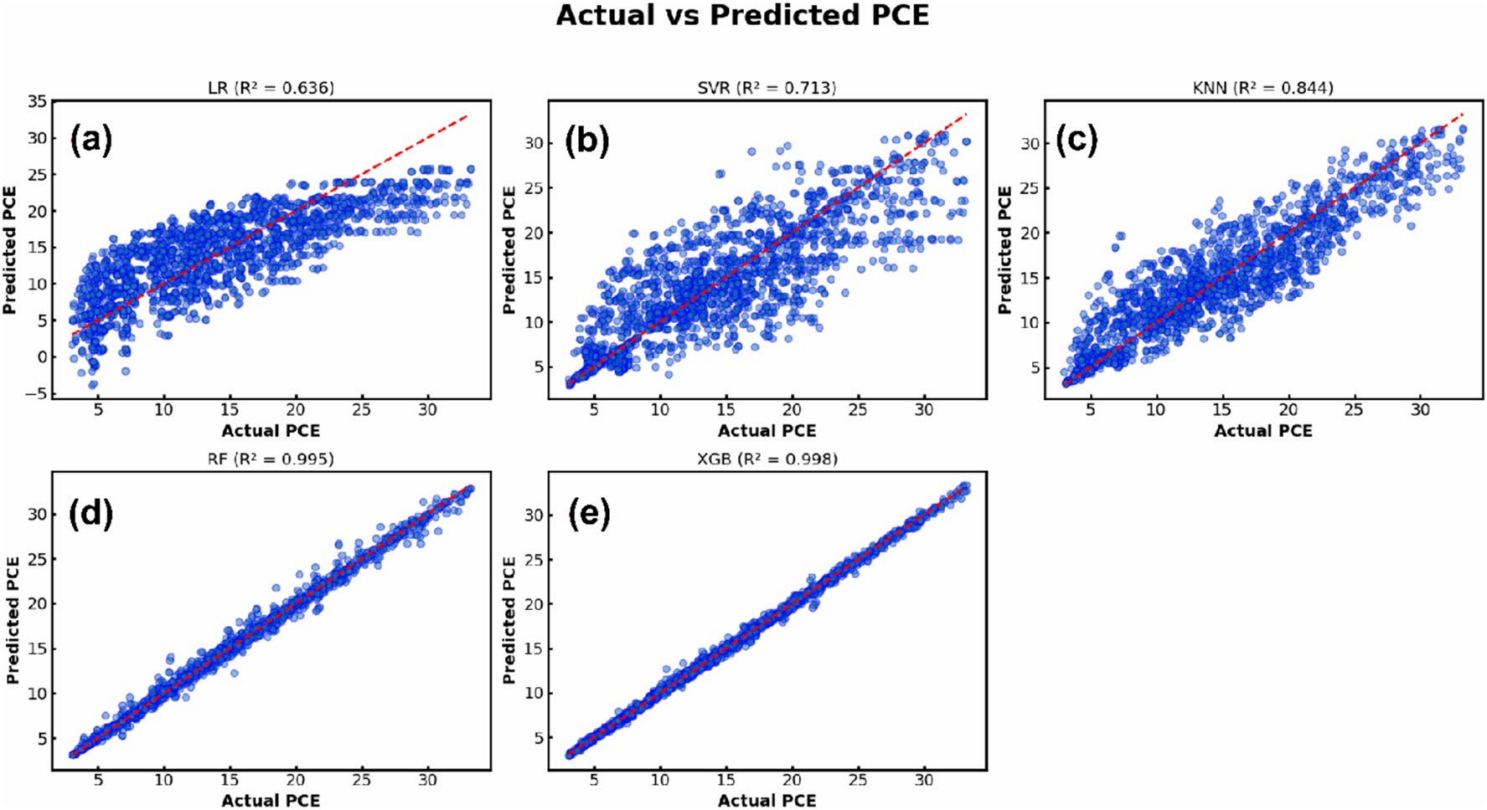
Comparison of predicted and actual PCE using **(a)** LR **(b)** SVR **(c)** KNN **(d)** RF **(e)** XGB algorithms.

Standard measures, including $MAE$, $MSE$, $RMSE$, and ${R}^{2}$, were utilized to evaluate the predictive capability of the employed algorithms and to provide quantitative insights into the models’ prediction accuracy. The objective of this part of the study is to select an ML model that can achieve minimal prediction errors, indicated by a low $RMSE$ value, while maintaining high correlation between observed and predicted values, reflected by a high value of ${R}^{2}$. Table 7 and Fig. 21 summarize the predictive performance of the evaluated ML models for the different photovoltaic parameters of the proposed device. Table 7 indicates the standard out of fold measures of $MAE$, $MSE$, $RMSE$, and ${R}^{2}$ for the different target parameters corresponding to each utilized model, while Fig. 21 presents comparison of predicted and actual PCE using the different employed algorithms. In addition, Figs. S7-S11 of the Supplementary Information present complete comparisons of predicted and actual values of all target variables using the different employed algorithms. As provided in Table 7 and Fig. 21(a), the LR model indicates poor predictive capability when applied to the utilized dataset. This model was unable to accurately capture the underlying data patterns, as evidenced by the low ${R}^{2}$ values of $0.6362$, $0.5415$, $0.6520$, and $0.3282$, and high $RMSE$ values of $4.2222$, $0.0930$, $3.9320$, and $7.9226$ for PCE, V_OC_, J_SC_, and FF, respectively.

**Table 7:** Assessment of various ML models for predicting key photovoltaic performance metrics.

${\boldsymbol{T}}{\boldsymbol{a}}{\boldsymbol{r}}{\boldsymbol{g}}{\boldsymbol{e}}{\boldsymbol{t}}$ ${\boldsymbol{V}}{\boldsymbol{a}}{\boldsymbol{r}}{\boldsymbol{i}}{\boldsymbol{a}}{\boldsymbol{b}}{\boldsymbol{l}}{\boldsymbol{e}}$	${\boldsymbol{M}}{\boldsymbol{o}}{\boldsymbol{d}}{\boldsymbol{e}}{\boldsymbol{l}}$	${\boldsymbol{M}}{\boldsymbol{S}}{\boldsymbol{E}}$	${\boldsymbol{M}}{\boldsymbol{A}}{\boldsymbol{E}}$	${\boldsymbol{R}}{\boldsymbol{M}}{\boldsymbol{S}}{\boldsymbol{E}}$	${{\boldsymbol{R}}}^{2}$
$PCE$	LR	$17.8273$	$3.5023$	$4.2222$	$0.6362$
	RF	$0.2494$	$0.2812$	$0.4994$	$0.9949$
	KNN	$7.6364$	$2.0320$	$2.7634$	$0.8442$
	SVR	$14.0590$	$2.6015$	$3.7495$	$0.7131$
	XGB	$0.0859$	$0.1999$	$0.2931$	$0.9982$
${V}_{OC}$	LR	$0.0087$	$0.0767$	$0.0930$	$0.5415$
	RF	$0.0000$	$0.0034$	$0.0060$	$0.9981$
	KNN	$0.0040$	$0.0495$	$0.0629$	$0.7902$
	SVR	$0.0046$	$0.0496$	$0.0677$	$0.7568$
	XGB	$0.0000$	$0.0043$	$0.0060$	$0.9981$
${J}_{SC}$	LR	$15.4608$	$3.2318$	$3.9320$	$0.6520$
	RF	$0.0321$	$0.0711$	$0.1791$	$0.9993$
	KNN	$9.8114$	$2.1925$	$3.1323$	$0.7792$
	SVR	$19.4499$	$2.8400$	$4.4102$	$0.5622$
	XGB	$0.0130$	$0.0693$	$0.1140$	$0.9997$
$FF$	LR	$62.7671$	$5.8926$	$7.9226$	$0.3282$
	RF	$1.0668$	$0.4289$	$1.0329$	$0.9886$
	KNN	$40.3807$	$4.0401$	$6.3546$	$0.5678$
	SVR	$60.2620$	$4.6898$	$7.7629$	$0.3550$
	XGB	$0.5912$	$0.4437$	$0.7689$	$0.9937$

The reliance of the SVR model on tolerance parameters makes it less suitable for handling datasets beyond small sizes. Although kernel transformations were applied, this model failed to accurately capture the underlying data patterns, as indicated in Fig. 21(b) and confirmed by the high $RMSE$ and low ${R}^{2}$ values corresponding to the different target variables. The KNN model showed a more reliable predictive capability, as indicated in Fig. 21(c), with higher ${R}^{2}$ and lower $RMSE$ values. On the other hand, the RF model, employing an ensemble of decision trees to enhance generalization and minimize overfitting, yeilded exceptional predictive accuracy, as indicated in Fig. 21(d). This was reflected by a high ${R}^{2}$ value of $0.9949$ and a low $RMSE$ value of $0.4994$ when predicting PCE. The XGB algorithm was found to possess the most effective prediction capability among the evaluated models. The tree-building process in XGB models uses gradient descent to reduce errors and improve overall prediction accuracy, as indicated in Fig. 21(e). The $RMSE$ and ${R}^{2}$ values yielded by the XGB model were found to be $0.2931$ and $0.9982$ for PCE prediction, $0.0060$ and $0.9981$ for V_OC_, $0.1140$ and $0.9997$ for J_SC_, $0.7689$ and $0.9937$ for FF.

**Fig. 22:**
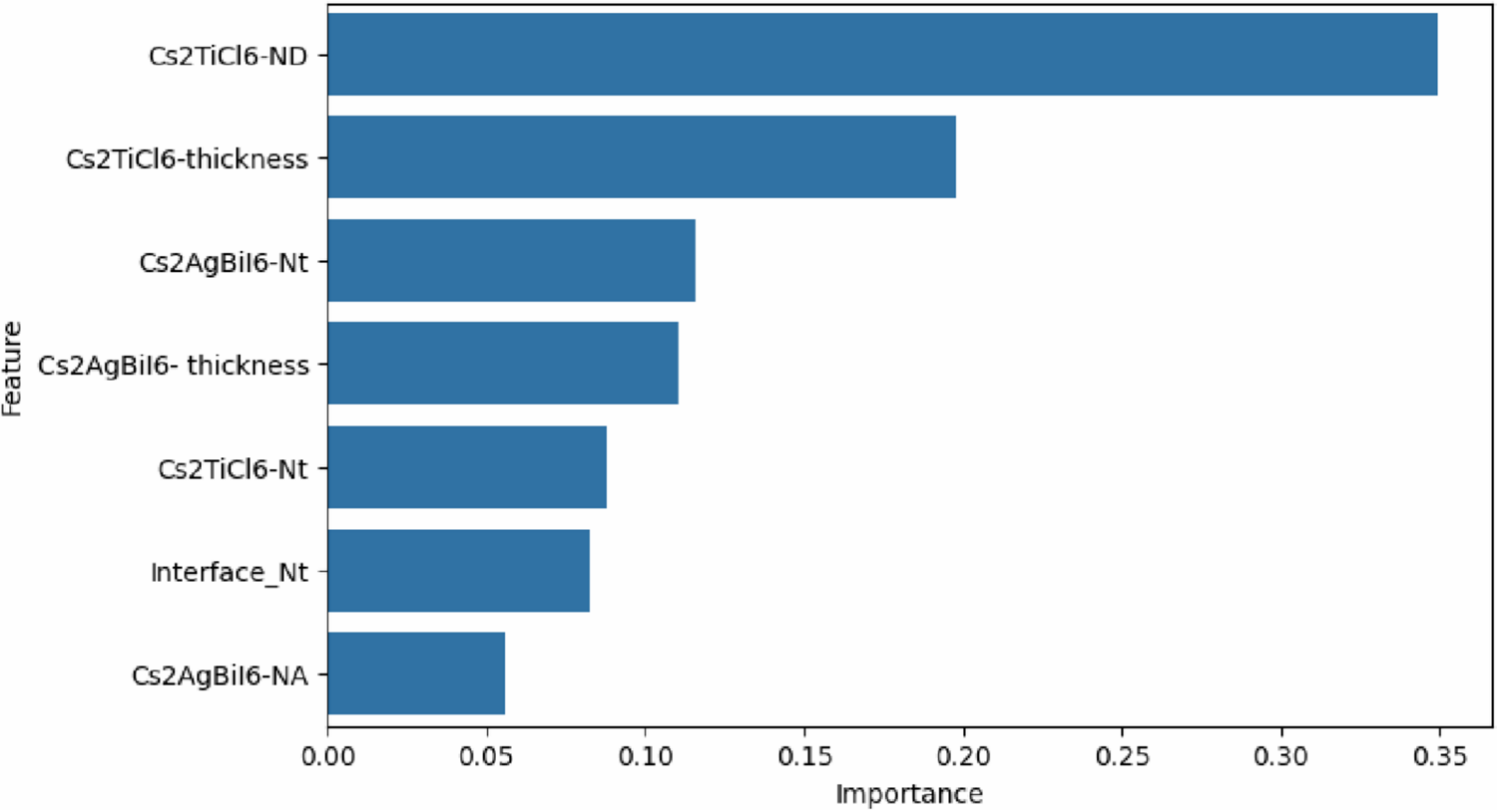
Bar chart representation of feature importance for the proposed PSC derived from the XGB model.

Further, The XGB model was employed to determine the key factors influencing performance of the proposed device. To achieve this, two complementary methods were utilized: the intrinsic feature importance measure and the more detaled SHAP analysis. Figure 22 shows the intrinsic feature importance presenting the relative contribution and influence direction of each input feature on the PCE prediction made by XGB model. As illustrated in Fig. 22, the doping density and the thickness of the Cs_2_TiCl_6_ upper absorberhave the most pronounced influence on PCE prediction.

**Fig. 23:**
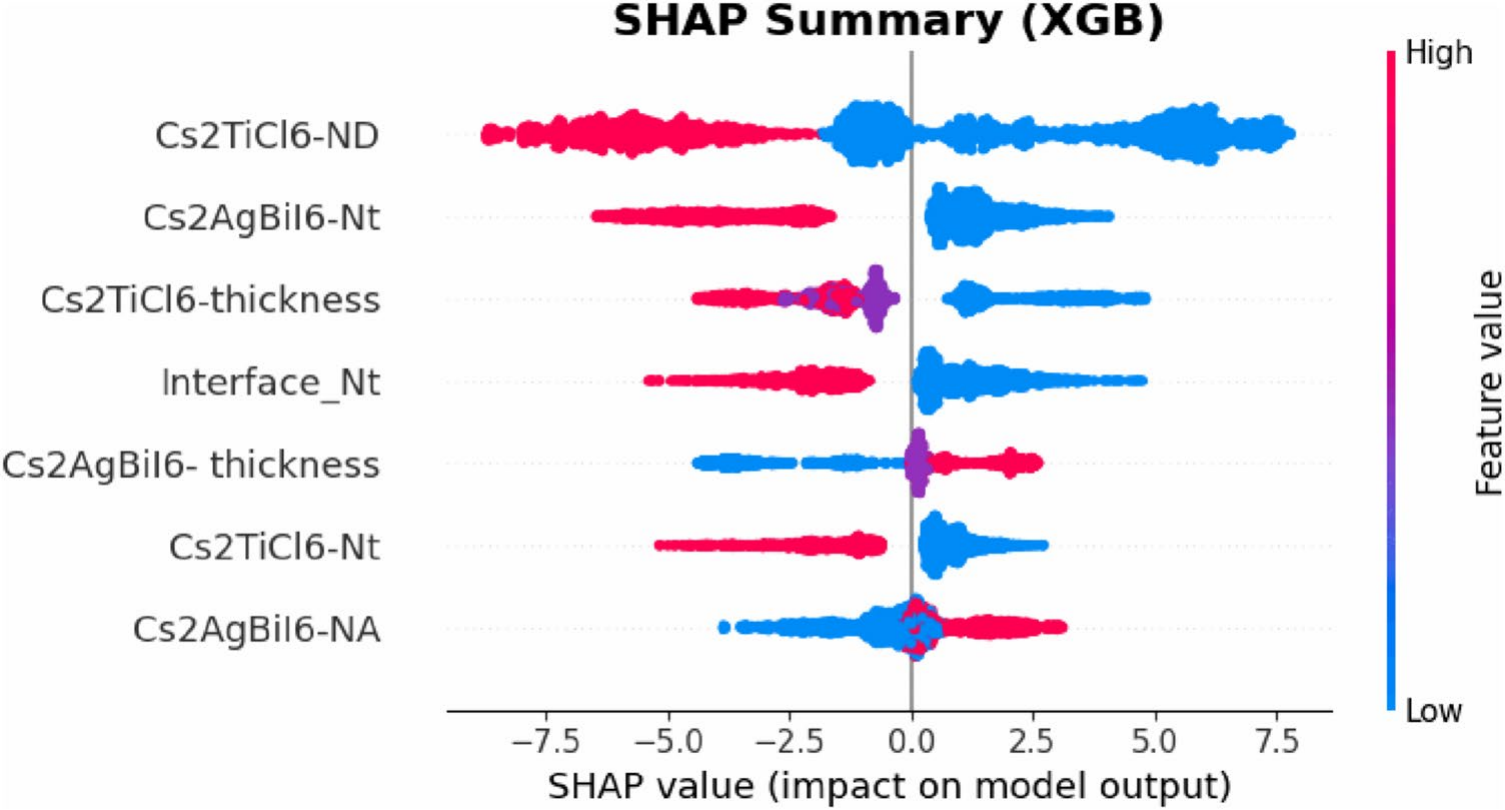
SHAP summary plot illustrating the contribution of individual features to estimate PCE utilizing XGB model.

Furthermore, Feature importance analysis using SHAP was conducted for the XGB model to guarantee a deeper understanding of factors influencing model predictions. While ordinary feature importance analysis only identifies dominant parameters, it cannot determine how they impact the performance. On the other hand, the SHAP approach overcomes this limitation by determining whether each feature exerts a positive or negative influence, providing a clearer understanding of the correlation between absorber parameters and photovoltaic target parameters ^31^. Figure 23 provides the SHAP summary plot, representing the relative contribution and influence direction of each input feature on the PCE prediction made by XGB model. As indicated by Fig. 23, the donor doping density of the Cs_2_TiCl_6_ upper absorber, the defect density of the Cs_2_AgBiI_6_ layer, the Cs_2_TiCl_6_ upper absorber thickness, and the interface defect density between the Cs_2_TiCl_6_ and Cs_2_AgBiI_6_ absorbers exhibit the most significant impacts on PCE prediction. In contrast, the thickness of Cs_2_AgBiI_6_, the bulk defect density of Cs_2_TiCl_6_, and the doping concentration of the Cs_2_AgBiI_6_ lower absorber have a lesser effect on PCE prediction. The SHAP distribution indicates that decreasing the doping density and thickness of the Cs_2_TiCl_6_ upper absorber can effectively improve PCE. Furthermore, reducing interfacial defect density and the defect densities of both absorbers can also enhance PCE, corresponding to the blue-shaded region with higher SHAP values. In contrast, increasing the thickness and doping density of the Cs_2_AgBiI_6_ lower absorber elevates PCE.

A key limitation of the presented ML framework is that the dataset was generated by varying only absorber-related parameters while keeping the properties of CTLs fixed at their optimized values. Consequently, the trained models are most reliable within the constrained parameter space and may not directly extend to substantially different device configurations. Although the proposed ML framework demonstrates strong predictive performance on SCAPS-1D generated data, its direct applicability to experimental datasets remains limited due to inherent differences between simulated and real-world device conditions. In practical devices, additional effects such as fabrication-induced defects, environmental degradation, and measurements uncertainties play a significant role, which are not fully captured in numerical simulation.

Therefore, the presented models should be regarded as physics-informed surrogate models intended to guide theoretical optimization within the simulated parameter space. Future work will therefore aim to broaden the dataset by incorporating additional layers and experimental variability to enhance model robustness and overall applicability. In addition, integrating experimental datasets will be crucial for future validating and improving the real-world relevance of the proposed approach.

#### Fabrication tolerance analysis

**Fig. 24:**
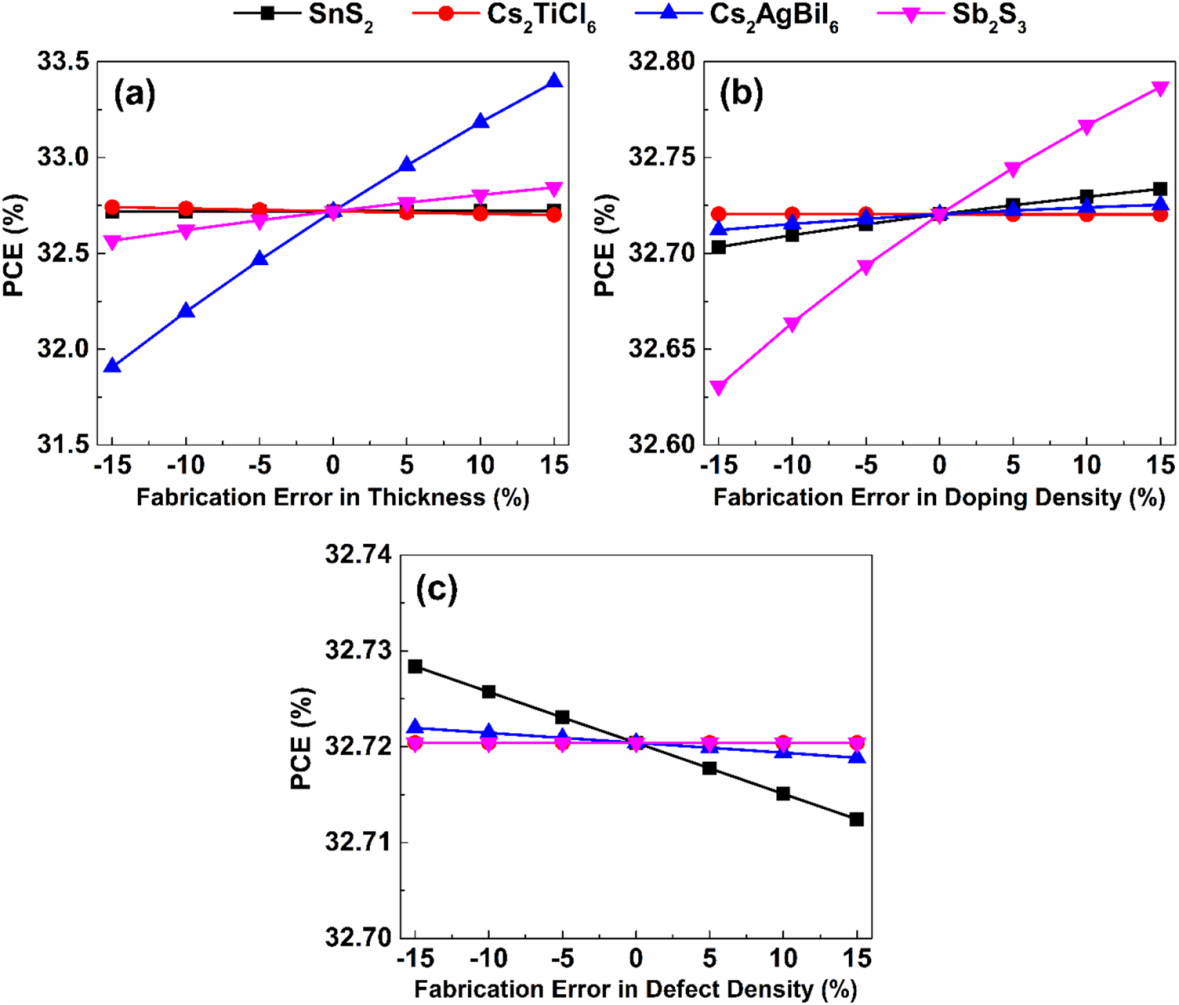
Predicted PCE variation corresponding to deviations in (**a**) the thickness (**b**) the doping density (**c**) the defect density of the utilized layers.

While the present study is numerical in nature, potential fabrication inaccuracies, particularly the variation in the thickness, doping concentrations, and defect densities of the utilized absorbers and charge transport layers, were incorporated into the analysis to evaluate their impact on the PSC’s performance. As shown in Fig. 24a, the thickness deviation of the SnS_2_ ETL by $\pm 15\%$ from $500\ nm$ has a negligible influence on the PCE of the introduced Cs_2_TiCl_6_-Cs_2_AgBiI_6_ bi-absorber device. Furthermore, a thickness deviation of the Cs_2_TiCl_6_ layer by $15\%$ and $-15\%$ from its nominal $100\ nm$ value produces only a $0.04\%$ marginal change in PCE. In addition, a deviation in the thickness of the lower absorber up to $15\%$ from the nominal thickness of $1000\ nm$ increases the PCE by only $0.67\%$. Moreover, the variation of the Sb_2_S_3_ HTL thickness within the same deviation range exhibits a $0.27\%$ variation in the PCE value. Such minor variations in PCE values over $\pm 15\%$ deviation of the layers’ thicknesses indicate the device’s robustness to small thickness variations.

On the other hand, the influence of doping density deviations for the utilized layers is presented in Fig. 24(b). As shown in this figure, a $\pm 15\%$ deviation in the layers’ doping densities from their nominal optimized values has only a minor influence on the performance of the introduced device, and consequently on its PCE. The variation of the acceptor doping density of the Sb_2_S_3_ layer by $\pm 15\%$ from its nominal value of $1\times {10}^{21} {\ cm}^{-3}$ causes a negligible, though measurable, effect on the PCE, which varies by only $0.14\%$. Further, the device exhibits minimal sensitivity to $\pm 15\%$ variations in layers’ defect density, with only a slight effect observed in both performance and PCE, as indicated in Fig. 24(c). Figures S12-S14 of the Supplementary Information indicate the detailed influence of variations in thicknesses, defect densities, and doping concentrations of absorbers and CTLs on the levels of J_SC_, V_OC_, PCE and FF. The findings of the presented fabrication tolerance analysis demonstrate that the proposed dual-absorber PSC exhibits a high degree of tolerance to deviations in structural parameters.

#### Comparison with Literature and Study Limitations

According to Zhu et al. ^23^, bi-absorber PSCs can successfully be manufactured through controlled deposition process. Their framework integrates the method of in-situ growth, wherein an organic ammonium salt solution was spin coated onto an existing 3D perovskite film then precise annealing was employed, along with solvent-free deposition method such as vapor processing yielding low-dimensional covering layer that can significantly improve the quality of interfaces and charge collection which are critical factors that govern the efficiency and stability of device. Although the present analysis is based on numerical simulation, the experimental strategies proposed by Zhu et al. provided a solid foundation for future experimental verification and optimization. In addition, the theoretical Shockley-Queisser detailed balance analysis indicates that full tandem perovskite SC employing Cs_2_TiCl_6_ and Cs_2_AgBiI_6_ absorbers with bandgaps of $2.23\ eV$ and $1.6\ eV$, respectively can achieve PCE up to $34 \%$, theoretically ^68^. The proposed half-tandem Cs_2_TiCl_6_/ Cs_2_AgBiI_6_ PSC offers optimal trade-off between the structural simplicity and high theoretical PCE. The proposed device benefits from the absence of current-matching requirement between the sub-cells in full tandem cells.

To clearly explain the mechanism behind the performance enhancement, the improvement in PCE of the proposed lead-free Cs_2_TiCl_6_/ Cs_2_AgBiI_6_ PSC has been achieved through a comprehensive multi-parameter optimization approach rather than relying on a single variable. In this study, simulations were initially used to investigate the impact of employing several CTLs, then the combined influence of absorber thickness, doping density, defect concentration, and interface defect states on the device performance were investigated. This approach helps determine the optimal physical conditions that simultaneously boost charge generation, minimize bulk and interfacial recombination losses, and enhance charge carrier transport through the device. The results of numerical analysis revealed that employing high-quality perovskites, characterized by fewer defects and appropriate energy-level alignment with the CTLs, effectively suppresses non-radiative recombination, leading to longer carrier lifetimes. Moreover, optimizing the absorber thickness ensures adequate light absorption while preventing increased recombination associated with longer carrier transport paths. Further, adjusting doping levels in both absorber layers improves the built-in electric field, facilitating better charge separation and extraction. These optimized physical parameters are subsequently analyzed using ML models to locate high-performance regions within the multidimensional parameter space, enabling efficient prediction and targeted optimization of device configurations for maximum PCE.

**Fig. 25:**
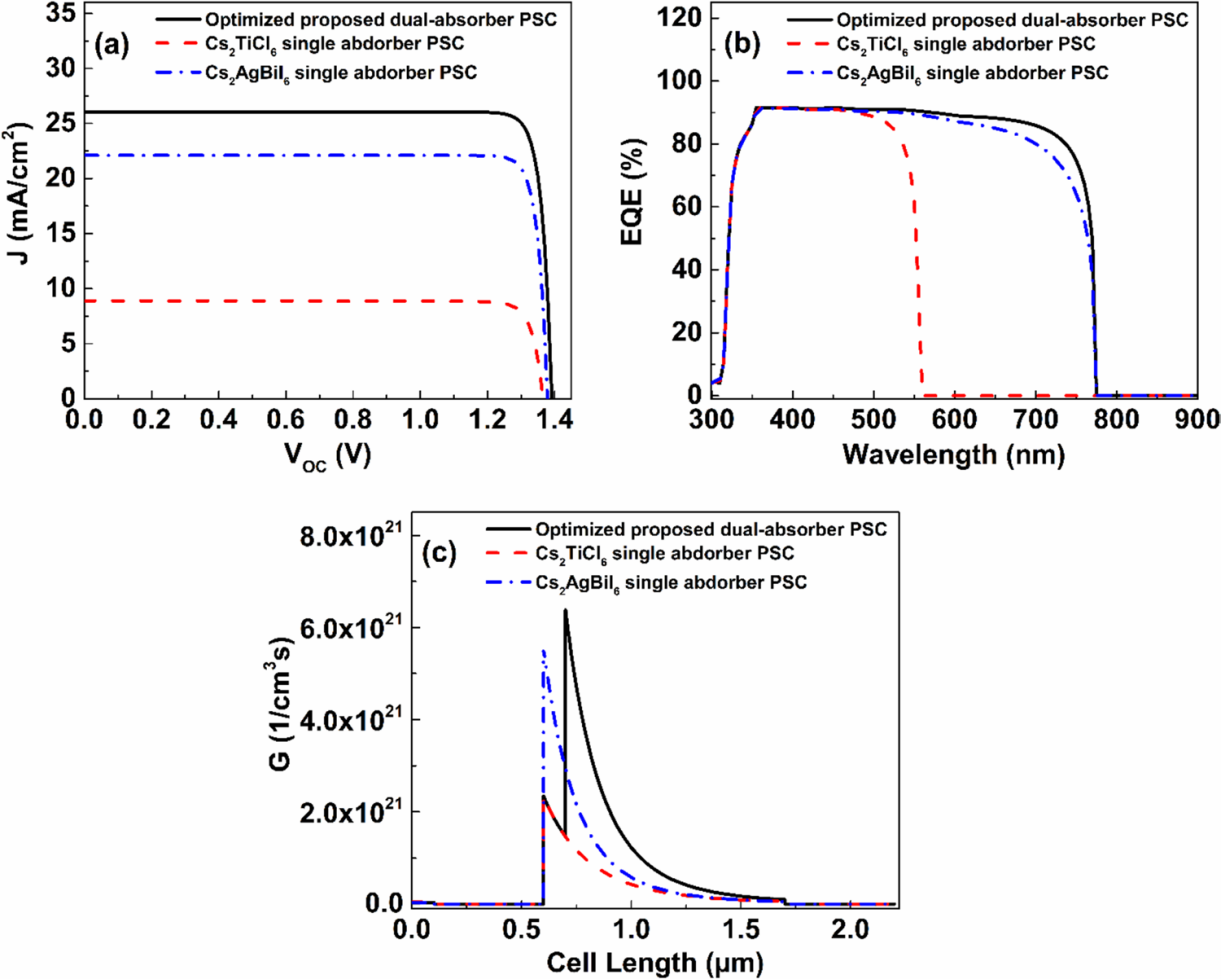
**(a)** J-V characteristics and **(b)** EQE spectra **(c)** Carrier generation rate profile for proposed dual-absorber PSC and reference single absorber-based cells.

Figure 25 compares the current density- voltage (J-V) characteristics, external quantum efficiency (EQE) spectra, and carrier generation rates of the proposed dual-absorber Cs_2_TiCl_6_/ Cs_2_AgBiI_6_ PSC with those of the single absorber Cs_2_TiCl_6_ and Cs_2_AgBiI_6_ based reference cells, while maintaining identical structural configurations.

From the data presented in Fig. 25(a), it can be discerned that the proposed optimized dual-absorber PSC configuration, incorporating a Cs_2_TiCl_6_ absorber above a Cs_2_AgBiI_6_ layer, yielded superior performance. It achieved a V_OC_ of $1.39\ V$, a J_SC_ of $26.06\ mA/{cm}^{2},$ an FF of $90.24\%$, and a PCE of $32.72\%$. In contrast, the single-absorber Cs_2_TiCl_6_ and Cs_2_AgBiI_6_ based PSCs exhibited suboptimal performances; their J_SC_ and V_OC_ were $8.89\ mA/{cm}^{2}$ and $1.36\ V$ for Cs_2_TiCl_6_, and $22.11\ mA/{cm}^{2}$ and $1.39\ V$ for Cs_2_AgBiI_6_. The single-absorber Cs_2_TiCl_6_ achieved a PCE of $10.81\%$, while the Cs_2_AgBiI_6_ single-absorber PSC yielded a PCE of $27.84\%$. The EQE curve of the single-absorber Cs_2_TiCl_6_ based PSC, indicated in Fig. 25(b), spans a substantial portion of the visible region, expanding from $350\ nm$ to approximately $556\ nm$. On the other hand, the EQE spectra of both the single Cs_2_AgBiI_6_ absorber-based PSC and the introduced dual-absorber Cs_2_TiCl_6_/ Cs_2_AgBiI_6_ extends to $775\ nm$. Notably, the spectral EQE profile of the suggested bi-absorber Cs_2_TiCl_6_/ Cs_2_AgBiI_6_ indicates higher quantum efficiency within the $550-770\ nm$ range. However, the EQE curves for both the single Cs_2_AgBiI_6_ absorber and the introduced dual-absorber Cs_2_TiCl_6_/ Cs_2_AgBiI_6_ drop to $0\%$ beyond $775\ nm$. The superior QE of bilayer PSC demonstrates improved photon absorption and carrier generation over single-layer designs. Consequently, the improved performance of the proposed Cs_2_TiCl_6_/ Cs_2_AgBiI_6_ dual-absorber PSC leads to an increase in PCE by $21.91\%$ and $4.88\%$ over the single-absorber Cs_2_TiCl_6_ and Cs_2_AgBiI_6_ based PSCs, respectively. Additionally, Fig. 25(c) shows that the generation rate profile of the proposed dual-absorber PSC follows the typical trend, with higher carrier generation near the top surface of each absorber layer that gradually decreases with depth in accordance with the extinction coefficient of each material. The figure also demonstrates that the proposed dual-absorber device exhibits superior carrier generation rates compared to single-layer designs, reaching a peak exceeding $6\times {10}^{21} {\ cm}^{-3}s$ at the interface between the two absorber layers, indicating enhanced photon absorption enabled by the incorporation of the lower Cs_2_AgBiI_6_ absorber.

Table 8 shows a comparative evaluation of several dual-absorber PSC reported designs, highlighting critical photovoltaic characteristics including V_OC_, J_SC_, FF, and PCE. Presented data reveals how different absorber compositions and layer arrangements influence device efficiency. Among the listed studies, this work achieves exceptional performance with PCE of $32.72 \%$. Such results underscore the capability of the proposed dual-absorber approach to advance PSC performance beyond other existing designs.

**Table 8:** Comparison of electrical parameters obtained in the present work with values reported in earlier studies.

**Structure**	${\boldsymbol{P}}{\boldsymbol{C}}{\boldsymbol{E}}$ $(\%)$	${{\boldsymbol{J}}}_{{\boldsymbol{S}}{\boldsymbol{C}}}$ $({\boldsymbol{m}}{\boldsymbol{A}}/{\boldsymbol{c}}{\boldsymbol{m}})$	${{\boldsymbol{V}}}_{{\boldsymbol{O}}{\boldsymbol{C}}}$ $({\boldsymbol{V}})$	${\boldsymbol{F}}{\boldsymbol{F}}$ $(\%)$	**Ref**
FTO/ NiO_x_/ BA_2_MA_3_Pb_4_I_13_/ MAPbI_3_/ C60/ Au	$28.24$	$23.15$	$1.37$	$89.2$	^69^
ITO/ ZnO/ Cs_3_Bi_2_I_9_/ La_2_NiMnO_6_/ CFTS/ Ag	$26.02$	$42.7$	$0.86$	$75$	^70^
FTO/ TiO_2_/ CsPbI_3_/ Cs_2_SnI_6_/ NiO/ Au	$20.86$	$21.32$	$1.19$	$81.77$	^64^
ITO/ SnO_2_/ MAPbI_3_/ SWCNTs/ NiO_x_/Au	$26.99$	$32.08$	$1$	$84.11$	^71^
Au/ Cu_2_O/ RbGel_3_/ MASnI_3_/ C60/ ITO	$20.19$	$32.7$	$0.91$	$65.49$	^25^
AZO/ TiO_2_/ CsPbI_3_/ RbGel_3_/ Ni	$31.91$	$34.77$	$1.04$	$88.38$	^72^
ITO/ ZnO/ Cs_2_BiAgI_6_/ CIGS/ Spiro-OMeTAD/ Au	$30.1$	$31.89$	$1.11$	$85.01$	^73^
FTO/ n^+^-FASnI_3_/ i-FASnI_3_/ p-FAGeCl_3_/ Au	$30.19$	$31.42$	$1.1$	$87.33$	^74^
FTO/ PC_60_BM/ CsSnl_3_/ CsPbl_3_/ Cu_2_O/Au	$21.71$	$25.56$	$1.13$	$74.92$	^75^
FTO/ PCBM/ CsSnGel_3_/ CsSnI_3_/ Au	$31.31$	$35.31$	$1.01$	$87.63$	^76^
FTO/ SnS_2_/ Cs_2_TiCl_6_/ Cs_2_AgBiI_6_/ Sb_2_S_3_/ C	$32.72$	$26.06$	$1.39$	$90.24$	This work

The highest-performing PSC configuration in this study corresponds to a dual-absorber FTO/ SnS_2_/ Cs_2_TiCl_6_/ Cs_2_AgBiI_6_/ Sb_2_S_3_/ C structure. The optimized design incorporated a $100\ nm$ Cs_2_TiCl_6_ upper absorber with a bulk defect concentration of $1\times {10}^{11} {\ cm}^{-3}$ and a donor atom concentration of $1\times {10}^{14} {\ cm}^{-3}$, atop a $1000\ nm$ Cs_2_AgBiI_6_ lower absorber with density of a bulk defect density of $1\times {10}^{11} {\ cm}^{-3}$ and an acceptor atom concentration of $1\times {10}^{18} {\ cm}^{-3}$. All contacts between the epitaxial layers were assumed to be of superior quality with limited interfacial defect concentrations of $1\times {10}^{10} {\ cm}^{-3}$. The favorable band alignment, which includes a $-0.24\ eV$ cliff at the interface between SnS_2_ and Cs_2_TiCl_6_ layers and a VBO of $-0.18\ eV$ at the interface of Cs_2_AgBiI_6_ with Sb_2_S_3_ HTL, promoted efficient collection of carriers. The optimized device achieves a theoretical PCE of $32.72 \%$, with J_SC_, V_OC_, and FF of $26.06\ mA/{cm}^{2}$, $1.39\ V$, $90.24 \%$, respectively at $300 K$, with parasitic resistances being neglected.

Nevertheless, it is significant to note that the evaluated performance represents an idealized upper limit, achievable only under perfect band alignment conditions and minimal structural defects. Furthermore, the lack of experimental validation of the introduced cesium-based dual-absorber configuration can be considered as a significant limitation of this work. Also, numerical simulations failed to consider several key physical phenomena that influence device performance, including ion migration and Auger non-radiative recombination, as well as operational environmental factors such as humidity, temperature variation and fluctuating light intensity. In particular, the infiltration of oxygen and moisture is known to degrade the chemical stability of perovskite materials, promoting the formation of trap states and shortening carrier lifetimes, which ultimately reduces PCE ^8^. Likewise, temperature fluctuations can influence carrier transport behavior, band alignment, and recombination processes ^60^. Moreover, defect densities in PSCs may change over time due to fabrication defects and environmental stress, which cannot be fully represented in static simulations. Further, bridging the divide between simulation and experimental implementation requires efforts to fabricate high-quality, pure Cs_2_TiCl_6_ and Cs_2_AgBiI_6_ absorber layers with reduced structural defects. In addition, the utilization of advanced interface engineering techniques, coupled with detailed optoelectronic characterization is required to achieve the intended band alignment and mitigate recombination losses.

#### Feasibility of fabrication of proposed device

A comprehensive experimental validation strategy is required to support and verify the device modeling for the proposed device. In the following subsections, a framework integrating material synthesis, device fabrication, photovoltaic performance characterization and long-term stability analysis is introduced to establish a link between theoretical simulations and real-world device performance.

#### Synthesis and processing of double perovskite absorbers for device fabrication

Zhu et al. ^23^ demonstrated that bi-absorber PSCs can be successfully realized via a controlled deposition process, enabling reliable device fabrication. In addition, double perovskite absorbers can be fabricated utilizing solution-based processing, solid-state synthesis, or vapor-assisted deposition techniques, with the choice dependent on target film quality and scalability constraints. Solution processing is particularly favorable due to its low-temperature processing window, cost-effectiveness, and suitability for large-area fabrication. Precise control of precursor stoichiometry, solvent selection, and crystallization dynamics is critical to achieve phase-pure films and prevent the formation of unwanted secondary phases. In addition, annealing under controlled inert or halide-rich conditions can improve crystallinity and assist in defect passivation, in agreement with low defect densities suggested by SCAPS-1D simulations. The crystal structure of Cs_2_TiCl_6_ and Cs_2_AgBiI_6_ can be validated using X-ray diffraction (XRD), whereas scanning electron microscopy (SEM) and atomic force microscopy (AFM) are utilized to examine grain structure, surface roughness, and film continuity. Additionally, elemental composition and uniformity can be evaluated through energy-dispersive X-ray spectroscopy (EDS) and X-ray photoelectron spectroscopy (XPS) ^77^.

Generally, Cs-based double perovskite absorbers can be prepared by spin coating followed by moderate annealing at ${135-285}^{o}C$ ^78^. Further, the proposed PSC adopts a planar structure of FTO/ SnS_2_/ Cs_2_TiCl_6_/ Cs_2_AgBiI_6_/ Sb_2_S_3_/ C, which should be fabricated within inert glovebox environment to prevent degradation caused by oxygen and moisture. Moreover, the consistency of layer deposition and the accuracy of thickness control can be characterized through atomic microscopy (AFM) and profilometry measurements. High interfacial uniformity promotes efficient charge extraction while reducing recombination losses ^33^.

#### Interface engineering and defect passivation

As previously established, the quality of interfacial regions plays a pivotal role in determining both efficiency and operational stability. The SnS_2_/ Cs_2_TiCl_6_ and Cs_2_AgBiI_6_/ Sb_2_S_3_ interfaces can be optimized using surface passivation methods such as controlled plasma treatments, self-assembled monolayers, or ultrathin insulating layers. These strategies effectively decrease interfacial defect densities, limit non-radiative recombination processes, and contribute to achieving the high PCE predicted by device simulations. Furthermore, advanced nanoscale characterization tools, such as Kelvin probe force microscopy (KPFM) and conduction atomic force microscopy (c-AFM), can be utilized to enable detailed analysis of local potential variations and nanoscale charge transport mechanisms ^32^.

#### Evaluation of electrical properties and photovoltaic performance

Photovoltaic performance can be evaluated under standard AM1.5G illumination, yielding key parameters such as PCE, V_OC_, J_SC_, and FF. External and internal quantum efficiency measurements can be conducted to assess wavelength-dependent carrier collection efficiency ^32^. Electrical characterization techniques, including impedance spectroscopy, capacitance–voltage analysis, and time-resolved photoluminescence, will be employed to investigate charge transport resistance, recombination dynamics, carrier lifetime, and defect-induced losses ^77^.

#### Evaluation of device stability and reliability through standardized testing protocols

To evaluate long-term reliability, dual-absorber devices should undergo environmental and operational stability testing. Ambient stability can be assessed by exposing devices to humidity and oxygen, with performance degradation monitored through periodic I-V measurements. Accelerated aging tests involving continuous light exposure and high-temperature conditions are utilized to simulate extended operating conditions. Additionally, thermal cycling and mechanical stress testing should be conducted to evaluate device robustness, particularly for flexible device configurations. Additionally, the development of durable encapsulation schemes is needed to preserve the operational stability and environmental resilience of the PSC ^77^.

Ultimately, the present theoretical study provides practical insights that can be utilized for future experimental realization by highlighting the critical roles of electrode and CTL material selection, thickness optimization of absorbers, control of doping density, and interfacial engineering in altering the performance of PSCs. Furthermore, the consideration of practical influences including series and shunt resistances, operational temperature as well as fabrication tolerance analysis establishes a more realistic framework for assessing the robustness of the proposed device. In general, this theoretical study serves to guide experimental realization by minimizing iterative fabrication trails and accelerating the advancement of high-performance SCs.

## Conclusion

The results of the presented study demonstrate that the performance enhancement of the proposed Cs_2_TiCl_6_/ Cs_2_AgBiI_6_ dual-absorber lead-free PSC can primarily be achieved by the optimization of absorber properties and interfacial characteristics, in addition to the employment of suitable CTLs. In particular, reducing bulk and interface defect densities plays a critical role in suppressing recombination, thereby improving carrier lifetime and escalating PCE. In addition, careful tuning of absorber thickness and doping concentration enhances light absorption and charge separation and carrier transport, leading to improved overall performance. Further, the present analysis suggests utilizing SnS_2_ and Sb_2_S_3_ as ETL and HTL, respectively, for their perfect band alignment with upper Cs_2_TiCl_6_ and lower Cs_2_AgBiI_6_ absorbers, which facilitates the separation and collection of carriers. The optimum device introduced in this study, with FTO/ SnS_2_/ Cs_2_TiCl_6_/ Cs_2_AgBiI_6_/ Sb_2_S_3_/ C structure, theoretically achieves a remarkable PCE of $32.72 \%$, in addition to elevating J_SC_, V_OC_, and FF to $26.06\ mA/{cm}^{2}$, $1.39\ V$, $90.24 \%$, respectively. The achieved PCE by the optimized Cs_2_TiCl_6_/ Cs_2_AgBiI_6_ proposed device surpasses those of Cs_2_AgBiI_6_ and Cs_2_TiCl_6_ single absorber PSCs by $4.88 \%$ and $21.91 \%$, respectively. Furthermore, the integration of ML provides a powerful tool for identifying nonlinear relationships between device parameters and performance metrics. In this regard, XGB achieved high estimation accuracy with values of ${R}^{2}$ equal $0.9982$, $0.9981$, $0.9997$, and $0.9937$ for predicting the values of PCE, V_OC_, J_SC_, and FF of the proposed device, respectively. The use of SHAP-based interpretability enabled a deeper understanding of the relative influence of key parameters, offering physical insights that complemented simulation results.

## Supplementary Information

Supplementary Information 1.

Supplementary Information 2.

## Data Availability

All data that supports the findings of this study are included within the article and its Supplementary Information. The corresponding ML codes are available from the corresponding author upon reasonable request.
