# Supplementary material for: Machine learning prediction of dual absorber lead-free perovskite solar cells for boosting PCE
Source: Sci Rep. 2026 May 24;16:16027. doi: 10.1038/s41598-026-51970-5 (PMC13199426; doi:10.1038/s41598-026-51970-5)
Supplement: Supplementary file 1 — Supplementary Information 1. [file 41598_2026_51970_MOESM1_ESM.docx]

**Supplementary Information**

**Machine Learning Prediction of Dual Absorber Lead-Free Perovskite Solar Cells for Boosting PCE**

**Table S1** Parameters of the different HTLs investigated in the study

| **Parameters** | $\boldsymbol{\varepsilon}_{\boldsymbol{r}}$ | $\boldsymbol{E}_{\boldsymbol{g}}$  ($\boldsymbol{eV}$) | $\boldsymbol{\chi}$  ($\boldsymbol{eV}$) | $\boldsymbol{N}_{\boldsymbol{c}}$  ($\boldsymbol{cm}^{\boldsymbol{-3}}$) | $\boldsymbol{N}_{\boldsymbol{v}}$  ($\boldsymbol{cm}^{\boldsymbol{-3}}$) | $\boldsymbol{\mu}_{\boldsymbol{n}}$ / $\boldsymbol{\mu}_{\boldsymbol{p}}$  (${\boldsymbol{cm}^{\boldsymbol{2}}}/\boldsymbol{Vs})$ | $\boldsymbol{N}_{\boldsymbol{A}}$  ($\boldsymbol{cm}^{\boldsymbol{-3}}$) | $\boldsymbol{N}_{\boldsymbol{t}}$  ($\boldsymbol{cm}^{\boldsymbol{-3}}$) | **Ref.** |
| --- | --- | --- | --- | --- | --- | --- | --- | --- | --- |
| $\boldsymbol{Spiro-}$  $\boldsymbol{OMeTAD}$ | $3$ | $3$ | $2.2$ | $2.2\times{10}^{18}$ | $1.8\times{10}^{19}$ | $2.1\times{10}^{-3}$  $/ 2.16\times{10}^{-3}$ | $1\times{10}^{18}$ | $1\times{10}^{15}$ | [1] |
| $\boldsymbol{Cu}_{\boldsymbol{2}}\boldsymbol{O}$ | $7.5$ | $2.2$ | $3.4$ | $2\times{10}^{19}$ | $1\times{10}^{19}$ | $200/8600$ | $1\times{10}^{18}$ | $1\times{10}^{15}$ | [1] |
| $\boldsymbol{P}\boldsymbol{3}\boldsymbol{HT}$ | $3$ | $1.7$ | $3.5$ | $2\times{10}^{18}$ | $2\times{10}^{19}$ | $1.8\times{10}^{-3}$  $/1.86\times{10}^{-2}$ | $1\times{10}^{18}$ | $1\times{10}^{14}$ | [2] |
| $\boldsymbol{PEDOT:PSS}$ | $3$ | $1.6$ | $3.4$ | $2.2\times{10}^{18}$ | $1.8\times{10}^{19}$ | $4.5\times{10}^{-2}$  $/4.5\times{10}^{-2}$ | $1\times{10}^{18}$ | $1\times{10}^{15}$ | [1] |
| $\boldsymbol{CuI}$ | $6.5$ | $3.1$ | $2.1$ | $2.8\times{10}^{19}$ | $1\times{10}^{19}$ | $100/ 43.9$ | $1\times{10}^{18}$ | $1\times{10}^{15}$ | [1] |
| $\boldsymbol{CuO}$ | $18.1$ | $1.51$ | $4.07$ | $2.2\times{10}^{19}$ | $5.5\times{10}^{20}$ | $100/0.1$ | $1\times{10}^{18}$ | $1\times{10}^{15}$ | [1] |
| $\boldsymbol{CFTS}$ | $9$ | $1.3$ | $3.3$ | $2.2\times{10}^{18}$ | $1.8\times{10}^{19}$ | $21.98/21.98$ | $1\times{10}^{18}$ | $1\times{10}^{15}$ | [1] |
| $\boldsymbol{CBTS}$ | $5.4$ | $1.9$ | $3.6$ | $2.2\times{10}^{18}$ | $1.8\times{10}^{19}$ | $30/10$ | $1\times{10}^{18}$ | $1\times{10}^{15}$ | [1] |
| $\boldsymbol{PTAA}$ | $9$ | $2.96$ | $2.3$ | $2\times{10}^{21}$ | $2\times{10}^{21}$ | $1/40$ | $1\times{10}^{18}$ | $1\times{10}^{15}$ | [3] |
| $\boldsymbol{Mo}\boldsymbol{O}_{\boldsymbol{3}}$ | $18$ | $3$ | $2.3$ | $1\times{10}^{19}$ | $2.2\times{10}^{18}$ | $210/210$ | $1\times{10}^{18}$ | $1\times{10}^{15}$ | [3] |
| $\boldsymbol{Mo}\boldsymbol{S}_{\boldsymbol{2}}$ | $3$ | $1.29$ | $4.2$ | $2.2\times{10}^{18}$ | $1.9\times{10}^{19}$ | $100/150$ | $1\times{10}^{17}$ | $1\times{10}^{14}$ | [4] |
| $\boldsymbol{Cu}_{\boldsymbol{2}}\boldsymbol{Te}$ | $10$ | $1.18$ | $4.2$ | $7.8\times{10}^{17}$ | $1.6\times{10}^{19}$ | $500/100$ | $1\times{10}^{21}$ | $1\times{10}^{14}$ | [4] |
| $\boldsymbol{nPB}$ | $3$ | $2.4$ | $3$ | $2.7\times{10}^{19}$ | $3.5\times{10}^{20}$ | $6.1/6.1$ | $1\times{10}^{18}$ | $1\times{10}^{15}$ | [5] |
| $\boldsymbol{Sb}_{\boldsymbol{2}}\boldsymbol{S}_{\boldsymbol{3}}$ | $7.08$ | $1.62$ | $3.7$ | $2\times{10}^{19}$ | $1\times{10}^{19}$ | $9.8/10$ | $1\times{10}^{18}$ | $1\times{10}^{15}$ | [5] |

**Table S2** Parameters of the different ETLs investigated in the study

| **Parameters** | $\boldsymbol{\varepsilon}_{\boldsymbol{r}}$ | $\boldsymbol{E}_{\boldsymbol{g}}$  ($\boldsymbol{eV}$) | $\boldsymbol{\chi}$  ($\boldsymbol{eV}$) | $\boldsymbol{N}_{\boldsymbol{c}}$  ($\boldsymbol{cm}^{\boldsymbol{-3}}$) | $\boldsymbol{N}_{\boldsymbol{v}}$  ($\boldsymbol{cm}^{\boldsymbol{-3}}$) | $\boldsymbol{\mu}_{\boldsymbol{n}}$ / $\boldsymbol{\mu}_{\boldsymbol{p}}$  (${\boldsymbol{cm}^{\boldsymbol{2}}}/\boldsymbol{Vs})$ | $\boldsymbol{N}_{\boldsymbol{D}}$  ($\boldsymbol{cm}^{\boldsymbol{-3}}$) | $\boldsymbol{N}_{\boldsymbol{t}}$  ($\boldsymbol{cm}^{\boldsymbol{-3}}$) | **Ref.** |
| --- | --- | --- | --- | --- | --- | --- | --- | --- | --- |
| $\boldsymbol{TiO}_{\boldsymbol{2}}$ | $9$ | $3.2$ | $4$ | $2\times{10}^{18}$ | $1.8\times{10}^{19}$ | $20/10$ | $9\times{10}^{16}$ | $1\times{10}^{15}$ | [1] |
| $\boldsymbol{PCBM}$ | $3.9$ | $2$ | $3.9$ | $2.5\times{10}^{21}$ | $2.5\times{10}^{21}$ | $0.2/0.2$ | $2.93\times{10}^{17}$ | $1\times{10}^{15}$ | [1] |
| $\boldsymbol{ZnO}$ | $9$ | $3.3$ | $4$ | $3.7\times{10}^{18}$ | $1.8\times{10}^{19}$ | $100/25$ | $1\times{10}^{18}$ | $1\times{10}^{15}$ | [1] |
| $\boldsymbol{C}\boldsymbol{60}$ | $4.2$ | $1.7$ | $3.9$ | $8\times{10}^{19}$ | $8\times{10}^{19}$ | $8\times{10}^{-2}$  $/3.5\times{10}^{-3}$ | $1\times{10}^{17}$ | $1\times{10}^{15}$ | [1] |
| $\boldsymbol{IGZO}$ | $10$ | $3.05$ | $4.16$ | $5\times{10}^{18}$ | $5\times{10}^{18}$ | $15/0.1$ | $1\times{10}^{17}$ | $1\times{10}^{15}$ | [1] |
| $\boldsymbol{SnO}_{\boldsymbol{2}}$ | $9$ | $3.6$ | $4$ | $2.2\times{10}^{18}$ | $1.8\times{10}^{19}$ | $100/25$ | $1\times{10}^{17}$ | $1\times{10}^{15}$ | [1] |
| $\boldsymbol{WS}_{\boldsymbol{2}}$ | $13.6$ | $1.8$ | $3.95$ | $1\times{10}^{18}$ | $2.4\times{10}^{19}$ | $100/100$ | $1\times{10}^{18}$ | $1\times{10}^{15}$ | [1] |
| $\boldsymbol{SnS}_{\boldsymbol{2}}$ | $17.7$ | $1.85$ | $4.26$ | $7.32\times{10}^{18}$ | $1\times{10}^{19}$ | $50/25$ | $9.85\times{10}^{19}$ | $1\times{10}^{14}$ | [6] |
| $\boldsymbol{CdS}$ | $10$ | $2.4$ | $4.5$ | $2.2\times{10}^{18}$ | $1.8\times{10}^{19}$ | $350/25$ | $1\times{10}^{17}$ | $1\times{10}^{14}$ | [7] |
| $\boldsymbol{ZnSe}$ | $8.6$ | $2.81$ | $4.09$ | $2.2\times{10}^{18}$ | $1.8\times{10}^{18}$ | $4\times{10}^{2}$  $/1.1\times{10}^{2}$ | $1\times{10}^{18}$ | $1\times{10}^{15}$ | [6] |
| $\boldsymbol{PC}_{\boldsymbol{60}}\boldsymbol{BM}$ | $4$ | $1.8$ | $4.2$ | $1\times{10}^{21}$ | $2\times{10}^{20}$ | $0.1/0.1$ | $1\times{10}^{17}$ | $1\times{10}^{15}$ | [6] |
| $\boldsymbol{LBSO}$ | $22$ | $3.12$ | $4.4$ | $1.8\times{10}^{20}$ | $1.8\times{10}^{20}$ | $0.69/0.69$ | $2\times{10}^{21}$ | $1\times{10}^{14}$ | [3] |
| $\boldsymbol{Nb}_{\boldsymbol{2}}\boldsymbol{O}_{\boldsymbol{5}}$ | $45$ | $3.46$ | $4.33$ | $1\times{10}^{19}$ | $2.2\times{10}^{18}$ | $20.73/20.73$ | $1\times{10}^{15}$ | $1\times{10}^{15}$ | [3] |
| $\boldsymbol{CdZnS}$ | $9.12$ | $3.2$ | $4.2$ | $1.5\times{10}^{18}$ | $1.8\times{10}^{18}$ | $250/40$ | $1\times{10}^{16}$ | $1\times{10}^{14}$ | [3] |

**Table S3** Numerical formulas utilized in the study

| **Description** | **Formula** | **Ref.** |
| --- | --- | --- |
| Poisson's equation | $\frac{\partial^{2}\varphi(x)}{\partial x^{2}}=-\frac{\partial E}{\partial x}=-\frac{\rho}{\varepsilon_{s}}=-\frac{q}{\varepsilon_{s}}\left( p\left( x \right)-n\left( x \right)+N_{D}\left( x \right)-N_{A}\left( x \right)\pm N_{t}\left( x \right) \right)$ | [7] |
| Continuity equations | $\frac{\partial n}{\partial t}=\frac{1}{q}\frac{\partial J_{n}}{\partial x}+G_{n}-R_{n}$  $\frac{\partial p}{\partial t}=-\frac{1}{q}\frac{\partial J_{p}}{\partial x}+G_{p}-R_{p}$ | [8] |
| Transport equations | $J_{n}=q(\mu_{n}nE+D_{n}\frac{\partial n}{\partial x})$  $J_{p}=q(\mu_{p}pE+D_{p}\frac{\partial p}{\partial x})$ | [9] |
| Absorption model | $\alpha\left( \lambda\right)=(A+\frac{B}{hv})\sqrt{hv-E_{g}}$ | [10] |
| Mean absolute error | $MAE=\frac{1}{N}\sum_{i=1}^{N} \left( y_{i}-\hat{y}_{i} \right)$ | [11] |
| Mean squared error | $MSE=\frac{1}{N}\sum_{i=1}^{N} \left( y_{i}-\hat{y}_{i} \right)^{2}$ | [11] |
| Root mean square error | $RMSE=\sqrt{MSE}$ | [11] |
| Correlation coefficient | $R^{2}=1-\frac{\sum_{i=1}^{N} \left( y_{i}-\hat{y}_{i} \right)^{2}}{\sum_{i=1}^{N} \left( y_{i}-\bar{y} \right)^{2}}$ | [11] |

**Table S4** Physical parameters employed in utilized formulas and their description

| **Parameter** | **Description** |
| --- | --- |
| $\varphi$ | Electrostatic potential |
| $E$ | Electric field |
| $q$ | Electron charge |
| $\varepsilon_{s}$ | Material permittivity |
| $p$ | Hole density |
| $n$ | Electron density |
| $N_{t}$ | Defect density |
| $G_{p}$ | Hole generation rate |
| $R_{p}$ | Hole recombination rate |
| $G_{n}$ | Electron generation rate |
| $R_{n}$ | Electron recombination rate |
| $J_{n}$ | Electron current density |
| $J_{p}$ | Hole current density |
| $\mu_{n}$ | Electron mobility |
| $\mu_{p}$ | Hole mobility |
| $E_{g}$ | Energy bandgap |
| $h$ | Plank's constant |
| $v$ | photons frequency |
| $N$ | number of samples |
| $y_{i}$ | $i^{th}$ predicted value |
| $\hat{y}_{i}$ | $i^{th}$ actual value |
| $\bar{y}$ | average value |

**Table S5** Initial data for reproduction the fabricated structure reported by Lu et al. [12]

| **Parameters** | $\boldsymbol{Sn}\boldsymbol{O}_{\boldsymbol{2}}$ | $\boldsymbol{CH}_{\boldsymbol{3}}\boldsymbol{NH}_{\boldsymbol{3}}\boldsymbol{Pb}\boldsymbol{I}_{\boldsymbol{3}}$ | $\boldsymbol{P}\boldsymbol{3}\boldsymbol{HT}$ | $\boldsymbol{Ni}\boldsymbol{O}_{\boldsymbol{x}}$ |
| --- | --- | --- | --- | --- |
| $\boldsymbol{thickness (nm)}$ | $50$ | $800$ | $30$ | $44$ |
| $\boldsymbol{E}_{\boldsymbol{g}}$ ($\boldsymbol{eV}$) | $3.6$ | $1.55$ | $1.7$ | $3.7$ |
| $\boldsymbol{\chi}$ ($\boldsymbol{eV}$) | $4$ | $3.93$ | $3.5$ | $1.7$ |
| $\boldsymbol{\varepsilon}_{\boldsymbol{r}}$ | $9$ | $6.5$ | $3$ | $10.7$ |
| $\boldsymbol{N}_{\boldsymbol{c}}$ ($\boldsymbol{cm}^{\boldsymbol{-3}}$) | $2.2\times{10}^{18}$ | $2.75\times{10}^{18}$ | $2\times{10}^{18}$ | $2.2\times{10}^{19}$ |
| $\boldsymbol{N}_{\boldsymbol{v}}$ ($\boldsymbol{cm}^{\boldsymbol{-3}}$) | $1.8\times{10}^{19}$ | $3.9\times{10}^{18}$ | $2\times{10}^{19}$ | $1.8\times{10}^{19}$ |
| $\boldsymbol{\mu}_{\boldsymbol{n}}$ / $\boldsymbol{\mu}_{\boldsymbol{p}}$  (${\boldsymbol{cm}^{\boldsymbol{2}}}/\boldsymbol{Vs})$ | $100/25$ | $50/50$ | $1.8\times{10}^{-3}$/ $1.86\times{10}^{-2}$ | $12/25$ |
| $\boldsymbol{N}_{\boldsymbol{D}}$ ($\boldsymbol{cm}^{\boldsymbol{-3}}$) | $1\times{10}^{17}$ | - | - | - |
| $\boldsymbol{N}_{\boldsymbol{A}}$ ($\boldsymbol{cm}^{\boldsymbol{-3}}$) | - | $5\times{10}^{13}$ | $1\times{10}^{18}$ | $1\times{10}^{15}$ |
| $\boldsymbol{N}_{\boldsymbol{t}}$ ($\boldsymbol{cm}^{\boldsymbol{-3}}$) | $1\times{10}^{15}$ | $3\times{10}^{14}$ | $1\times{10}^{14}$ | $1\times{10}^{14}$ |
| $\boldsymbol{Ref.}$ | [1] | [13–15] | [2] | [16,17] |

**Table S6** Electrical parameters of the simulated PSC compared to those of the fabricated structure reported by Lu et al. [12]

|  | $\boldsymbol{J}_{\boldsymbol{SC}}\boldsymbol{(mA/}\boldsymbol{cm}^{\boldsymbol{2}}\boldsymbol{)}$ | $\boldsymbol{V}_{\boldsymbol{OC}}\boldsymbol{(V)}$ | $\boldsymbol{PCE (\%)}$ | $\boldsymbol{FF (\%)}$ |
| --- | --- | --- | --- | --- |
| $\boldsymbol{Simulated Device}$ | $22.90$ | $1.15$ | $20.80$ | $78.80$ |
| $\boldsymbol{Ref. Fabricated}$  $\boldsymbol{Device}$ | $22.82$ | $1.11$ | $20.88$ | $82.48$ |

**Table S7** Statistical analysis of the generated dataset

| $\boldsymbol{Parameter}$ | $\boldsymbol{Count}$ | $\boldsymbol{Mean}$ | $\boldsymbol{Std.}$ | $\boldsymbol{Min}$ | $\boldsymbol{Max}$ |
| --- | --- | --- | --- | --- | --- |
| Thickness of Cs_2_TiCl_6_ layer | $2187$ | $0.533$ | $0.368$ | $0.1$ | $1$ |
| Thickness of Cs_2_AgBil_6_ layer | $2187$ | $0.633$ | $0.449$ | $0.1$ | $1.2$ |
| Defect density of Cs_2_TiCl_6_ layer | $2187$ | $3.37\times{10}^{15}$ | $4.69\times{10}^{15}$ | $1\times{10}^{11}$ | $1\times{10}^{16}$ |
| Defect density of Cs_2_AgBil_6_ layer | $2187$ | $3.37\times{10}^{15}$ | $4.69\times{10}^{15}$ | $1\times{10}^{11}$ | $1\times{10}^{16}$ |
| Doping density of Cs_2_TiCl_6_ layer | $2187$ | $3.37\times{10}^{18}$ | $4.69\times{10}^{18}$ | $1\times{10}^{14}$ | $1\times{10}^{19}$ |
| Doping density of Cs_2_AgBil_6_ layer | $2187$ | $3.37\times{10}^{18}$ | $4.69\times{10}^{18}$ | $1\times{10}^{14}$ | $1\times{10}^{19}$ |
| Interface Defect density between the two absorbers | $2187$ | $3.37\times{10}^{15}$ | $4.69\times{10}^{15}$ | $1\times{10}^{11}$ | $1\times{10}^{16}$ |


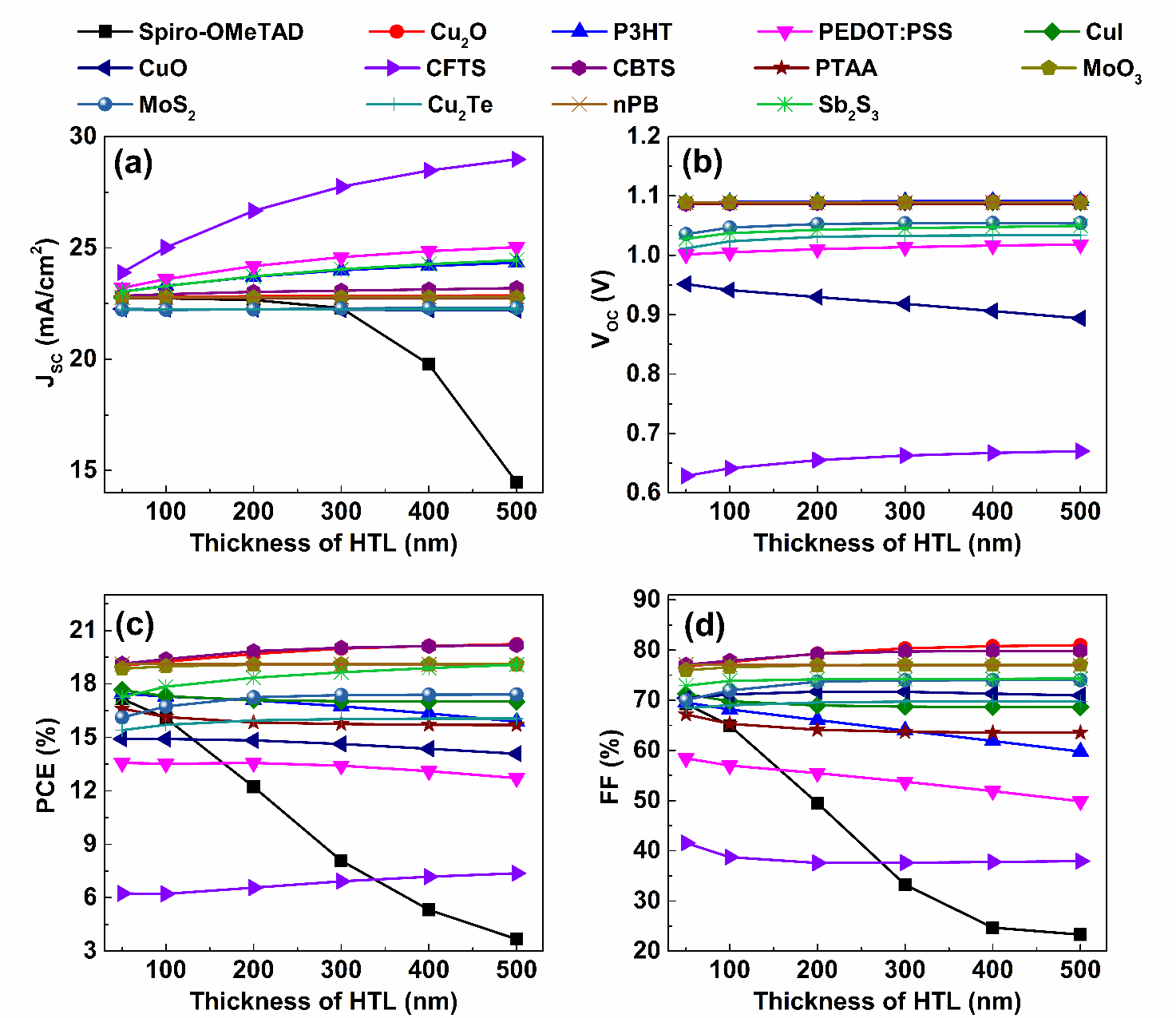


**Fig. S1** In evaluating the proposed dual- absorber PSC with different HTLs, the influence of HTL thickness on (a) J_SC_ (b)V_OC_ (c) PCE (d) FF


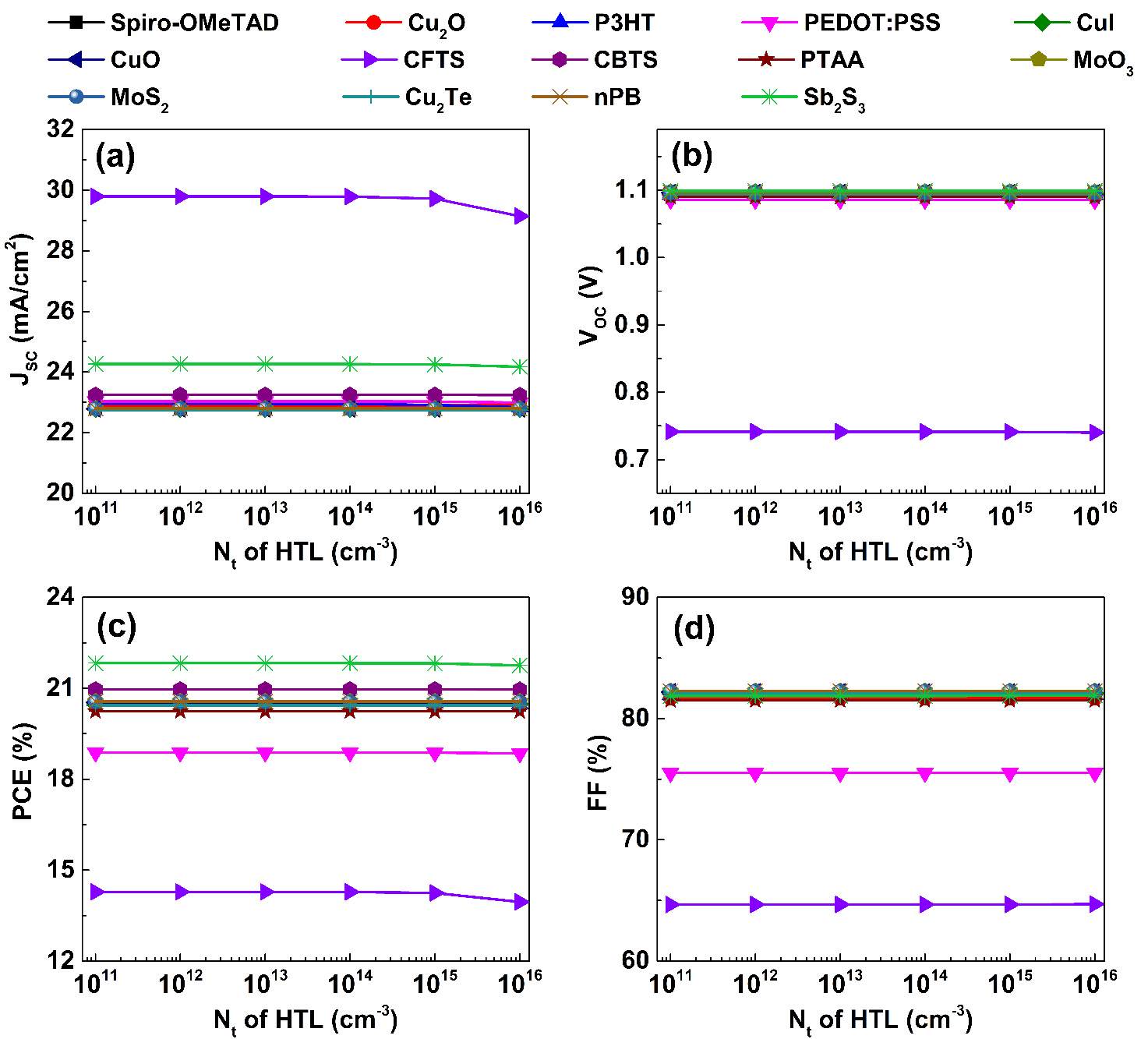


**Fig. S2** In evaluating the proposed dual- absorber PSC with different HTLs, the influence of HTL defect density on (a) J_SC_ (b)V_OC_ (c) PCE (d) FF


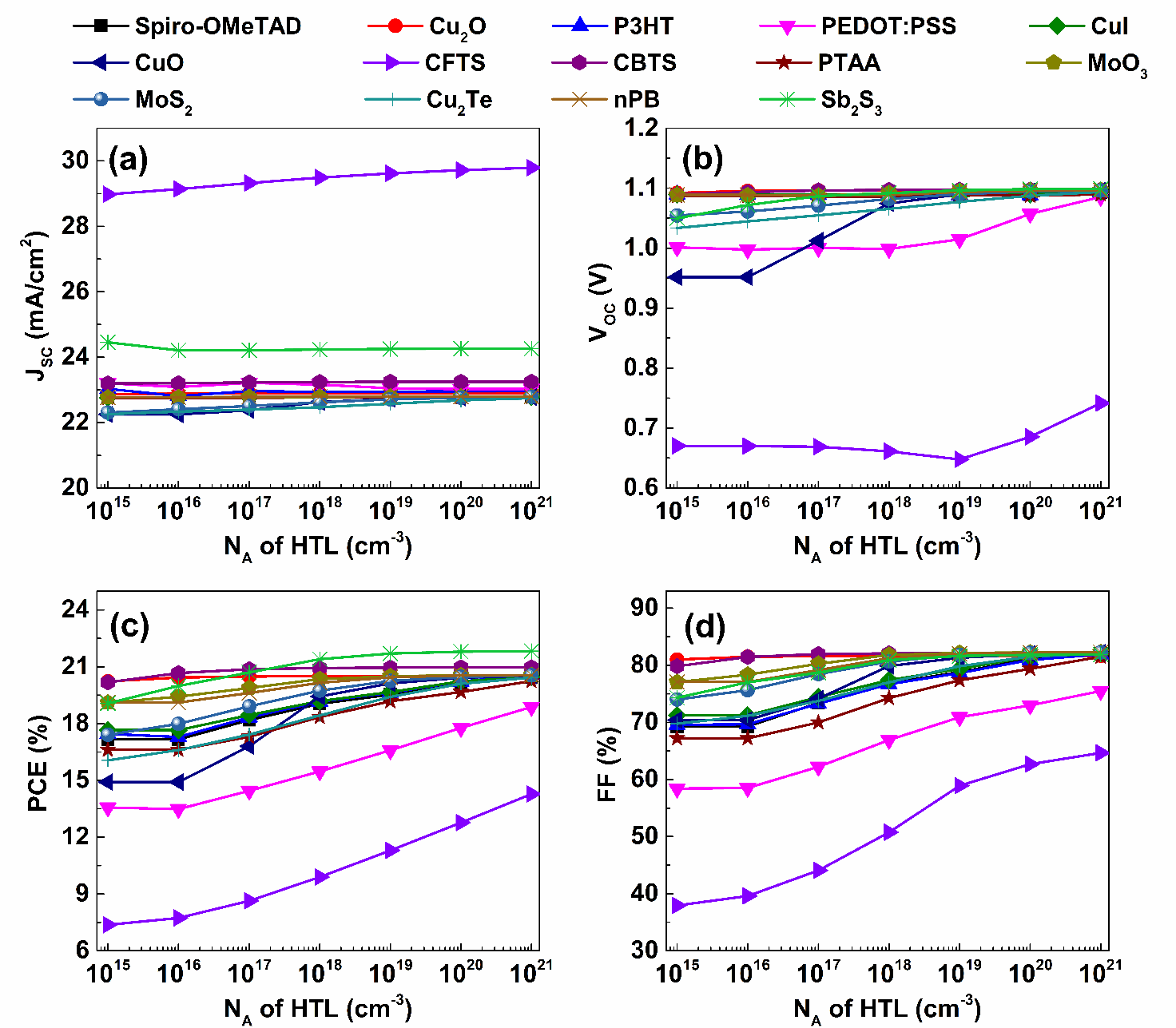


**Fig. S 3** In evaluating the proposed dual- absorber PSC with different HTLs, the influence of HTL acceptor doping density on (a) J_SC_ (b)V_OC_ (c) PCE (d) FF


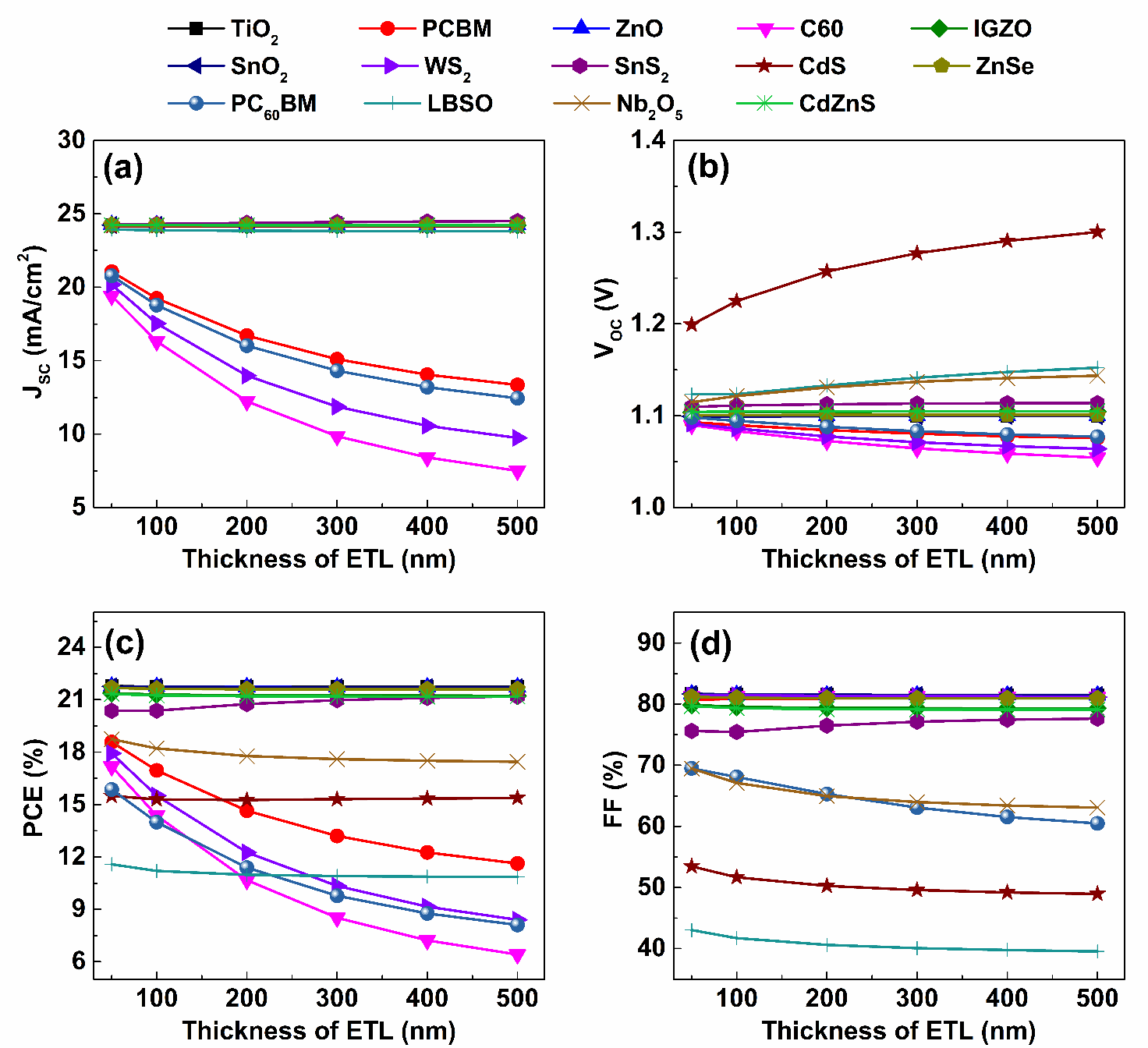


**Fig. S4** In evaluating the proposed dual- absorber PSC with different ETLs, the influence of ETL thickness on (a) J_SC_ (b)V_OC_ (c) PCE (d) FF


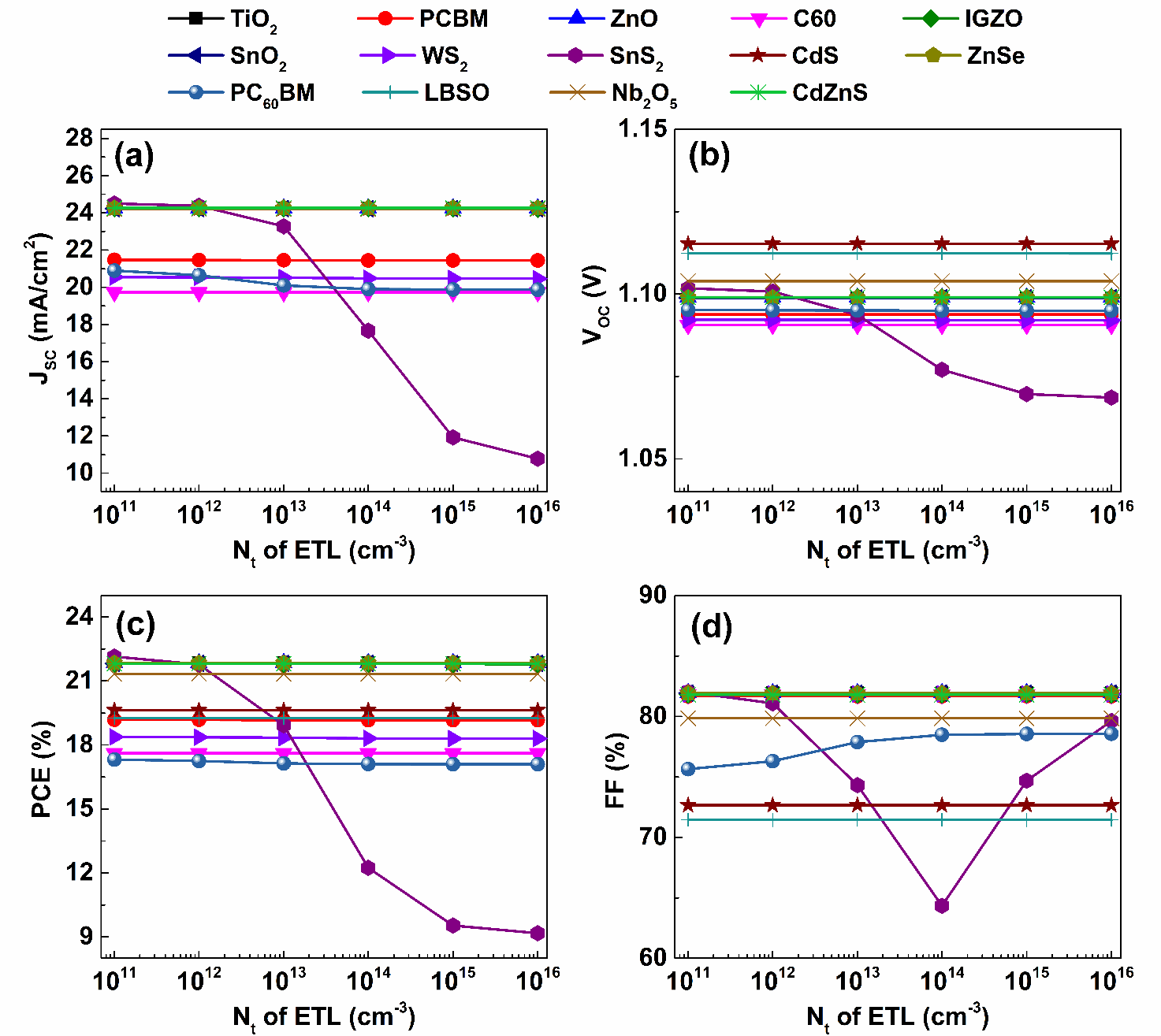


**Fig. S5** In evaluating the proposed dual- absorber PSC with different ETLs, the influence of ETL defect density on (a) J_SC_ (b)V_OC_ (c) PCE (d) FF


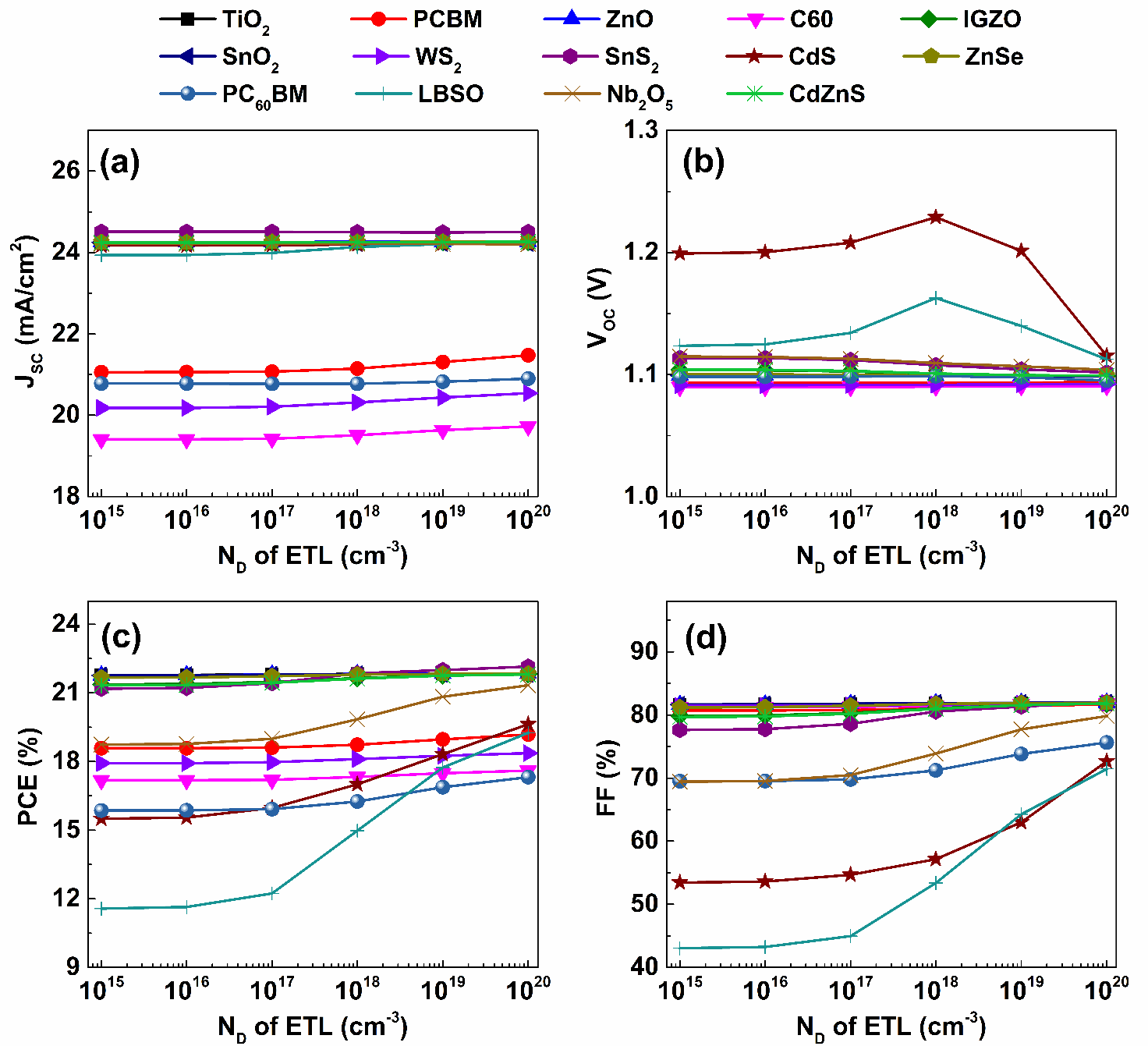


**Fig. S6** In evaluating the proposed dual- absorber PSC with different ETLs, the influence of ETL donor doping density on (a) J_SC_ (b)V_OC_ (c) PCE (d) FF


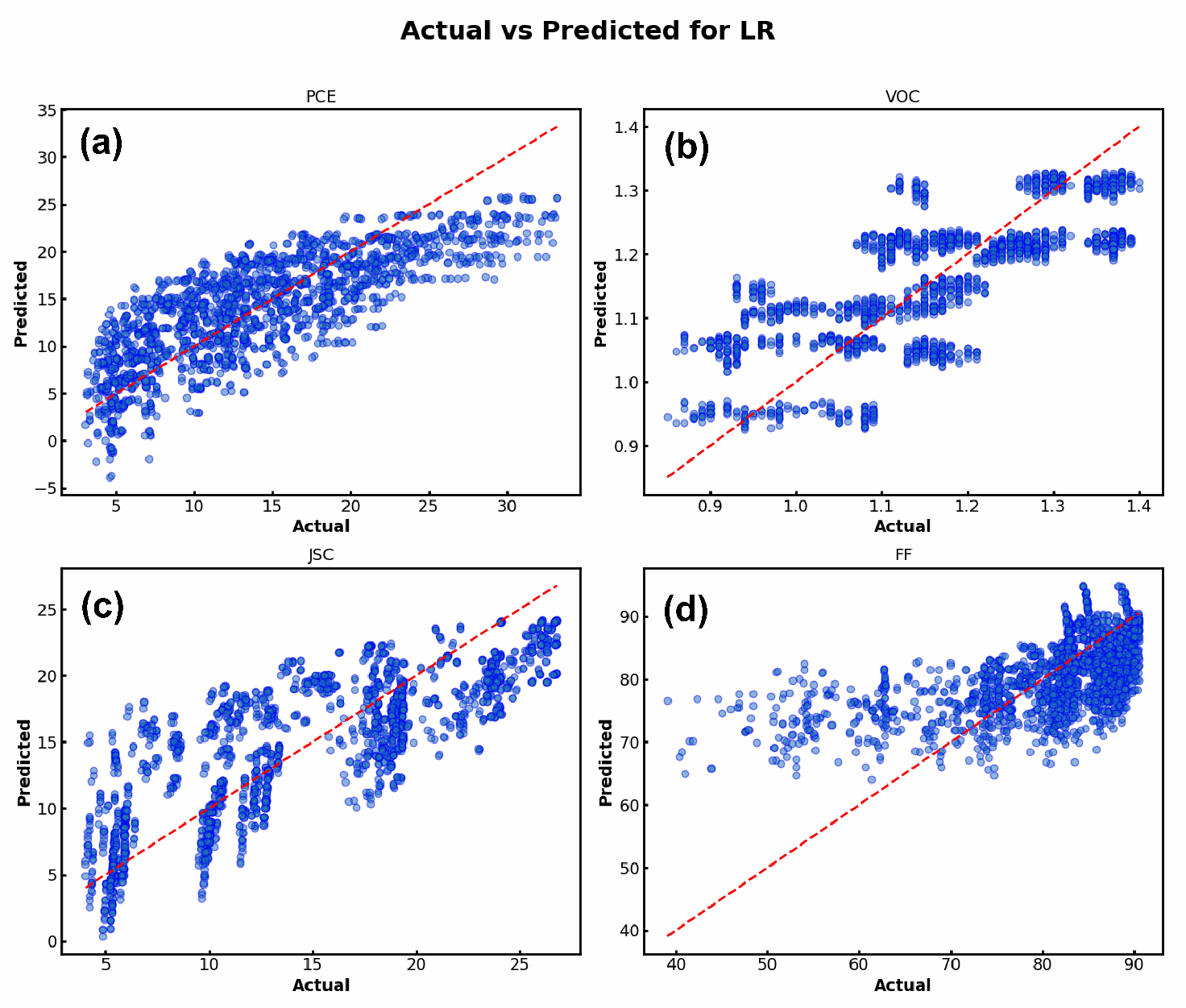


**Fig. S7** Comparison of predicted and actual values of **(a)** PCE **(b)** V_OC_ **(c)** J_SC_ **(d)** FF using LR algorithm


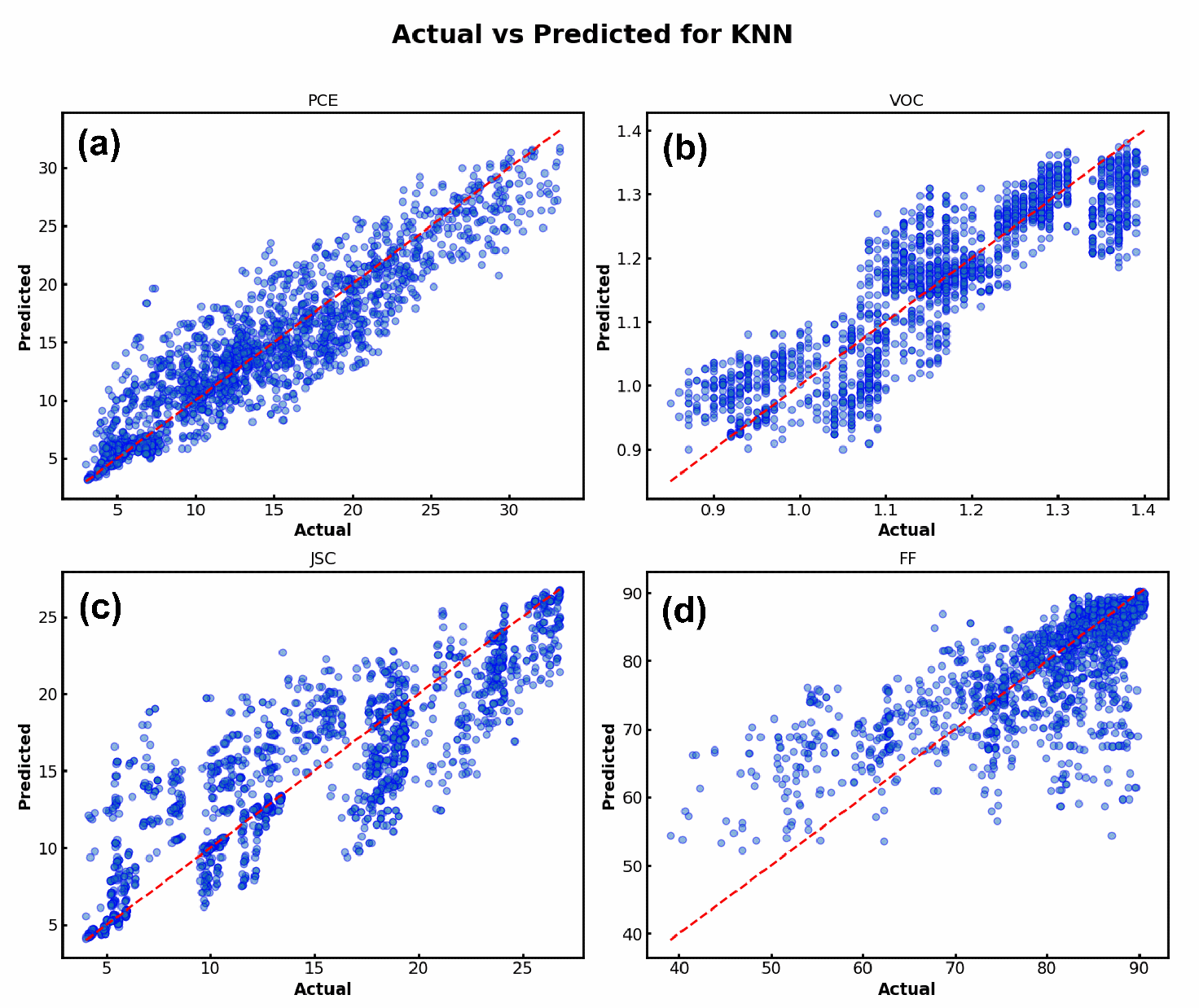


**Fig. S8** Comparison of predicted and actual values of **(a)** PCE **(b)** V_OC_ **(c)** J_SC_ **(d)** FF using KNN algorithm


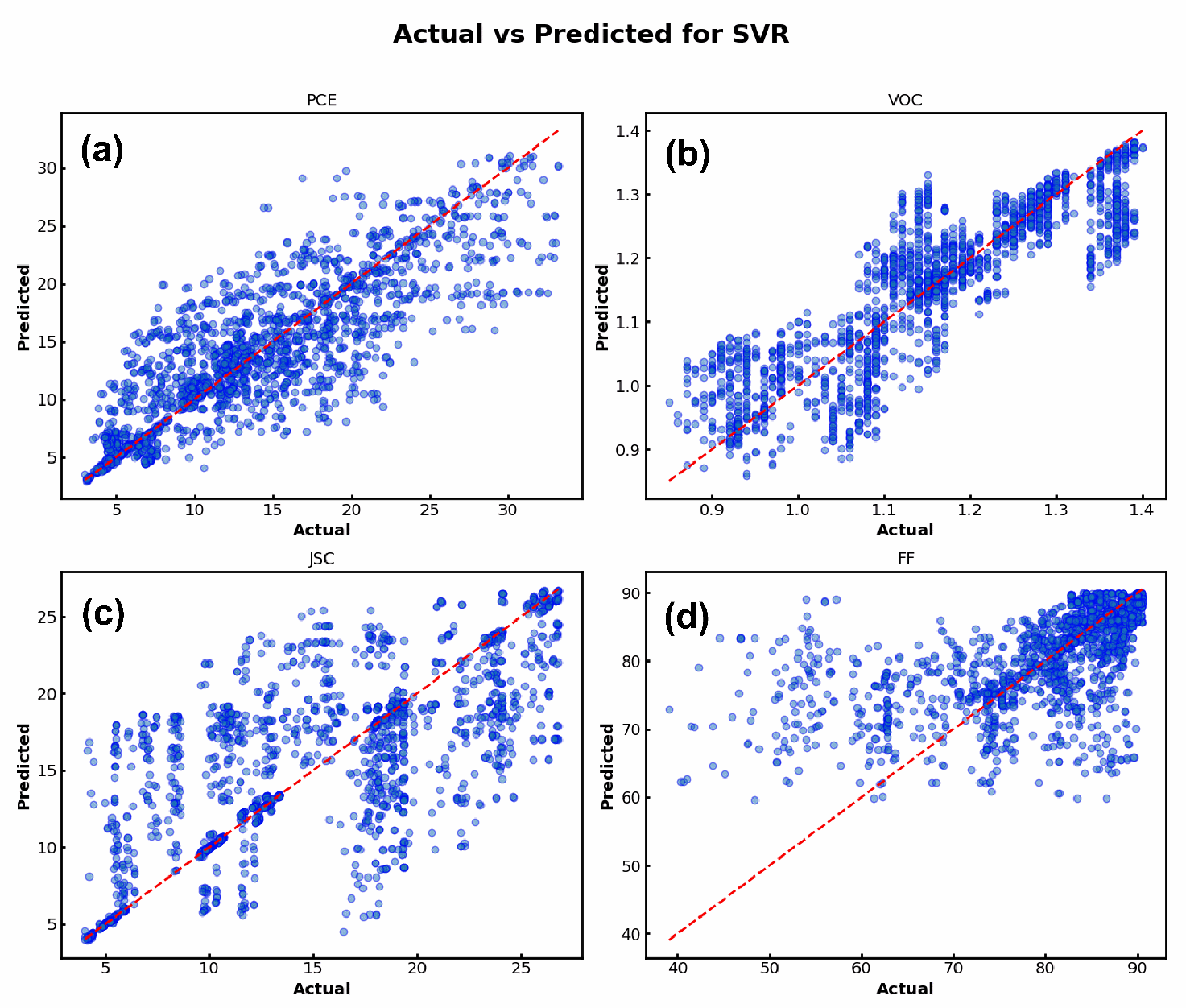


**Fig. S9** Comparison of predicted and actual values of **(a)** PCE **(b)** V_OC_ **(c)** J_SC_ **(d)** FF using SVR algorithm


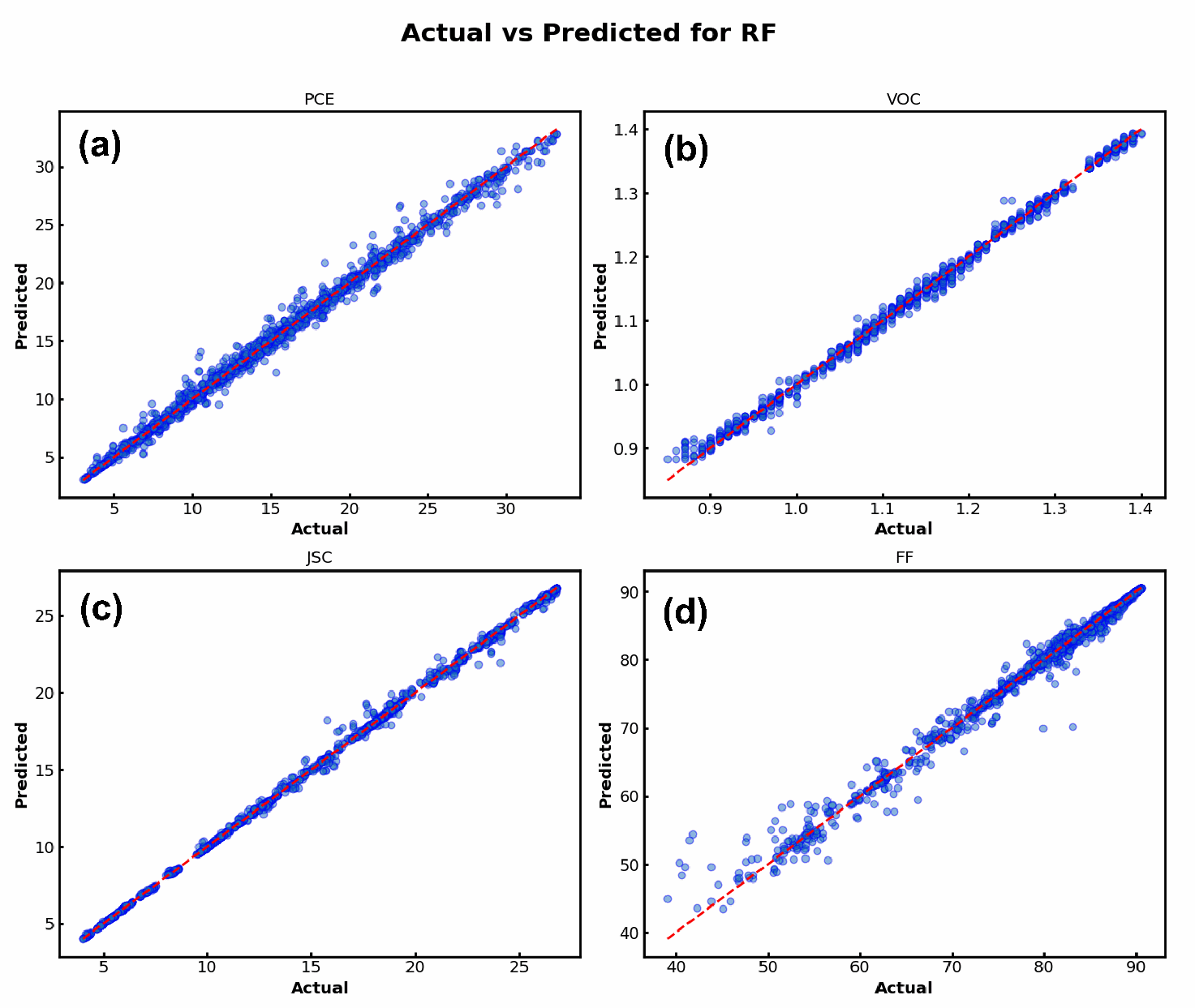


**Fig. S10** Comparison of predicted and actual values of **(a)** PCE **(b)** V_OC_ **(c)** J_SC_ **(d)** FF using RF algorithm


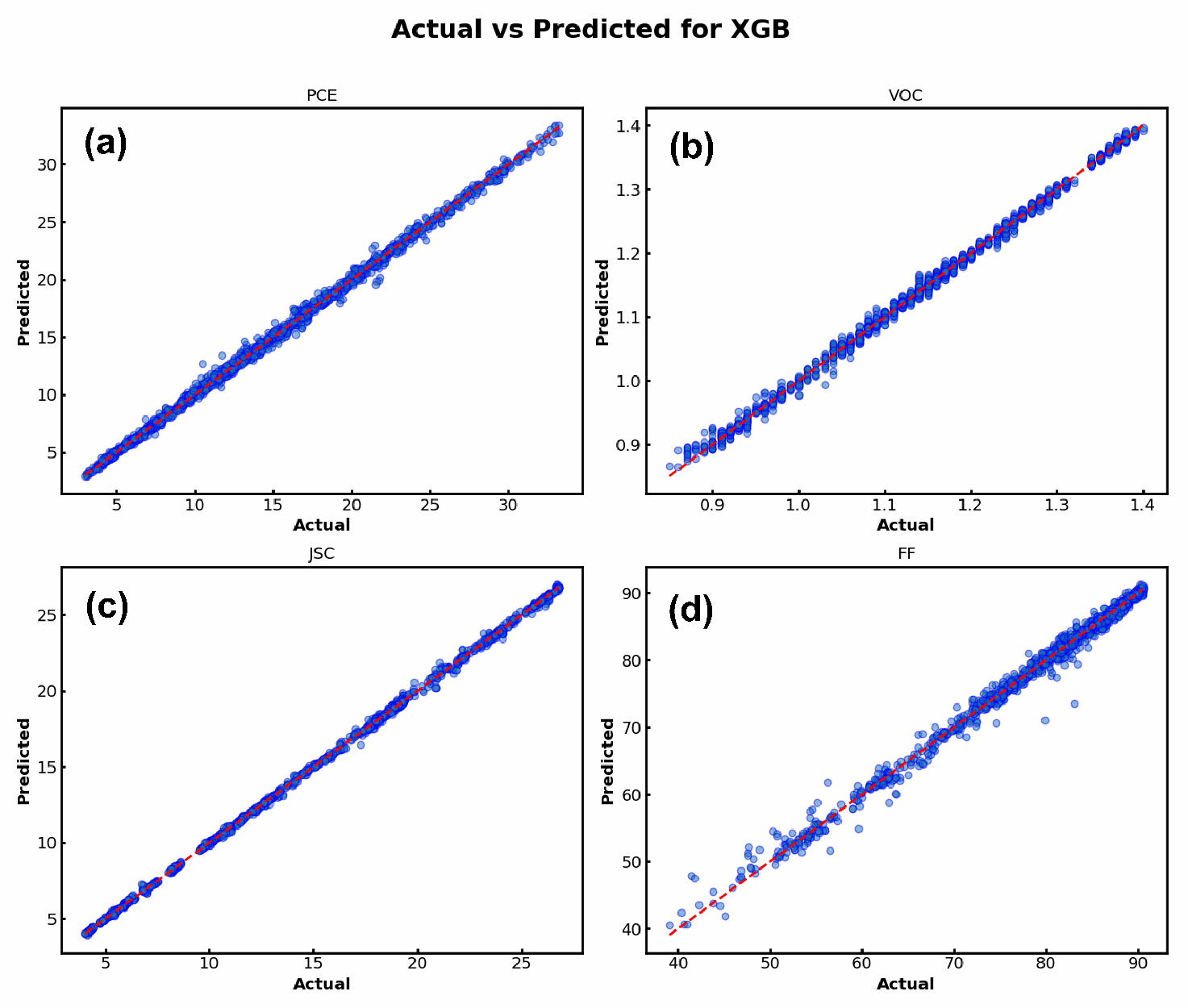


**Fig. S 11** Comparison of predicted and actual values of **(a)** PCE **(b)** V_OC_ **(c)** J_SC_ **(d)** FF using XGB algorithm


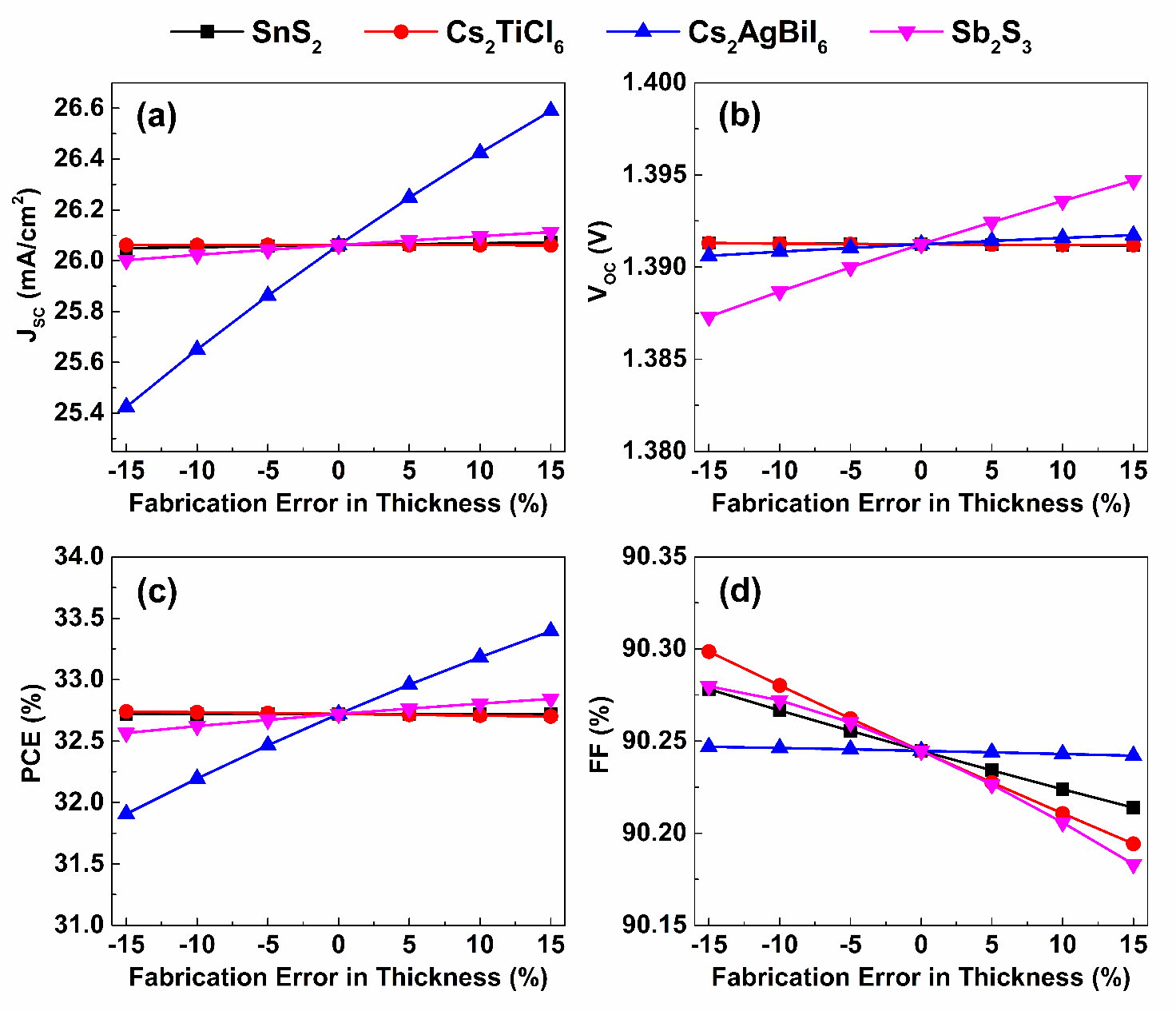


**Fig. S12** Variation of predicted performance in accordance with the thickness deviation of utilized layers


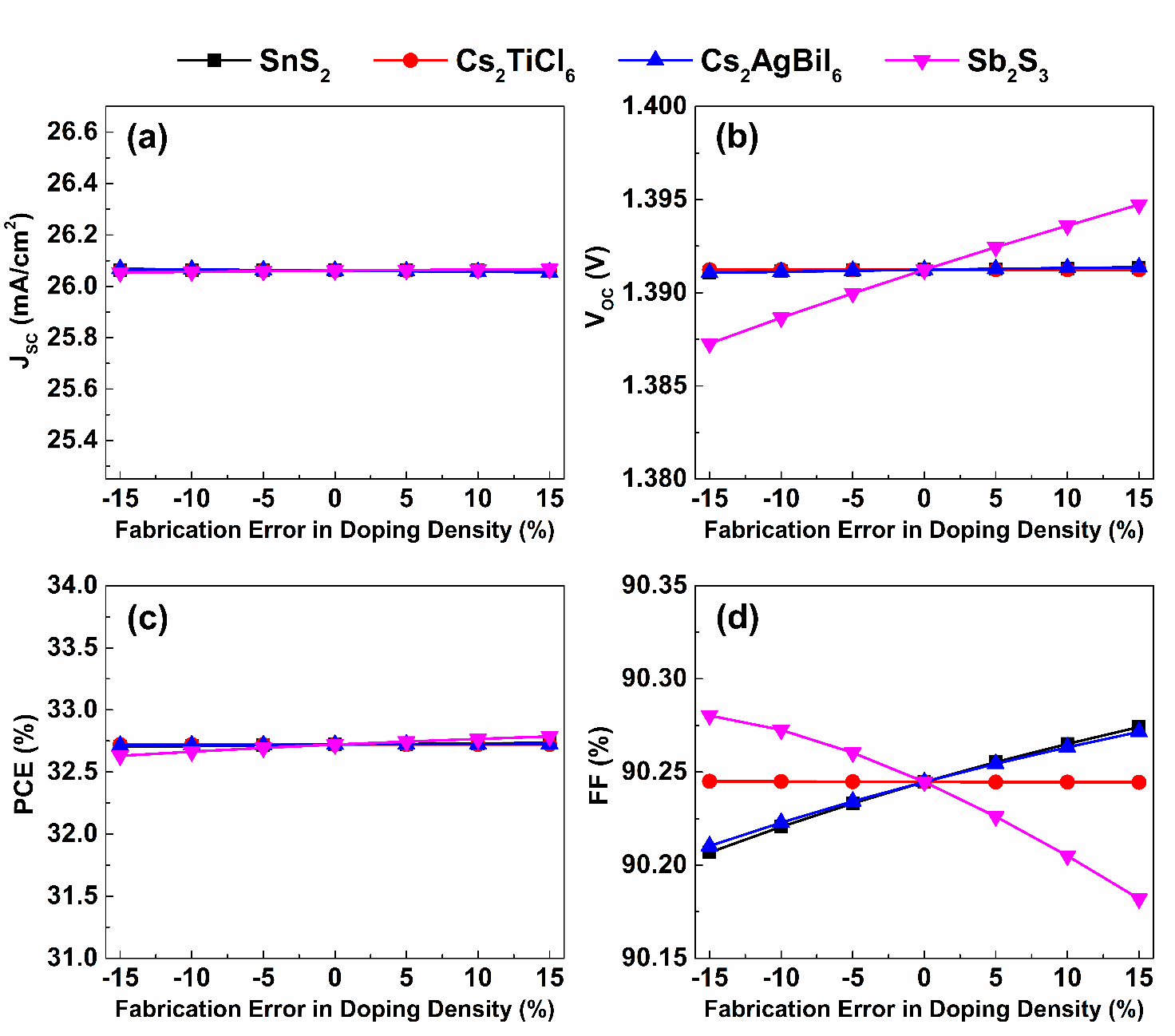


**Fig. S13** Variation of predicted performance in accordance with the doping density deviation of utilized layers


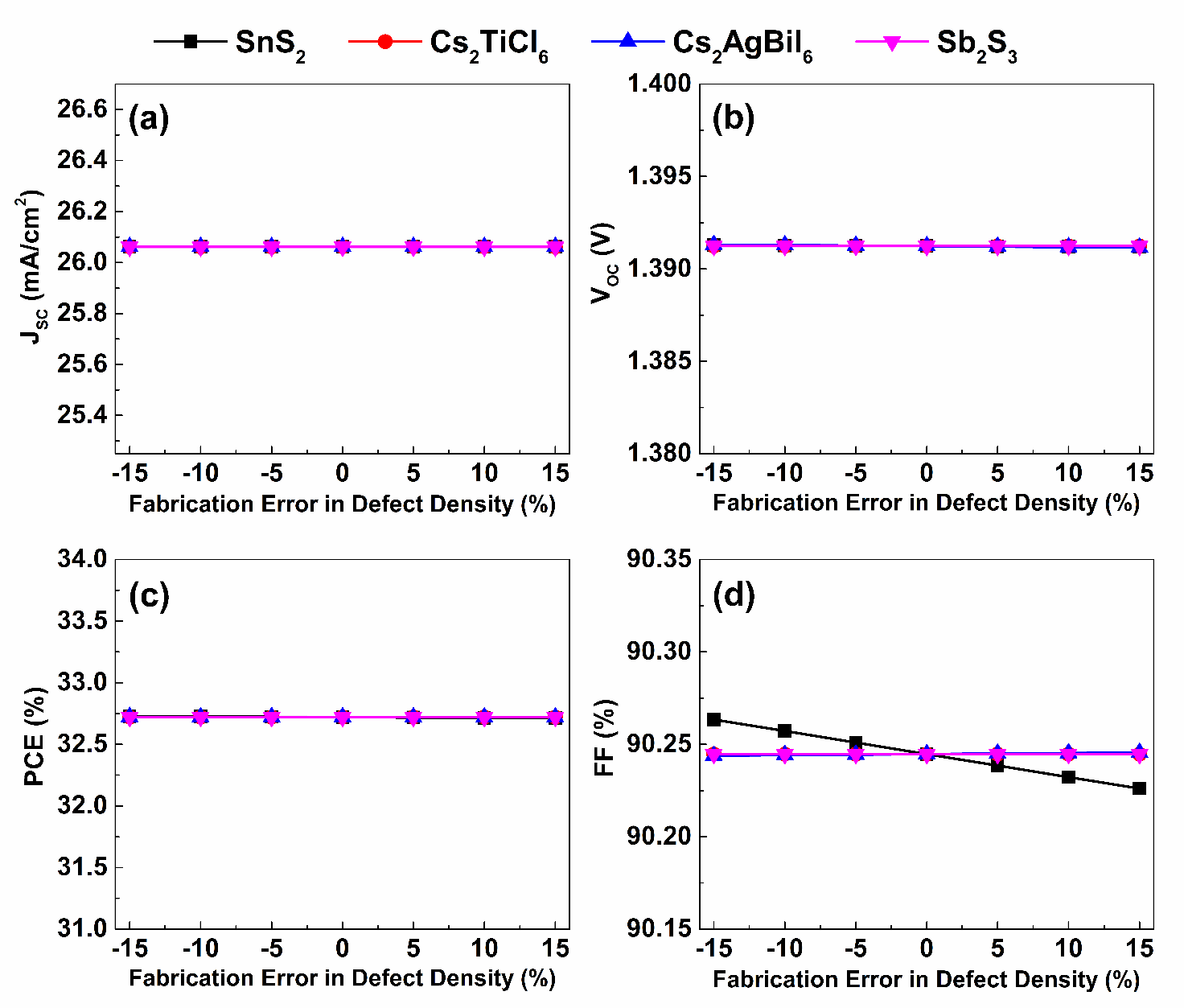


**Fig. S14** Variation of predicted performance in accordance with the defect density deviation of utilized layers

# **Reference**

1. Hossain, M. K. *et al.* Combined DFT, SCAPS-1D, and wxAMPS frameworks for design optimization of efficient Cs 2 BiAgI 6-based perovskite solar cells with different charge transport layers. *RSC Adv.* **12**, 35002–35025 (2022).

2. Das, A. *et al.* Numerical Simulation and Optimization of Inorganic Lead-Free Cs3Bi2I9-Based Perovskite Photovoltaic Cell: Impact of Various Design Parameters. *Energies* **16**, 2328 (2023).

3. Hossain, M. K. *et al.* An investigation of hole transport layers and electron transport layers to produce highly efficient K2TiI6-based perovskite solar cells. *Sci. Rep.* **15**, 19014 (2025).

4. Hossain, M. K. *et al.* Exploring the optoelectronic and photovoltaic characteristics of lead‐free Cs2TiBr6 double perovskite solar cells: a DFT and SCAPS‐1D investigations. *Adv. Electron. Mater.* **11**, 2400348 (2025).

5. Abbasi, Z., Jan, S. T., Safeer, M., Imran, M. & Rehman, A. U. Optimization & enhancement of KGeCl 3-based perovskite solar cells through charge transport layer engineering. *RSC Adv.* **15**, 2525–2544 (2025).

6. Hossain, M. K. *et al.* Design and simulation of CsPb. 625Zn. 375IBr2-based perovskite solar cells with different charge transport layers for efficiency enhancement. *Sci. Rep.* **14**, 30142 (2024).

7. Ali, M. E., Haque, M. M. & Cheragee, S. H. Device modeling and numerical analysis of lead-free MASnI3/Ca3AsI3 based perovskite solar cells with over 38% efficiency. *Sol. Energy* **288**, 113309 (2025).

8. Islam, M. A. & Paul, R. A lead-free inorganic Cs2TiX6-based heterostructure perovskite solar cell design and performance evaluation. *Opt. Quantum Electron.* **55**, 957 (2023).

9. Shimul, A. I., Hossain, M. M. & Dipa, S. A. Investigating the effectiveness of Ca3AsCl3-based Perovskite Solar Cells with optimal hole transport layer selection through numerical optimization and machine learning. *Opt. Commun.* **586**, 131916 (2025).

10. Moiz, S. A., Albadwani, S. A. & Alshaikh, M. S. Towards highly efficient cesium titanium halide based lead-free double perovskites solar cell by optimizing the interface layers. *Nanomaterials* **12**, 3435 (2022).

11. Feng, S. & Wang, J. Prediction of Organic–Inorganic Hybrid Perovskite Band Gap by Multiple Machine Learning Algorithms. *Molecules* **29**, 499 (2024).

12. Lu, X. *et al.* Efficient carbon electrode perovskite solar cells with robust buffer interfaces. *J. Mater. Res. Technol.* **24**, 8162–8170 (2023).

13. Fooladvand, P., Eskandari, M., Fathi, D. & Das, N. Single-walled carbon nanotube as hole transport layer in perovskite solar cell: Efficiency enhancement. *Energy Reports* **10**, 3652–3664 (2023).

14. Mohammed, K. A. M. *et al.* Improving the performance of perovskite solar cells with carbon nanotubes as a hole transport layer. *Opt. Mater. (Amst).* **138**, 113702 (2023).

15. Son, C., Son, H. & Jeong, B.-S. Enhanced Conversion Efficiency in MAPbI3 Perovskite Solar Cells through Parameters Optimization via SCAPS-1D Simulation. *Appl. Sci.* **14**, 2390 (2024).

16. Mehmood, S., Xia, Y., Qu, F. & He, M. Investigating the Performance of Efficient and Stable Planer Perovskite Solar Cell with an Effective Inorganic Carrier Transport Layer Using SCAPS-1D Simulation. *Energies* **16**, 7438 (2023).

17. Beriha, S., Dikhit, A. & Tripathy, S. K. Improving the efficiency of a FTO/PCBM/Cs2AgBiBr6/NiOx/Au lead-free double perovskite solar cell using numerical simulation through optimizing the absorption layer thickness and work function of electrodes. *Results Opt.* **12**, 100467 (2023).
